# Thymidylate synthase inhibitory drugs induce p53-dependent pathways differently

**DOI:** 10.1371/journal.pone.0332491

**Published:** 2026-07-01

**Authors:** Eszter Holub, Milda Blanka Szajkó, Anna Felföldi, Beáta G. Vértessy, Angéla Békési

**Affiliations:** 1 Department of Applied Biotechnology and Food Science, Faculty of Chemical Technology and Biotechnology at Budapest University of Technology and Economics, Budapest, Hungary; 2 Genome Metabolism Research Group, Institute of Molecular Life Sciences at HUN-REN Research Center for Natural Sciences, Budapest, Hungary; 3 Doctoral School of Biology, Institute of Biology at ELTE Eötvös Loránd University, Budapest, Hungary; BMSCE: BMS College of Engineering, INDIA

## Abstract

Thymidylate synthase (TS) is a key enzyme in thymidylate biosynthesis and an established target of chemotherapeutics such as 5-fluoro-2’-deoxyuridine (5FdUR) and raltitrexed (RTX). Inhibition of TS disrupts the dUTP:dTTP balance, leading to uracil misincorporation, triggering futile base excision repair cycles, DNA strand breaks, and ultimately cell death. Interestingly, when the main uracil-DNA repair pathway is inhibited, treatment with TS-inhibitory drugs still leads to cell death. Beyond its catalytic role, TS also binds RNA, autoregulating its own translation and interacting with transcripts such as p53 and c-Myc, thereby linking TS activity to broader post-transcriptional regulatory networks. These interactions, together with regulation by miRNAs and lncRNAs, suggest that TS inhibition may provoke cellular responses extending beyond DNA metabolism. To explore these mechanisms, we investigated the transcriptomic effects of TS inhibition with either 5FdUR or RTX in wild-type HCT116 and two HCT116-derived cell lines with different capacities in base excision and mismatch repair pathways. Both drugs induced DNA damage responses yet displayed distinct transcriptional signatures. A strong 5FdUR-biased induction of mRNAs corresponding to p53-related pathways was detected in all cell lines and further validated with qPCR and Western blot. Moreover, co-immunoprecipitation coupled to sequencing revealed direct RNA partners of TS, highlighting its possible post-transcriptional regulatory role. Our findings underscore the multifaceted impact of TS inhibition, linking enzymatic disruption to RNA-level regulation and revealing drug-specific differences in cellular responses.

## Introduction

Thymidylate synthase (TS, abbreviations are listed in S1 Table), a key enzyme in thymidylate biosynthesis, forms the sole *de novo* source of dTMP, a dTTP precursor, by transferring a methyl group from 5,10-methylenetetrahydrofolate (MTHF) to dUMP. Cancer cells are actively dividing, thus targeting nucleotide biosynthesis is a common anticancer strategy, and inhibition of TS is widely used in chemotherapeutic treatment of solid tumors such as colon, breast, and head and neck cancers [1–3]. TS is usually targeted by using substrate analogue molecules such as antifolates or fluoro-pyrimidines [2,4]. Inhibition of TS perturbs the cellular dUTP:dTTP pool, which can lead to elevated uracil incorporation upon DNA synthesis, triggering base excision repair (BER) [5,6]. This repair pathway is initiated by the uracil-DNA-glycosylases (UDGs), among which the uracil-N-glycosylase (UNG) has the highest activity [7]. If a high dUTP:dTTP ratio persists, the repair is transformed into hyperactive futile repair cycles, DNA strand breaks, and finally, cell death [1,6]. We have shown that combining UGI-driven inhibition of UNG [8] with the frequently used TS-inhibitory drugs 5-fluoro-2’-deoxyuridine (5FdUR) and raltitrexed (RTX) [9,10] leads to a dramatic increase in genomic uracil level, suggesting that these drugs efficiently perturb the cellular dUTP:dTTP ratio, resulting in thymine replacing uracil incorporation [11,12]. Importantly, the cytotoxicity remained even if U-DNA BER was severely impaired, which suggests additional mechanisms of action beyond the genomic instability caused by the putatively hyperactive repair [12,13].

The two TS-inhibitory drugs, the nucleotide analogue 5FdUR and the antifolate RTX, lead to either the formation of a covalent ternary complex [14], or a competitive inhibition of folate-binding [15]. The binding of these drugs assumes an active conformation of TS, which is usually in equilibrium with an inactive state [16]. This equilibrium is considered a pivotal factor in drug sensitivity; accordingly, a new group of TS inhibitors has also been designed, shifting this equilibrium towards the inactive conformation [17].

As global overexpression of TS has been associated with emerging drug resistance and a worse survival rate [18], understanding the complex regulation of TS expression, including transcriptional, post-transcriptional, and autoregulatory mechanisms, might be beneficial. The TS mRNA is negatively regulated by different microRNAs (miRNAs), which are targets of the MALAT1 long-noncoding RNA (lncRNA), usually upregulated in cancers [19], and their transcription is also repressed by p53 [20]. The TS mRNA is also the target of miR192/215-5p, which is induced by p53 [20].

Besides having a crucial enzymatic role, TS is also able to bind to its own mRNA, autoregulating its protein level by translation inhibition [21]. This phenomenon can provide a potential resistance mechanism against inhibitory drug treatments, as the binding of drugs to the active site interferes with the mRNA binding and translation inhibition [21–23]. Hence, the TS protein tends to release its mRNA, allowing it to be translated; thus, the newly formed enzyme can counteract the drug effect [24]. Interestingly, similar RNA-mediated autoregulatory mechanisms have been described for two other members of the folate cycle: the dihydrofolate reductase (DHFR) [25,26] and the serine hydroxymethyltransferase (SHMT1) [27,28], which were also shown to form a ternary complex with TS, potentially facilitating thymidylate biosynthesis [29].

TS can also bind to the mRNA of the tumor-suppressor, p53, and the oncogene, c-Myc [30–32]. TS might repress the translation of p53 mRNA [30]. Treatment with 5FU increased TS mRNA level and also increased protein level of TS and p53 in colon cancer cell lines, as well as in tumors, which was associated with worse patient survival [33].

In addition to TS, p53, and c-Myc mRNAs, a couple of more TS-bound RNAs have also been identified in H630-R10 colon cancer cells using immunoprecipitation of TS-containing hRNPs, molecular cloning, in vitro transcription, and screening for high-affinity binders [34]. Apparently, no sequence homology was found among these isolated RNA fragments of p53, ISG15, ZNF460, KIF5B, and 7S RNA, or TS. Among these RNA sequences, TS and p53 mRNA binding sites were studied in detail [30,35] and found that binding relies on RNA secondary structures rather than specific sequence elements, suggesting a more general RNA regulatory role of TS with the potential of yet unidentified RNA binding partners. Beyond the known mRNA targets, different noncoding RNAs might also be affected by TS-binding, as well as by transcriptional changes induced by TS-inhibitory drug treatments. These studies suggest a complex regulatory network around TS, which might be influenced by inhibitory drug treatments.

To obtain molecular details of cellular responses, whole transcriptome analysis might be revealing. Above the protein-coding mRNAs, the functional relevance of the noncoding transcriptome has also been established. Short noncoding RNAs (<200–300 nucleotides) include small nuclear RNAs (snRNAs), small nucleolar RNAs (snoRNAs), and miRNAs. SnoRNAs primarily guide rRNA modification and maturation [36] and miRNAs mediate gene silencing through mRNA degradation or translational inhibition [37,38]. The functions of other small RNAs, such as snRNAs, are more diverse. lncRNAs, in contrast, are longer transcripts that do not encode proteins but regulate gene expression at multiple levels: transcriptionally, by modulating chromatin structure, and post-transcriptionally, often by sponging miRNAs to prevent degradation of their target mRNAs [39–41]. They may also act as scaffolds in phase separation [41,42].

Previously, in UNG-inhibited HCT116 colon adenocarcinoma cell-lines either with impaired or restored mismatch repair (MMR), we published that both 5FdUR and RTX treatments result in an S-phase arrested phenotype, and a similar increase of γH2AX signal [12], which suggests that even though the main UDG is inhibited and genomic uracil content dramatically increased without efficient repair, these treatments eventually lead to the occurrence of DNA-strand breaks and the initiation of DNA damage response (DDR) signaling [12]. Recently, we also detected differences between the effects of the two drugs reflected in altered genomic uracil distributions, drug sensitivity, and induced mutagenicity [13]. Surprisingly, 5FdUR, when applied at high doses, was less effective than at lower doses, or than RTX at any dose in UNG-inhibited HCT116 cells, regardless of their MMR status [13]. In MMR-deficient cells, this decreased sensitivity is accompanied by an increased frequency of genomic C-to-T transitions due to the selective induction of cytidine deaminases in response to high-dose 5FdUR treatment [13]. Despite similarly decreased sensitivity and induced cytidine deaminases, the increase in genomic C-to-T transitions was not detected in MMR-proficient cells, most likely due to their efficient repair coupled with extensive DNA synthesis that was reflected in a markedly divergent genomic uracil profile [13]. We propose that TS inhibition can trigger more complex cellular responses than previously thought, and drug-specific differences might also occur at transcriptomic and proteomic levels.

In the present study, we aimed to provide a better understanding of the mechanism of action of these two drugs and the potential causes of the obvious differences between them. Hence, we performed whole-transcriptome sequencing from HCT116 cells with altered DNA repair capacities, applying the same 48-hour treatments as in previously published studies. We identified significantly upregulated and downregulated groups of RNAs, with some drug-specific differences. Most prominently, the effects on the p53-regulated pathways were markedly altered upon the two drug treatments, especially in DNA-repair-deficient cell lines, to which validation qPCR and Western blotting experiments were also performed. The RNA-based regulatory role of TS was addressed using a TS-coupled RNA-co-immunoprecipitation (TS-RIP) followed by sequencing (abbreviated as TS-RIP-seq) from DNA-repair-deficient cells, which revealed a much wider repertoire of target RNAs than expected, and drug-specific differences could also be identified.

## Results

### RTX and 5FdUR treatments influence cellular gene expression profiles differently

To characterize the differences in the cellular effects of the two TS inhibitory drugs, RTX and 5FdUR, we performed whole transcriptome sequencing of (i) the HCT116-UGI cell line, in which both UNG-initiated base excision repair and MMR are impaired; (ii) the MMR-proficient but UNG-inhibited HCT116-MMR-UGI cell line; and (iii) the parental MMR-deficient HCT116 cell line (data are available at GEO under accession GSE318306). Relative expression levels and differential expression were determined following 48-hour treatments with either 0.1 µM RTX or 20 µM 5FdUR, to be comparable with the earlier results [12,13] (cf. Materials and Methods and Supplementary Methods).

**Fig 1:**
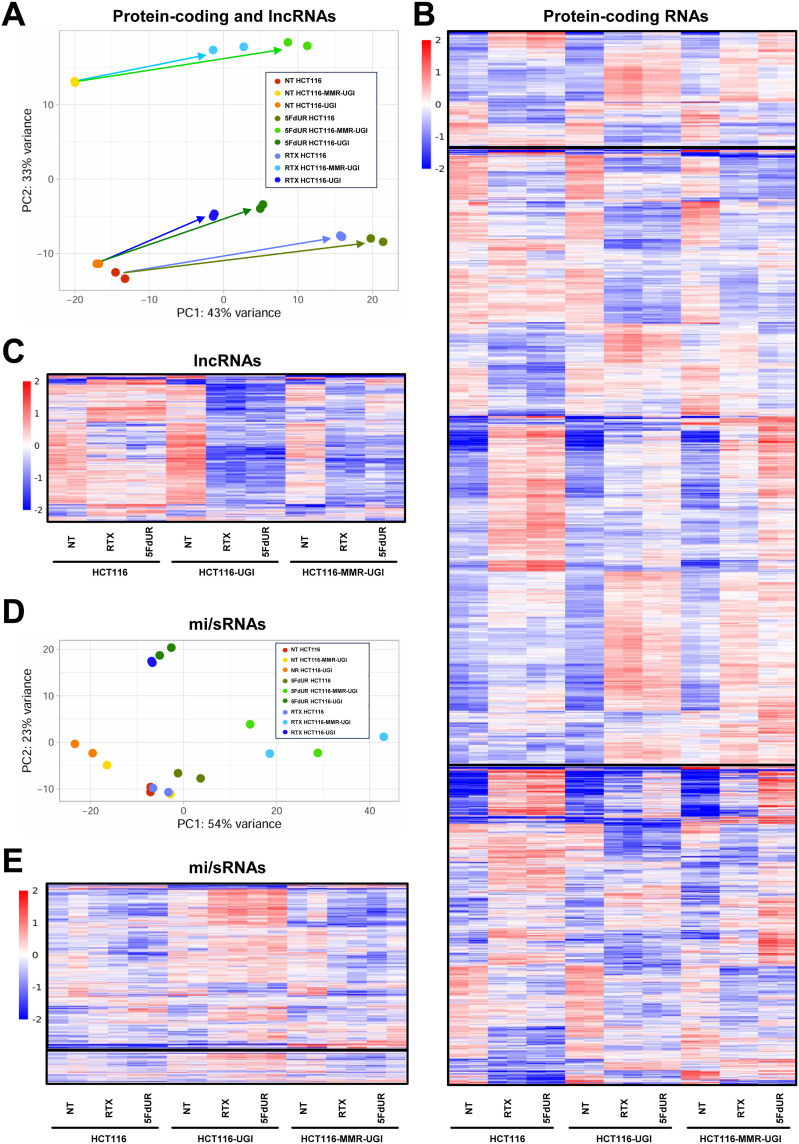
Summary of whole-transcriptome sequencing data. Two biological replicates of the three cell lines (HCT116, HCT116-UGI, and HCT116-MMR-UGI), non-treated (NT) or treated with either 0.1 μM RTX or 20 μM 5FdUR, overall 18 samples were sequenced and compared. (A) Principal component analysis (PCA) with sequencing data of long RNAs, including protein-coding and lncRNAs as defined in GenCode V43 [43,44]. Arrows represent the shifts upon drug treatments (RTX or 5FdUR) compared to the non-treated (NT) state. Color codes are given and consequently applied throughout the study. Dots with the same color represent biological replicates of the same treatment and cell line combination. (B) Relative expression levels (log_2_(FPKM_sample_ / FPKM_mean_)) of protein-coding genes that show significant differential expression at least in one comparison of samples. The heatmap panels (separated with solid black lines) represent three ranges of expression levels: FPKM ≥ 100,000 (top), 100,000 > FPKM ≥ 10,000 (middle), and 10,000 > FPKM ≥ 1000 (bottom). Genes with a mean FPKM below 1000 were excluded from the analysis. The color bar (right) indicates changes of the (log_2_(FPKM_sample_ / FPKM_mean_)) from −2 (blue) to 2 (red); larger changes are represented with the same blue and red. (C) Relative expression levels of lncRNAs that reach a minimum expression (mean FPKM≥1000) and display significant differential expression at least in one comparison of samples. The heatmap representation follows the same rules as on panel B. (D) PCA on count data of short RNAs, including sRNAs, snRNAs, snoRNAs (as defined in GenCode V34 annotation), and matured miRNAs (as defined in MiRBase, [45,46]). (E) Relative expression levels of short RNAs that reach a minimum expression (mean count ≥ 10). Heatmaps were generated separately for miRNAs (top) and smallRNAs (bottom). The color bar (right) indicates changes of the (log_2_(RPKM_sample_ / RPKM_mean_)) from −2 (blue) to 2 (red); larger changes are represented with the same blue and red.

In whole-transcriptome sequencing, long and short RNAs are size-separated (with a 200 nt cutoff) and processed separately, from library preparation to data analysis. Accordingly, we refer to these datasets as long RNA and short RNA throughout this study. Principal component analysis (PCA) of long RNA expression levels revealed clear drug-induced differences in the transcriptional profiles, as well as differences arising from the genetic background (Fig 1A). The non-treated MMR-deficient samples (NT HCT116 and NT HCT116-UGI) clustered closely together, whereas non-treated HCT116-MMR-UGI cells, in which MMR is restored but BER remains impaired, displayed a pronounced shift. The differences induced by RTX or 5FdUR were slightly more pronounced in the HCT116 and HCT116-MMR-UGI cell lines, compared with HCT116-UGI cells. Nevertheless, the two treatments also produced distinct transcriptional shifts; RTX generally induces smaller changes than 5FdUR (Fig 1A).

To visualize differences in the relative expression of individual genes across the 18 samples, heatmaps were generated separately for protein-coding genes (Fig 1B) and lncRNAs (Fig 1C) as defined in GenCode V34. To avoid presenting excessive and uninformative data, genes were filtered to include those with a mean expression level of FPKM > 1,000 that were significantly differentially expressed upon drug treatment in at least one condition. Protein-coding RNAs were further subdivided into three expression ranges (FPKM ≥ 100,000; 100,000 > FPKM ≥ 10,000 and 10,000 > FPKM ≥ 1000), highlighting the impact of relative expression changes (Fig 1B). The resulting expression profiles of non-treated samples were similar, showing only minor differences among the three cell lines, with HCT116-MMR-UGI being the most divergent, consistent with the PCA results (cf. Fig 1A). Drug treatments, however, altered expression profiles in a manner that depended on the uracil-DNA repair capacity of the cells. Distinct clusters of genes were selectively up- or downregulated in HCT116 or HCT116-UGI cells. Smaller but consistent differences between corresponding RTX- and 5FdUR-treated samples were also observed within each expression range. Notably, these drug-specific differences were more pronounced in HCT116-MMR-UGI cells, in which the RTX-induced expression profile more closely resembled that of HCT116-UGI cells, whereas the 5FdUR-induced changes were closer to those observed in HCT116 cells with active uracil-DNA repair.

While for protein-coding RNAs, a balanced amount of drug-induced down- and upregulated genes were found in all cell lines (Fig 1B), the lncRNAs exhibited a strong bias towards downregulation in cells with inhibited UNG (HCT116-UGI and HCT116-MMR-UGI), especially when neither BER nor MMR was functioning (Fig 1C). In contrast, in HCT116 cells with active uracil-DNA repair and low genomic uracil content, lncRNA expression displayed smaller and more balanced changes (Fig 1C). Similar to protein-coding RNAs, certain drug-specific differences in lncRNAs’ expression profiles were also observed.

The PCA of short RNAs, denoted as mi/sRNAs – including sRNAs, snRNAs, snoRNAs (cf. GenCode V34 annotation), and matured miRNAs (cf. MiRBase), revealed major shifts due to drug treatments that are also influenced by the cell line background (Fig 1D). However, while the biological replicates were closely clustered in the case of long RNAs, short RNA expression profiles show great variance, especially in the case of HCT116-MMR-UGI cells. Interestingly, the smallest drug-induced changes of mi/sRNAs’ expression were observed in the case of HCT116 cells, where the biggest change of long RNAs’ expression was detected (Fig 1D). Comparing the relative expression levels (RPKM) of mi/sRNA genes (miRNAs and other short RNAs plotted separately) revealed differences between cell lines, and the effect of drug treatments also varies based on the cellular background (Fig 1E). Notably, the inter-replicate variability is also higher than in the case of the long RNA analysis, consistent with the PCA results. Differential expression was further analyzed separately for protein-coding (mRNAs) and later for lncRNAs and short RNAs (mi/sRNAs).

### Differentially expressed (DE) protein-coding genes following RTX and 5FdUR treatment in the three applied cell lines

**Fig 2:**
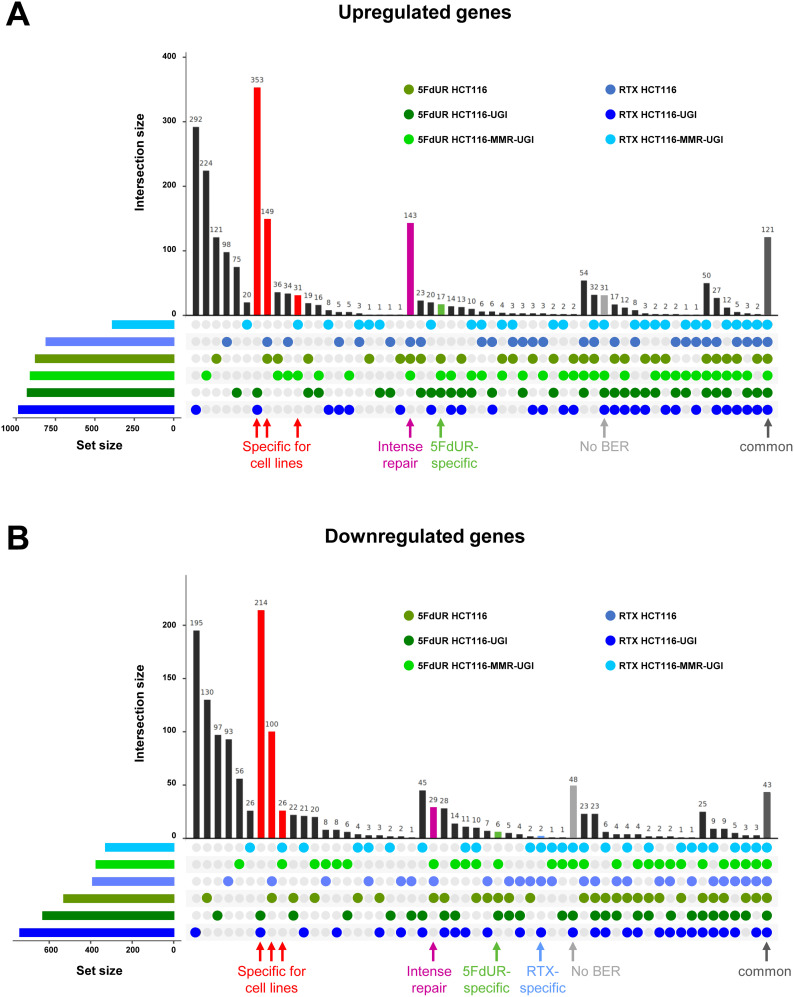
Differentially expressed (DE) protein-coding genes following RTX and 5FdUR treatments across the three cell lines. Treatment-induced differential expression profiles were calculated from long RNA sequencing data of the overall 18 samples (for names and the color code see Fig 1). For significance, data were filtered: absolute fold change ≥ 2, q-value < 0.05, and mean FPKM > 1000. Multiple comparisons of significantly upregulated (A) and downregulated (B) protein-coding genes are displayed on UpSet plots [47]. Set size shows the number of DE genes in the given conditions. The intersection size represents the number of DE genes common to the conditions indicated by the colored dots below the bar graph. Intersections without common DE genes are not shown. Some specific intersections are marked: specific to cell lines (red bars and arrows); specific to drugs (5FdUR: green, RTX: blue); the UNG-inhibited cell lines (gray bars and arrows, labeled as “NO BER”); those cell lines where extensive repair synthesis is expected (HCT116 and 5FdUR-treated HCT116-MMR-UGI, pink bars and arrows), and the common up- or downregulated are also indicated (dark gray bars and arrows for the last intersection).

DE mRNAs were characterized for each drug-treated sample as compared to the corresponding NT controls. A comparison of DE mRNAs across the six conditions (different cell lines and treatments) is presented in UpSet plots (Fig 2) as well as in volcano plots (S1 Fig). In general, more DE mRNAs were found to be upregulated than downregulated after drug treatments (cf. Set size in Fig 2 and S1 Fig). In each cell type, the two treatments resulted in a certain amount of common DE mRNAs (S1 Fig). Despite the major cell-line specific differences, a group of absolutely common DE mRNAs was also identified, meaning 121 upregulated and 43 downregulated mRNAs, which implies expected common aspects of the cellular responses.

### Drug-induced expression changes confirm S-phase-arrest DNA damage-response, and impaired transcriptional activity

To understand the induced and the attenuated processes and pathways involved in this core cellular response, a Gene Set Enrichment Analysis (GSEA) was performed on these commonly upregulated and downregulated groups of protein-coding genes using the online platform of the STRING database ([48], Table 1)). The 121 commonly upregulated mRNAs form a highly interconnected network with enriched terms including p53-related functions, DNA damage response (DDR), and localization in the extracellular space (S2 Fig). Notably, several of the most interconnected upregulated genes are highly abundant already in the non-treated cells (reflected in node size in S2A Fig). In contrast, the 43 genes that were downregulated in response to both RTX and 5FdUR in each cell line did not exhibit notable interconnectivity or functional enrichments (Table 1).

**Table 1:** Network statistics and permanent links to STRING networks and GSEA.

				Number of nodes	Number of edges	Average node degree	Avg. clustering coefficient	Expected number of edges	PPI enrichment p-value	Permanent link
Commonly upregulated in all six conditions				121	99	1.64	0.285	43	1.63e-13	https://version-12-0.string-db.org/cgi/network?networkId=bi45kG4Q0PmZ
Commonly downregulated in all six conditions				43	6	0.279	0.233	4	0.183	https://version-12-0.string-db.org/cgi/network?networkId=bGKkbMeIpToI
Specific for HCT116		Drug-induced upregulation		149	34	0.456	0.25	25	0.0415	https://version-12-0.string-db.org/cgi/network?networkId=bwhAqxR9tcck
		Drug-induced downregulation		100	42	0.84	0.297	34	0.0952	https://version-12-0.string-db.org/cgi/network?networkId=bu2KiPXza9GX
Specific for HCT116-UGI		Drug-induced upregulation		353	2201	12.5	0.47	853	<1.0e-16	https://version-12-0.string-db.org/cgi/network?networkId=bFhnWZG9CxPt
		Drug-induced downregulation		213	70	0.657	0.317	38	2.63e-06	https://version-12-0.string-db.org/cgi/network?networkId=btCRHReuKyyt
Specific for HCT116-MMR-UGI		Drug-induced upregulation		31	9	0.581	0.247	2	0.000828	https://version-12-0.string-db.org/cgi/network?networkId=bCebsv13lsSS
		Drug-induced downregulation		26	9	0.692	0.269	3	0.00457	https://version-12-0.string-db.org/cgi/network?networkId=bFSErsBZxvdb
**RTX**	Upregulated in all three cell lines			140	135	1.93	0.318	61	3.33e-16	https://version-12-0.string-db.org/cgi/network?networkId=b1THuVUPp1Y7
	Downregulated in all three cell lines			66	15	0.455	0.288	8	0.0124	https://version-12-0.string-db.org/cgi/network?networkId=bNZL6wvDNKjK
**5FdUR**	Upregulated in all three cell lines			296	453	3.06	0.381	243	<1.0e-16	https://version-12-0.string-db.org/cgi/network?networkId=bcOtZqhRtXRF
	Downregulated in all three cell lines			96	25	0.521	0.202	15	0.016	https://version-12-0.string-db.org/cgi/network?networkId=bvQph3CcSiRu
**HCT116**	Up-regulated genes		common	629	1196	3.8	0.361	737	<1.0e-16	https://version-12-0.string-db.org/cgi/network?networkId=bhWuEyiMWDLB
			RTX-spec	174	78	0.897	0.293	48	5.9e-05	https://version-12-0.string-db.org/cgi/network?networkId=bo4yIVHSL0NP
			5FdUR-spec	247	194	1.57	0.287	155	0.00128	https://version-12-0.string-db.org/cgi/network?networkId=b4fgb8T18kBx
	Down-regulated genes		common	266	426	3.2	0.400	230	<1.0e-16	https://version-12-0.string-db.org/cgi/network?networkId=bhy4Wq9kPUhK
			RTX-spec	122	37	0.607	0.223	37	0.508	https://version-12-0.string-db.org/cgi/network?networkId=bRgwaMBmp8DC
			5FdUR-spec	260	295	2.27	0.330	192	2.6e-12	https://version-12-0.string-db.org/cgi/network?networkId=bfcrU88pUUBd
**HCT116-UGI**	Up-regulated genes		common	644	4373	13.6	0.432	2219	<1.0e-16	https://version-12-0.string-db.org/cgi/network?networkId=bKKqauOiNRJ9
			RTX-spec	328	959	5.85	0.403	570	<1.0e-16	https://version-12-0.string-db.org/cgi/network?networkId=bDQI9Eg8bgVx
			5FdUR-spec	274	273	1.99	0.346	187	1.9e-09	https://version-12-0.string-db.org/cgi/network?networkId=bLv5J1o4asqw
	Down-regulated genes		common	479	405	1.69	0.353	291	1.66e-10	https://version-12-0.string-db.org/cgi/network?networkId=bbKspCbRd8dC
			RTX-spec	258	112	0.868	0.324	71	3.67e-06	https://version-12-0.string-db.org/cgi/network?networkId=bzqQMAG6QiWW
			5FdUR-spec	149	41	0.55	0.244	29	0.0187	https://version-12-0.string-db.org/cgi/network?networkId=bAR9RQGpkE9j
**HCT116-MMR-UGI**	Up-regulated genes		common	313	551	3.52	0.360	284	<1.0e-16	https://version-12-0.string-db.org/cgi/network?networkId=bhhjmzp6cZ2p
			RTX-spec	71	116	3.27	0.398	25	<1.0e-16	https://version-12-0.string-db.org/cgi/network?networkId=bNazrj0dHW7l
			5FdUR-spec	591	1039	3.52	0.359	670	<1.0e-16	https://version-12-0.string-db.org/cgi/network?networkId=bVUXZG0tssRb
	Down-regulated genes		common	203	420	4.14	0.416	180	<1.0e-16	https://version-12-0.string-db.org/cgi/network?networkId=bSXun4XW4518
			RTX-spec	125	24	0.384	0.238	14	0.00742	https://version-12-0.string-db.org/cgi/network?networkId=bfuREDofA1h8
			5FdUR-spec	168	156	1.86	0.330	66	<1.0e-16	https://version-12-0.string-db.org/cgi/network?networkId=bf18qLVhnnPO

Significantly up- or downregulated genes were those with abs(log_2_FC) > 1; q < 0.05; and mean FPKM ≥ 1000. Interactions in the STRING database were filtered for “medium confidence level”, and only “textmining”, “experiments”, and “databases” were considered as valid sources. The whole genome was chosen as the statistical background.

In addition to this core group of commonly upregulated genes, numerous genes were up- or downregulated in limited sets of the six applied conditions (cf. Fig 2 and S1 Fig). The most populated gene groups on the UpSet plots were the double intersections of up- and downregulated genes in HCT116-UGI cells with both treatments (353 and 214 genes in Fig 2A, B, respectively). The upregulated genes form a highly interconnected network with numerous enriched functional terms, including those related to DNA synthesis, chromatin organization, and unfolded proteins (cf. permanent link to STRING analysis in Table 1). In contrast, the corresponding downregulated genes were less interconnected, and only “Zn-finger domains” and “nuclear pore complex interacting protein domains” were enriched. Other double intersections specific to cell lines were also pronounced (red arrows in Fig 2), suggesting that the cellular background has a major effect on the overall transcriptomic changes after these two TS-inhibitory treatments. Therefore, we performed GSEA on commonly up- or downregulated protein-coding genes in each cell line separately (S3A and S3B Fig, cf. also Table 1 and Venn diagrams in S1 Fig). The enrichment data were collected and visualized for the three cell lines together (S3A and S3B Fig).

The common upregulated DE protein-coding genes displayed enrichment for terms corresponding to extracellular region, apoptosis, chromosomal instability, DNA damage response, and p53-related pathways in all three cell lines (S3A Fig), which is in agreement with the GSEA results of the 121 commonly upregulated genes in all six conditions (cf. S2B Fig).

In contrast, the DE protein-coding genes commonly downregulated by both drug treatments were enriched in terms corresponding to cellular senescence, DNA repair, transcription regulation, and chromatin organization (S3B Fig). Interestingly, despite numerous downregulated genes in the DNA-repair-deficient HCT116-UGI cells (cf. Venn diagrams in S1 Fig, and red arrows in Fig 2B), only transcription-regulation-related terms were enriched (S3B Fig). Consistently, GSEA performed on genes downregulated by both drugs exclusively in HCT116-UGI cells (214 genes, cf. Fig 2B) exhibited enrichment for Zn-finger domains typically occurring in transcription factors (cf. permanent link in Table 1).

Notably, the 143-member peak standing out from the triple intersections of upregulated genes (Fig 2A) belongs to the drug-treated HCT116 and the 5FdUR-treated HCT116-MMR-UGI samples. Based on our previous results, the common aspect of these three conditions is likely extensive DNA repair synthesis, either UNG-initiated BER in RTX- or 5FdUR-treated HCT116 or MMR triggered by the increased number of U:G mispairs in 5FdUR-treated HCT116-MMR-UGI cells [12,13].

### Drug-specific differences in DE mRNAs

Interestingly, while groups of DE mRNAs specific to cell lines are populated, the absolute drug-specific responses observed in each of the three cell lines are less pronounced, with 17 up-, and 6 downregulated, and only two downregulated genes for 5FdUR and RTX treatments, respectively (Fig 2). Hence, drug-specific differences were investigated in each cell line independently. GSEA performed on groups of RTX-specific upregulated genes (cf. Venn diagrams in S1 Fig) resulted in enriched terms related to cell cycle, cell cycle checkpoints, and DNA synthesis in the cases of the two UGI-expressing cell lines (S3C Fig). Elements of the p53 signaling pathway and p53 transcriptional network were still identified among the RTX-specific upregulated genes, but only in the case of HCT116-MMR-UGI cells (S3C Fig). The affected genes are mainly involved in transcription (e.g., transcription factors, RNA polymerase subunits, helicases) and cell cycle regulation (cyclins and cyclin-dependent kinases). Interestingly, among RTX-specific upregulated genes in HCT116 cells, “extracellular matrix organization” appeared as the only enriched term (S3C Fig). The enrichment analysis of the group of 5FdUR-specific upregulated genes revealed terms corresponding to MAPK, Nuclear receptors, and Vitamin D signaling pathways (S3D Fig). Additionally, the “p53 signaling pathway” and “p53 transcriptional gene network” were also enriched terms in the cases of HCT116-UGI and HCT116-MMR-UGI cells. These genes were mainly related to DNA damage (DRAM, POLH), cell-cell communication, and cell growth and death (WNTs, laminins, BTG2, BAX, BAK, FAS) (cf. Source Data File 1).

Consistent with the previous GSEA, the drug-specific groups of downregulated genes were not enriched in functional terms, or only a few were detected (cf. permanent links to STRING networks in Table 1).

**Fig 3:**
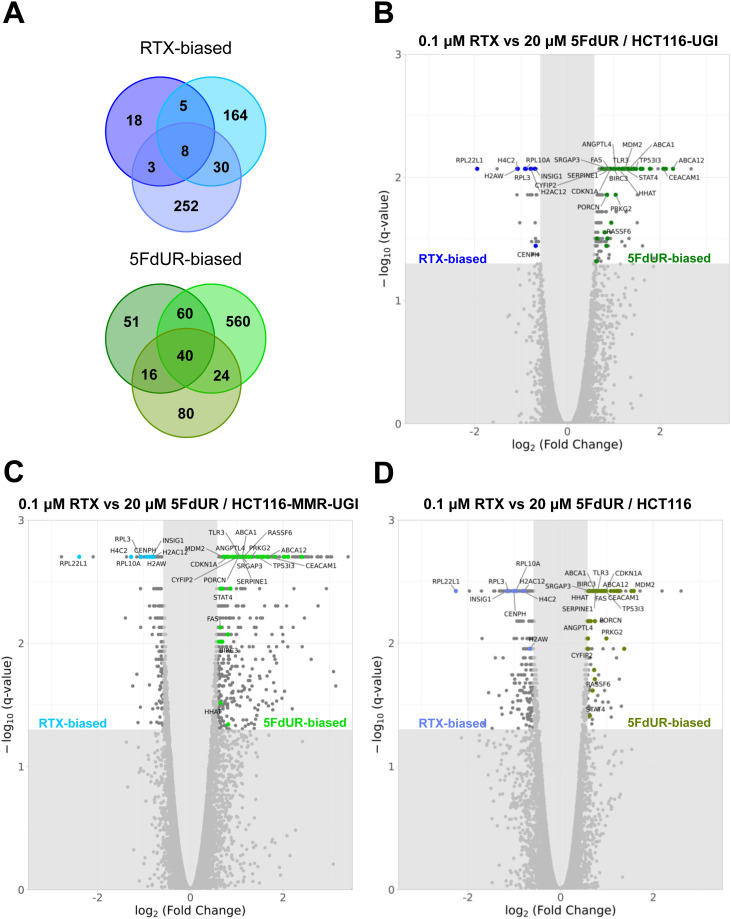
Protein-coding genes exhibiting drug-biased expression. Direct comparisons of the two drug treatments were performed for the three cell lines using the Cufflinks package [49,50]. For significance, differential expression data were filtered using the following criteria: absolute fold change ≥ 1.5, q-value < 0.05, and mean FPKM > 1000. (A) Venn diagram of RTX-biased (blue, top) and 5FdUR-biased (green, bottom) genes in the three cell lines. Volcano plots of drug-specific expression (B) in HCT116-UGI cells, (C) in HCT116-MMR-UGI cells, and (D) in HCT116 cells. The significant drug-biased genes are colored with dark gray on a white background, and those with drug-biased expression in all three cell lines are colored by the previously applied color code in each Volcano plot. The 8 common RTX-biased genes and selected ones of the 40 common 5FdUR-biased genes are labeled by their gene symbols.

To see another aspect of the drug-specific differences, we directly compared the two drug-treated samples on each cellular background (Fig 3). We found a general 5FdUR-biased expression of protein-coding genes in the UGI-expressing cell lines (Fig 3A). In contrast, an RTX-biased expression was observed in the original HCT116 cells, where incorporated uracils are continuously repaired by UNG-initiated BER, hence the genomic uracil level is kept low. Genes with drug-biased expression are also demonstrated on Volcano plots (Fig 3B-D), suggesting potential pathways selectively activated by 5FdUR- or RTX-treatments.

**Fig 4:**
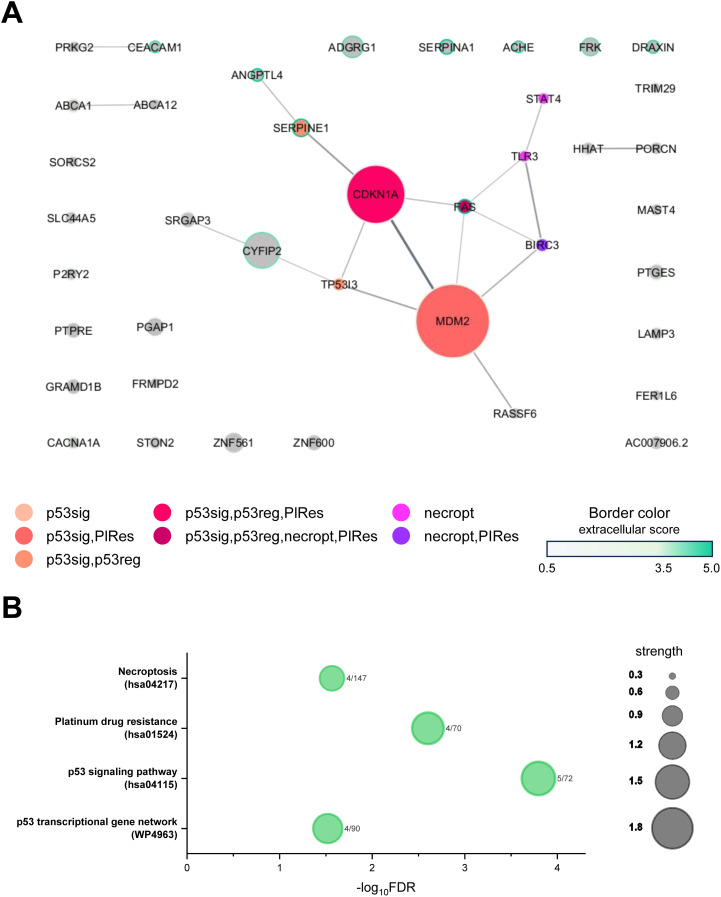
Network of the 40 genes that showed 5FdUR-biased expression in each of the three cell lines. **(A)** Network data from the STRING database visualized in CytoScape [51]. The mean FPKM values measured in non-treated samples were mapped to the node size, ranging from the smallest FPKM of 264 (FRMPD2) to the highest of 264940 (MDM2). Enriched functional terms were mapped to the node colors as indicated: p53sig – “p53 signaling pathway” (hsa04115) in KEGG Pathways, p53reg – “p53 transcriptional gene network” (WP4963) of WikiPathways, PlRes – “Platinum drug resistance” (hsa01524), and Necr – “necroptosis” (hsa04217) in KEGG Pathways. The border color was mapped to the STRING score for extracellular localization as indicated on the bar. **(B)** GSEA results for the 40 commonly 5FdUR-biased genes. GSEA was performed using the online Analysis tool of the STRING database, setting the whole genome as a background. Selected enriched terms (the same as on panel A, vertical axis) and the corresponding -log_10_(FDR) values (horizontal axis) are plotted on the enrichment maps. The dot size is mapped to the enrichment strength as indicated. The number of genes associated with each term in the current network is indicated relative to the total number of genes annotated with that term. The gene lists and all details are provided in Source Data File 1.

The selective enrichment of cell cycle and replication-related processes among RTX-induced genes, as well as of additional signaling pathways and DDR among 5FdUR-induced genes, reports differences in the cell response after treatment with these two TS inhibitors. This is further confirmed by the altered induction of p53-related genes following the two drug treatments, as also reflected in the enrichment of p53-related pathways within the 40 common 5FdUR-biased genes (Fig 4).

### Altered activation of the p53 regulatory network upon RTX and 5FdUR treatments

**Fig 5:**
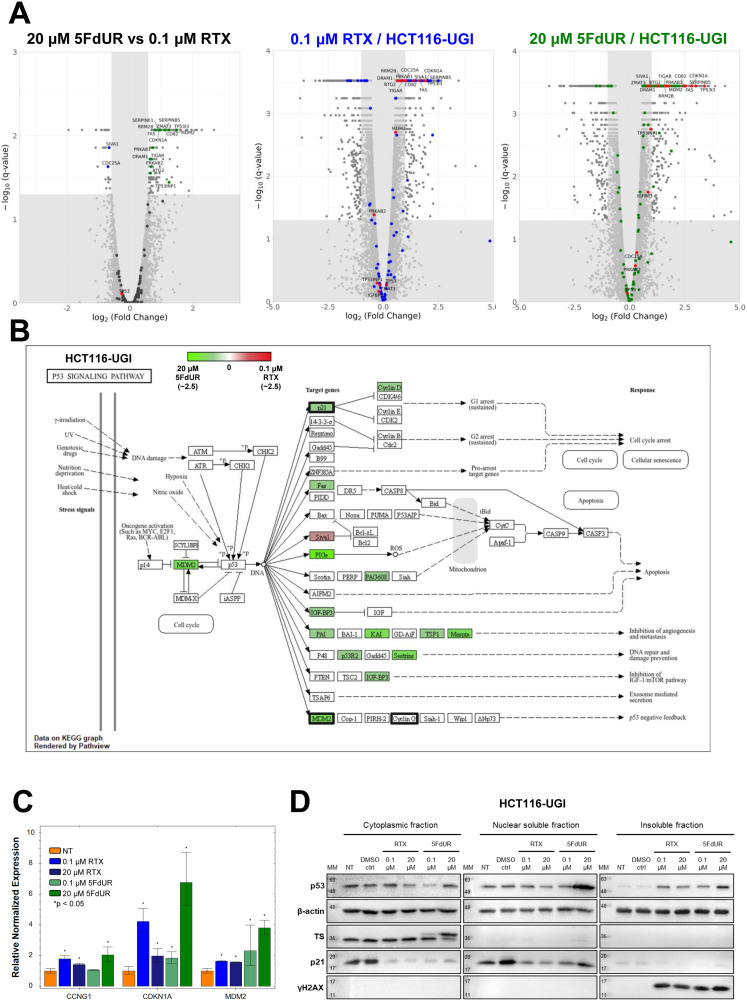
The p53 pathways are more strongly induced upon 5FdUR treatment. **A:** On the volcano plots (same as in Fig 3B and S1A Fig), components of the “p53 signaling pathway” (KEGG Pathways, hsa04115) and the “p53 transcriptional gene network” (WikiPathways, WP4963) are marked. Genes that show significant differences in the direct comparison of RTX and 5FdUR (left plot) are labeled with their gene symbols on all three plots. Color code on left panel: blue, RTX-biased expression; green, 5FdUR-biased expression; dark gray, p53 network components without significant differential expression. Color code on the middle and right panel: blue, p53 network components in RTX; green, p53 network components in 5FdUR sample; red, p53 network genes that are significantly different between RTX and 5FdUR. TP53 is highlighted in red on all three plots. **B:** Drug-specific differential expression levels are projected onto the “p53 signaling pathway” (KEGG Pathways, hsa04115) using clusterProfiler (R package) https://github.com/YuLab-SMU/clusterProfiler. The color bar (green to red) at the top represents log_2_(fold change) up to ±2.5. Genes measured by RT-qPCR are marked with a bold border. **C:** Induction of CCNG1 (cyclin **G)**, CDKN1A (p21), and MDM2 at the mRNA level, measured by RT-qPCR in the non-treated (NT) HCT116-UGI cells and after 48 h treatment with 0.1 or 20 μM 5FdUR or 0.1 or 20 μM RTX (*p < 0.05, at least two biological replicates). Data are provided in Source Data File 2. **D:** Expression and subcellular localization of p53, TS, p21, and γH2AX proteins in control samples (NT and DMSO control) and after treatments with TS inhibitory drugs (0.1 or 20 µM RTX or 5FdUR) in HCT116-UGI cells. Original blot images are provided in Source Data File 3.

First, p53-regulated genes and *TP53* itself were highlighted on the volcano plots, also labeling those genes that show significant differences in the RTX and 5FdUR comparison (Fig 5A and S4 Fig). Interestingly, *TP53* is not induced at the mRNA level in any of the cell lines; however, many of its target genes are significantly induced and show 5FdUR-biased expression. p53-mediated regulation is usually initiated within the DDR, and, while determining cell death or survival, it has multiple possible outputs [52–54]. We mapped the differential expression levels (RTX compared to 5FdUR) onto the network of “p53 signaling pathway” (hsa04115) from the KEGG Pathways database (Fig 5B and S5 Fig). In all three cell lines, a pronounced 5FdUR-biased induction of the p53 signaling network can be observed. It is immediately apparent that apoptosis-related subpathways differ between the two drugs: induction of reactive oxygen species (ROS) may play a greater role in 5FdUR-mediated cytotoxicity, which is even more pronounced in HCT116 and HCT116-MMR-UGI cells. In contrast, Siva-1 – encoded the only RTX-biased gene displayed in the KEGG network – inhibits the anti-apoptotic Bcl-2 proteins. 5FdUR-biased induction of several angiogenesis- and metastasis-inhibitory factors can also be observed, including the serine protease inhibitors Plasminogen Activator Inhibitor 1 (PAI / *SERPINE1*) and Maspin (*SERPINB5*), as well as the extracellular proteins, thrombospondin 1 (TSP1 / *THBS1*), Adhesion G Protein-Coupled Receptor B1 (BAI-1 / *ADGRB1*), and CD82 antigen (KAI / *CD82*). These changes occur in different combinations in the three cell lines (PAI, KAI, TSP1, and Maspin in HCT116-UGI; PAI, BAI-1, and KAI in HCT116-MMR-UGI; and PAI and Maspin in HCT116). The p21 (*CDKN1A*), a key cell-cycle regulatory protein, shows extremely high expression further induced by both drugs, with a significant bias toward 5FdUR in all three cell lines (cf. Fig 5B and S5 Fig). These differences might suggest increased cytotoxicity of 5FdUR treatment; however, this is not the case based on our previous results regarding cell viability [13]. Notably, 5FdUR also induces MDM2 more strongly in all three cell lines, providing a negative feedback loop that attenuates p53 activity at the protein level.

### RT-qPCR validation of 5FdUR-biased induction of *CDKN1A*, *MDM2*, and *CCNG1*

The above RNA-seq analysis clearly demonstrates that the p53 network was induced more strongly upon 5FdUR treatment than upon RTX treatment (cf. Fig 4, Fig 5A, B, S4, and S5 Fig). To validate these high-throughput results, we measured the expression levels of three genes from the p53 network in the HCT116-UGI cell line using RT-qPCR (Fig 5C). We selected the CDK inhibitor p21 encoded by the *CDKN1A* gene, and two negative regulators of p53: the E3 ubiquitin ligase MDM2 (*MDM2* gene) and cyclin G encoded by the *CCNG1* gene. We also extended the analysis to additional concentrations of RTX and 5FdUR. We found that CDKN1A mRNA level was highly elevated after treatment with 20 µM 5FdUR and, to a lesser extent, after treatment with 0.1 µM RTX (relative normalized expression: 6.77 ± 1.72 and 4.22 ± 0.79, respectively), whereas only slight increases were observed with 20 µM RTX or 0.1 µM 5FdUR (1.97 ± 0.44 and 1.83 ± 0.36, respectively). The expression level of MDM2 mRNA was also strongly increased after treatment with 20 µM 5FdUR (3.79 ± 0.48) and moderately increased after treatment with 0.1 µM 5FdUR (2.31 ± 1.33) or either dose of RTX (1.63 ± 0.06 for the lower dose and 1.57 ± 0.03 for the higher dose). For CCNG1 mRNA, similarly small changes were observed following RTX treatments (fold change values are: 1.79 ± 0.019 at 0.1 µM RTX; 1.41 ± 0.09 at 20 µM RTX), whereas treatment with 0.1 µM 5FdUR resulted in virtually no change (1.06 ± 0.04). Treatment with 20 µM 5FdUR resulted in a modest increase in CCNG1 mRNA levels (2.03 ± 0.47). These results are in good agreement with the RNA-seq analysis, further supporting our findings. Moreover, they suggest that the RNA-seq experiments were performed at drug concentrations that produced pronounced transcriptional responses.

### The protein level of p53 is increased in 20 μM 5FdUR-treated cells

As many p53 target genes were elevated while p53 mRNA expression remained unchanged (cf. Fig 5A), we examined p53 protein levels after TS-inhibitory drug treatments by Western blotting (Fig 5D and for biological replicate, S6 Fig). Indeed, in both the nuclear soluble and insoluble fractions of 20 µM 5FdUR-treated cells, a strong increase in the p53 signal was detected. In contrast, following treatment with 0.1 or 20 µM RTX or 0.1 µM 5FdUR, p53 showed only a weak induction compared with the NT samples, which was detected only in the insoluble fraction (Fig 5D and S6 Fig). These results suggest the involvement of additional regulatory mechanisms, either at the translational level or through p53 protein stabilization, that are more pronounced in the high-dose 5FdUR sample than in the low-dose 5FdUR or RTX treatments. This occurs despite the 20 µM 5FdUR-biased induction of MDM2 transcript (cf. Fig 5A-C).

We also examined the DNA damage marker γH2AX, which showed a similarly strong signal increase in the insoluble (chromatin-bound) fraction for all drug treatments, indicating a comparable intensity of DDR. However, in our case, this is not accompanied by similar induction of the p53 protein or its related pathways.

The TS protein could only be detected in the cytoplasmic fractions. Despite our RNA-seq results and previous reports [55], the TS protein level appeared similar across all samples. After 5FdUR treatment, a band duplication was observed, corresponding to the stable ternary complex of TS, 5FdUMP, and the folate co-substrate [14]. The abundance of this covalent inhibitory complex correlated well with the applied drug concentrations: the lower concentration led to less inhibition.

In addition, we examined p21, encoded by the validated drug-induced *CDKN1A* gene. Interestingly, although the p21 mRNA level was strongly elevated after drug treatments, considerably less p21 was present in the cytoplasmic fraction following TS inhibitory treatment than in the NT and DMSO control samples (Fig 5D and S6 Fig). In the nuclear soluble fraction, the p21 protein level was similar in NT and 20 µM 5FdUR-treated cells, whereas it was decreased after treatment with low-dose 5FdUR or any concentration of RTX (Fig 5D and S6 Fig). This discrepancy between mRNA and protein levels suggests the presence of additional post-transcriptional regulatory mechanisms affecting p21 protein abundance.

### Differentially expressed noncoding RNAs in response to RTX or 5FdUR treatments

To address possible drug-specific differences in post-transcriptional regulation, we analyzed the noncoding transcriptome, with particular focus on p53-related RNAs.

### lncRNAs show preferential downregulation in BER-impaired cells

For protein-coding genes, upregulation was generally more common than downregulation (cf. S1 Fig). In contrast, noncoding RNAs (lncRNAs, sRNAs, snoRNAs, and miRNAs) display more diverse patterns (S7 and S8 Figs). In cell lines with impaired BER (HCT116-UGI and HCT116-MMR-UGI), a clear bias toward downregulation of lncRNAs was observed upon treatment with either 0.1 μM RTX or 20 μM 5FdUR (S7A and S7B Fig). In contrast, in the BER-competent HCT116 cell line, treatment with these TS inhibitors resulted in roughly similar numbers of upregulated and downregulated lncRNA genes (S7C Fig).

Among the numerous downregulated lncRNAs, only 20 were commonly downregulated in all six conditions (black dots in S7 Fig), with a further 5 shared among the three RTX-treated samples (dark blue dots in S7 Fig), and 19 among the 5FdUR-treated samples (dark green dots in S7 Fig). Many of the downregulated lncRNAs are expressed at relatively low levels, which may limit their functional relevance. Applying more stringent filtering criteria (expression levels in NT cells ≥ 10.000 FPKM and log_2_(Fold Change) values < −2) reduced the list to a few prominent hits (labeled on volcano plots in S7B and S7C Fig). In HCT116-UGI cells, where both U-DNA BER and MMR are impaired, RTX and 5FdUR treatments resulted in pronounced downregulation of 29 and 21 such lncRNAs, respectively (functionally characterized genes are labeled in S7A Fig).

Among the few upregulated lncRNA genes, only GAS6-DT (also known as GAS6-AS2) was found to be upregulated in all six conditions. LINC02846, AC093866.1, AC015912.3, and AL035661.1 were induced across the three cell lines following 5FdUR treatment (S7 Fig). AL158206.1 showed 5FdUR-selective induction in all three cell lines, although not always with significant drug bias. Only AP002761.4 displayed significant 5FdUR-biased induction across all cell lines (S8 Fig); however, this did not coincide with significant upregulation in the 5FdUR-treated HCT116-UGI cells (larger green dot on each volcano plot in S7 Fig). No significant RTX-biased lncRNAs common to all three cell lines were identified (S8A Fig).

We also examined whether highly abundant and functionally characterized lncRNAs in colon cancer were affected by these treatments. MALAT1 did not show significant changes, whereas NEAT1 expression depended on the cellular DNA repair capacity. In repair-incompetent HCT116-UGI cells, NEAT1 was slightly but significantly downregulated, whereas in HCT116 cells with active U-DNA repair, it was rather upregulated following both treatments (S7 Fig).

### Drug-induced changes in snoRNAs and snRNAs

Although short RNA sequencing data were less reproducible across replicates, a clear trend was observed: miRNAs were predominantly downregulated, whereas snoRNAs were generally upregulated, particularly in UGI-expressing cells (S9 Fig). Upregulated sn/snoRNAs, which are mostly involved in rRNA and mRNA processing, varied substantially among cell lines. The only commonly upregulated sn/snoRNA in all six conditions was SCARNA12, a Small Cajal body-specific RNA.

In HCT116-UGI cells, SNORD49A and SNORD104 were upregulated upon both drug treatments (labeled in S9A Fig). In MMR-proficient cells, numerous C/D-box class snoRNAs (SNORDs) were upregulated following both treatments, although none overlapped with those identified in MMR-deficient cells (S9A and S9B Fig). Seven abundant upregulated SNORDS were common to both drug-treated samples; only SNORD36A and SNORD13 were 5FdUR-specific, whereas six were selectively upregulated in response to RTX treatment. In addition, one abundant H/ACA class snoRNA, SNORA21, and one snRNA, RNU7−1, were also markedly upregulated after both drug treatments in these cells.

Drug responses differed markedly in HCT116 cells with efficient U-DNA repair: the only commonly upregulated abundant snoRNA was SNORD25 (S9C Fig). In 5FdUR-treated HCT116 cells, numerous abundant miRNAs and snoRNAs were upregulated, including SNORDs also upregulated in other cell lines (SNORD104, SNORD49A, and SNORD1B) (S9C Fig).

### miRNAs are predominantly downregulated in UGI-expressing cells

In contrast to snoRNAs, miRNAs were mostly downregulated in UGI-expressing cell lines, whereas differential expression in wild-type HCT116 was more balanced (S9 Fig). In HCT116-MMR-UGI cells, miR-139-5p was the only abundant miRNA (> 10k relative mean expression) showing slight upregulation after 5FdUR treatment (similarly to wild type HCT116 cells in response to both treatments, cf. S9C Fig). Other miRNAs were mainly downregulated but not abundant enough to label (S9B Fig). In HCT116-UGI cells, abundant miRNAs were generally downregulated except miR-185-5p in 5FdUR-treated cells (S9A Fig). In HCT116 cells, several abundant miRNAs were also upregulated (S9C Fig), although expression changes were moderate.

### Drug-specific differences in small RNA expression

Direct comparison of the two inhibitors revealed only a few drug-biased mi/sRNA genes (S10 Fig). In HCT116-UGI cells, only SNORA9 showed significant RTX bias, whereas SNORA38, SNORD118, SNORA64, RNU7−1, and SNORD55 showed significant 5FdUR bias, with the latter two being relatively abundant (S10B Fig). No miRNA showed significant drug bias in these cells. In MMR-proficient cells, 5 snoRNAs, an snRNA, and a miRNA displayed RTX-biased expression; SNORA21 and SNORA63 showed relatively high expression levels (S10C Fig), although SNORA21 was upregulated after both drug treatments (cf. S9B Fig).

In HCT116 cells, the two TS-inhibitory drugs triggered markedly different responses, with far more DE mi/sRNA genes detected in 5FdUR-treated cells than in RTX-treated cells, indicating a strong 5FdUR-biased effect. Among the drug-specific candidates, the RTX-biased miR-32-5p and miR-23a-3p, and the 5FdUR-biased miR-151a-3p and SNORD43, were abundant (>10k mean expression) (S10D Fig). These differences appear to arise mainly from selective down- or upregulation following 5FdUR treatment (cf. S9C Fig). Only two biased DE mi/sRNAs (SNORA38 and SNORA64) were shared among cell lines, and both had low expression levels (<1k) (S10 Fig).

Overall, cell-type–specific differences in miRNA and sn/snoRNA expression were more pronounced than drug-specific effects. Therefore, the observed drug-specific differences in mRNA expression, as well as previously reported differences in cellular responses, mutational rates, and cell survival, likely arise from regulatory mechanisms acting at the transcriptional, translational, or post-translational levels.

### TS RNA-binding ability is influenced by drug treatments

**Fig 6:**
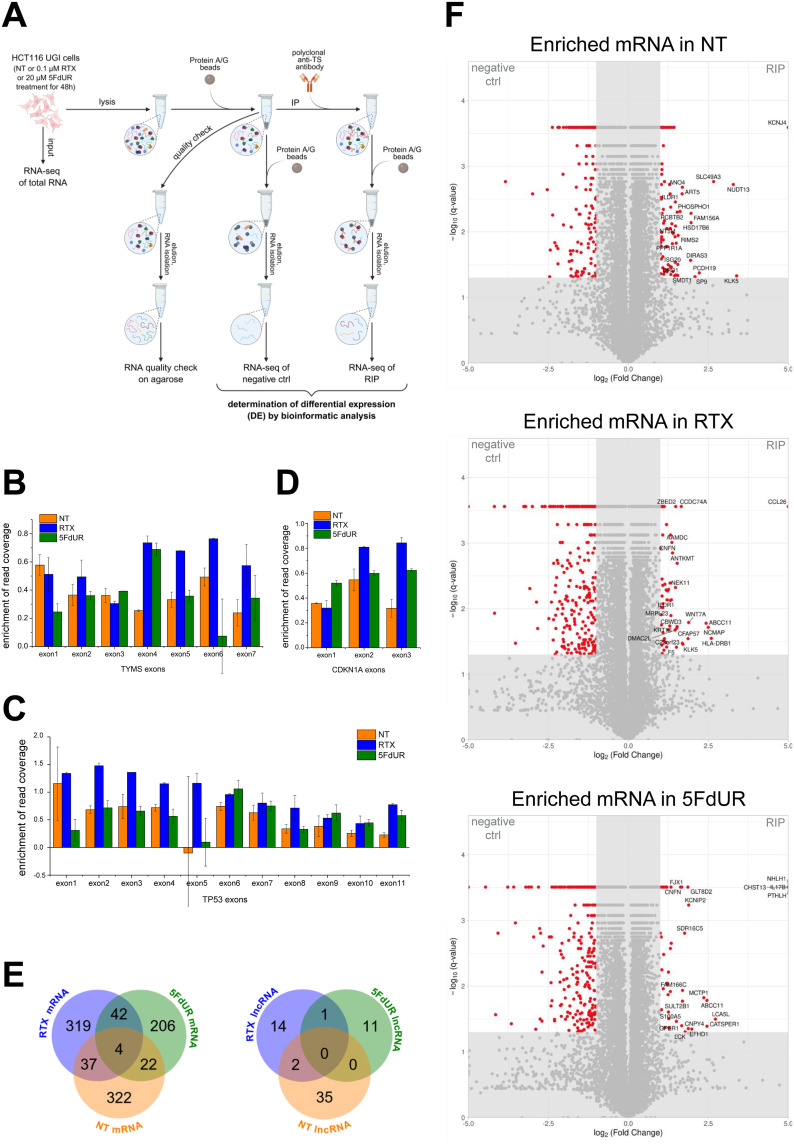
Several RNAs are bound to TS, and drug treatments alter TS-RNA interactions. **A:** Workflow of TS-bound RNA co-immunoprecipitation (TS-RIP) followed by next-generation sequencing. The scheme was created in BioRender (Vértessy, **B.** (2026) https://BioRender.com/x6d0dac). **B-D:** Exon-level TS-RIP enrichment profiles for TYMS, encoding TS **(B)**, TP53 **(C)**, and CDKN1A **(D)**. Average read coverage values were calculated separately for exons, and TS-RIP signals were compared with the corresponding negative control (RIPctr) in non-treated (NT), 0.1 μM RTX-treated, and 20 μM 5FdUR-treated samples. Data are shown as bar graphs; error bars represent standard deviation calculated from pseudoreplicate data. **E:** Venn diagrams showing TS-bound mRNA (left) or lncRNA (right) detected in NT, 0.1 μM RTX-treated, or 20 μM 5FdUR-treated samples. Significant enrichment was defined as log_2_FC>0.585, q-value<0.05, and the mean FPKM > 500. **F:** Volcano plots comparing TS-RIP and the corresponding RIPctr samples for NT, 0.1 μM RTX, and 20 μM 5FdUR treatments. The most enriched genes are labeled based on log_2_FC and q-value.

As TS was described as a moonlighting RNA-binding protein with translation-inhibiting ability [21,22], we assumed that drug treatments could influence these functions, potentially explaining the discrepancies observed between the mRNA and protein levels of p53 and p21. To test this, we performed RNA co-immunoprecipitation using an anti-TS antibody (TS-RIP; scheme in Fig 6A) followed by sequencing of TS-RIP samples and the corresponding control IP samples (RIPctr, pull-down without specific antibody).

Average enrichment values were calculated with Cuffdiff (Table 2), and exon-level enrichment profiles were also examined (Fig 6B-D). Both the TP53 / p53 and TYMS / TS mRNAs could be pulled down; however, their enrichment relative to RIPctr was modest (Table 2). Interestingly, drug treatments increased these enrichments, except for the TYMS mRNA in the 5FdUR-treated sample.

**Table 2:** TS-RIP enrichment data for the selected genes in the three samples: NT, RTX, and 5FdUR. Rows with q < 0.05 are bolded. Values were calculated with the Cuffdiff tool as described in Supplementary Methods.

sample	RNA	FPKM in RIPctr	FPKM in TS-RIP	log_2_ (fold change)	q-value
**NT**	**MYC**	**562836**	**587207**	**0.006**	**0.01852**
**RTX**	**MYC**	**208954**	**222702**	**0.092**	**0.04769**
5FdUR	MYC	226123	226460	0.002	0.96167
NT	TP53	94520	94081	−0.007	0.93237
**RTX**	**TP53**	**88631**	**115828**	**0.386**	**0.00028**
**5FdUR**	**TP53**	**98913**	**115836**	**0.228**	**0.00031**
NT	TYMS	126651	125622	−0.012	0.85482
**RTX**	**TYMS**	**241156**	**277632**	**0.203**	**0.00028**
5FdUR	TYMS	200383	195808	−0.033	0.54803
**NT**	**CDKN1A**	**159164**	**181984**	**0.193**	**0.00026**
**RTX**	**CDKN1A**	**362117**	**478637**	**0.402**	**0.00028**
**5FdUR**	**CDKN1A**	**1.04966e + 06**	**1.27427e + 06**	**0.280**	**0.00031**

For the TYMS mRNA, the highest enrichment in the NT sample occurred at exons 1 and 6, which decreased after 5FdUR treatment but remained unchanged or increased after RTX treatment (Fig 6B). Interestingly, both drugs increased enrichment at exon 4, and RTX additionally increased TS-RIP enrichment at exons 5–7 relative to the NT sample (Fig 6B). These results suggest a stronger interaction between TYMS mRNA and TS protein after RTX treatment than after 5FdUR treatment, consistent with the overall enrichment values (cf. Table 2). Notably, we did not observe significant induction of TS protein following drug treatments, indicating that the reported translational autoregulation of TYMS mRNA is not markedly perturbed under our experimental conditions.

A similar trend was observed for TP53 mRNA (Fig 6C). Treatment with 0.1 μM RTX increased enrichment across most *TP53* exons except exons 1, 5, and 7, which remained similar to NT. In contrast, 20 μM 5FdUR increased enrichment only at exons 6, 9, and 11, while enrichment at exon 1 decreased compared with NT. These findings suggest a weaker interaction between the TS protein and TP53 mRNA following 5FdUR treatment and a stronger interaction after RTX treatment. This observation is consistent with our RNA-seq and Western blot data, where TP53 mRNA levels remained unchanged upon 5FdUR treatment while p53 protein levels were strongly elevated. These results also confirm that the two TS-inhibitory drugs may differently influence the mRNA-binding ability of thymidylate synthase, implying mechanistic consequences.

For CDKN1A mRNA (p21), not yet identified among TS-binder mRNAs, weak enrichment was detected in the NT sample but increased after drug treatments, particularly after RTX (Table 2). This trend was evident for exons 2 and 3, whereas exon 1 showed increased enrichment only after 5FdUR treatment (Fig 6D). The stronger interaction in the RTX-treated sample may contribute to the reduced p21 protein levels observed on Western blot (cf. Fig 5D).

### Thymidylate synthase binds multiple RNAs

Beyond these known targets, in the TS-RIP experiment, we detected numerous additional mRNAs and a few lncRNAs bound to TS, not reported before (Fig 6E and F). Drug treatments affected the composition of these RNA sets differently. While only 42 mRNAs were common to both drug-treated samples but absent in the NT sample, 319 and 206 mRNAs were specific to RTX- and 5FdUR-treated samples, respectively, whereas 322 were NT-specific. 37 mRNAs were shared between NT and RTX samples, 22 between NT and 5FdUR samples, and only four were present in all conditions.

We performed GSEA on TS-associated mRNAs, which revealed enrichment of membrane organelle-related functional terms across all conditions (Table 3). In the NT sample, the most enriched categories included “intrinsic and integral component of membrane”, “bounding membrane component”, and the “organelle membrane”. After RTX treatment, additional Gene Ontology (GO) terms such as “mitochondrial membrane”, “striated muscle thin filament”, and “cytoplasm” appeared, whereas 5FdUR treatment resulted in enrichment of “endoplasmic reticulum membrane”. No significant enrichment was detected among the TS-associated mRNAs shared between NT and either drug treatment, while only the Human T-cell leukemia virus 1 infection was enriched among mRNAs common to both drug-treated samples.

**Table 3:** GSEA results and STRING network statistics of enriched (putatively TS-associated) mRNAs identified in the NT sample and after treatment with 0.1 μM RTX or 20 μM 5FdUR.

	Enriched mRNA in NT RIP	Enriched mRNA in RTX RIP	Enriched mRNA 5FdUR RIP	Enriched common mRNA in NT and RTX RIP	Enriched common mRNA in NT and 5FdUR RIP	Enriched common mRNA in RTX and 5FdUR RIP
Number of nodes	385	400	273	41	26	45
PPI enrichment p-value	0.00301	0.00921	0.0206	0.582	0.0306	0.896
Permanent link	https://version-12-0.string-db.org/cgi/network?networkId=bgRbHU8aErHR	https://version-12-0.string-db.org/cgi/network?networkId=bzlkjbXvXjeo	https://version-12-0.string-db.org/cgi/network?networkId=bsBBGWDf1OOW	https://version-12-0.string-db.org/cgi/network?networkId=bzxij0OF3pyS	https://version-12-0.string-db.org/cgi/network?networkId=bNc00726d5Kb	https://version-12-0.string-db.org/cgi/network?networkId=bUbIJoy1VcA1
Enriched terms (GO or KEGG)	Intrinsic component of membrane GO:0031224, Integral component of membrane GO:0016021, Bounding membrane of organelle GO:0098588, Organelle membrane GO:0031090	Mitochondrial membrane GO:0031966, Striated muscle thin filament GO:0005865, Cytosol GO:0005829, Organelle membrane GO:0031090	Endoplasmic reticulum membrane GO:0005789, Organelle membrane GO:0031090	No enrichment	No enrichment	Human T-cell leukemia virus 1 infection KEGG pathway hsa05166

Compared with mRNAs, substantially fewer lncRNAs were detected in TS-RIP samples (Fig 6E). Moreover, the number of TS-bound lncRNAs decreased in drug-treated cells, likely reflecting the overall downregulation of lncRNAs observed after TS-inhibitory treatments. Among these TS-bound lncRNAs, AC080112.2 was common to both treatments, AC027031.2 and AL109627.1 were shared between NT and RTX samples, and none were shared between NT and 5FdUR samples. AC027031.2 has been proposed to participate in ferroptosis [56], whereas AL109627.1 and AC080112.2 remain uncharacterized. The binding of TS to lncRNAs, as translation inhibition is irrelevant, might rather modulate their stability.

Overall, similar numbers of TS-associated mRNAs were detected in NT and RTX-treated samples, whereas fewer TS-bound RNAs (mRNA and lncRNA) were identified after 5FdUR treatment (Fig 6E). These findings suggest that the formation of the covalent ternary complex among TS, 5FdUR, and the folate cofactor disrupts TS-RNA interactions more strongly than the binding of the antifolate RTX, which, in some cases, might even enhance TS-RNA association.

## Discussion

Thymidylate synthase inhibitory drugs are widely used in chemotherapeutic treatments; however, their exact mechanism of action is not yet fully understood. Here, we provide deeper insight into the cellular response at the RNA level initiated by treatment with the antifolate RTX or the base-analogue 5FdUR. We also present evidence that, although these drugs target the same enzyme, they induce distinct cellular responses that might also depend on cellular repair status. We hypothesized that these differences might be partially due to TS’s RNA-binding and RNA-regulatory roles, which might be differentially affected by drug binding. Accordingly, we demonstrated that TS protein interactions with its own mRNA and with p53 and p21 mRNAs are altered differently after treatments with the two inhibitors. Additionally, we identified previously undescribed RNA-interacting partners of the TS protein that differ in response to inhibition by 5FdUR or RTX.

### Both drugs induce DDR and p53 pathways regardless of the cellular DNA-repair capacity

Comparison of the gene expression profiles of the three cell lines (HCT116, HCT116-UGI, and HCT116-MMR-UGI) treated and untreated revealed cell-line- and drug-dependent changes (cf. Fig 1 and Fig 2). Still, a group of DE genes is shared among the six conditions (cf. Fig 2). The commonly upregulated mRNA genes form a coherent functional network, in which the terms “DNA damage response”, “p53 signaling network”, and “p53-regulated cell cycle” were enriched (cf. S2 Fig). The p21 protein encoded by the *CDKN1A* gene, the most abundant and upregulated member of this network, is a major regulator of cyclin D / CDK4 complexes and might contribute to p53-mediated inhibition of cellular proliferation in response to DNA damage [57]. The Growth Differentiation Factor 15, encoded by the *GDF15* gene, is a stress hormone or cytokine recognized by transforming growth factor receptor beta (TGF-beta) [58]. The Proliferating Cell Nuclear Antigen (PCNA) is an essential factor for efficient DNA synthesis and a key regulator of the polymerase switch in translesion synthesis mediated by its ubiquitination [59,60] or ISGylation [61] in DDR. ISGylation means conjugation of the ubiquitin-like ISG15 [62,63], which is also upregulated in this network (S2A Fig). The 14-3-3 sigma protein encoded by the *SFN* gene serves as a scaffold in many signaling pathways, being a p53-regulated inhibitor of G2/M transition [64], and may also activate p53 by regulating MDM2 autoubiquitination and degradation [65]. The network of these commonly upregulated genes aligns with our previous results, demonstrating drug-induced S-phase arrest and DNA damage [12,13].

### Transcriptomic changes largely depend on the cellular backgrounds

In addition to these common aspects of cellular responses, cell-line-dependent differences were also obvious (cf. Fig 2). These cell lines are isogenic and differ only in their DNA-repair capacities: HCT116 and HCT116-UGI cells are MMR-deficient due to impaired *Mlh1* expression, while this was restored in HCT116-UGI-MMR cells by introduction of a healthy chromosome 3 [66]; the stably expressed UGI transgene efficiently inhibits UNG-initiated U-DNA-repair, as has been demonstrated in HCT116-UGI and HCT116-UGI-MMR cell lines [12]. Notably, the MMR-proficient transcriptomic profiles diverged from MMR-deficient ones even without drug treatments (cf. Fig 1A). The two drugs induce somewhat different changes, whereas the cellular background has a stronger effect (cf. Fig 1A). In some cases, drug treatments result in opposite changes in the gene expression depending on cellular backgrounds, as reflected in the heatmaps of DE mRNAs and lncRNAs (cf. Fig 1B and 1C). Accordingly, the cell-line-specific groups of DE mRNAs are also emerging on the UpSet plots except for the MMR-proficient case (cf. Fig 2). The exception of MMR-proficient cells might be due to fewer DE mRNAs after RTX treatment (cf. S1B Fig) and the larger drug-specific differences (cf. Fig 3). This increased drug-specific divergence can be well explained by the previously detected 5FdUR-induced cytosine deamination, which might trigger MMR activity and coupled DNA-synthesis in these MMR-proficient cells [13]. In the aspect of intense repair-coupled DNA synthesis (either MMR or UNG-initiated BER), the 5FdUR-treated HCT116-MMR-UGI and the drug-treated HCT116 cells are similar. This is also reflected in the large number of common DE mRNAs in these three samples (cf. Fig 2).

### Drug-specific differences are observed in each cell line

In addition to the dominant cell-line-specific features of cellular responses, drug-specific differences were identified in each cell line separately (cf. Fig 3 and S1, S3, S4, S7, S8, and S9 Figs).

### Changes in expression levels of protein-coding genes

In the case of mRNAs, the relative weight of downregulation was usually lower than upregulation, as reflected in the numbers of downregulated genes (cf. Venn diagrams in S1 Fig) and the average amplitudes of changes (cf. volcano plots in S1 Fig). In the group of RTX-specific upregulated protein-coding genes, DNA-synthesis-and cell-cycle-related processes were enriched, whereas in the 5FdUR-specific group, DNA repair and additional signaling pathways emerged (c.f. S3C and S3D Fig). Notably, the three p53-related functional terms that were enriched among the commonly upregulated genes (cf. S2 Fig) were also enriched among drug-specific upregulated genes in the cases of 5FdUR-treated HCT116-UGI and HCT116-MMR-UGI cells (cf. S3D Fig), as well as RTX-treated HCT116-MMR-UGI cells (cf. S3C Fig). However, in the latter case, which is associated with fewer upregulated mRNA genes (cf. Venn diagrams in S1 Fig), the enriched terms “TP53 Regulates Transcription of Cell Cycle Genes” and “Cell Cycle” are somewhat redundant (cf. S3C Fig). Notably, the confidence of the enrichment of “p53 transcriptional gene network” in 5FdUR-specific upregulated genes is outstanding (cf. S3D Fig), which includes genes regulating apoptosis and cell survival (cf. Source Data File 1).

Among the drug-induced downregulated protein-coding genes, DNA synthesis and transcription-related processes were enriched. Interestingly, no functional enrichment was found in either RTX- or 5FdUR-specific downregulation.

In a direct comparison of the two drug treatments within each cell line, genes with RTX- or 5FdUR-biased expression were identified; however, this bias does not necessarily reflect significant up- or downregulation relative to the corresponding NT samples (cf. Fig 3). In UNG-inhibited cells, 5FdUR-biased expression predominated, whereas in HCT116 cells, where UNG-initiated U-DNA repair is active, drug-biased differences were more balanced. Notably, 40 genes exhibited consistent 5FdUR-biased expression across all three cell lines, compared with only 8 genes showing common RTX bias (cf. Fig 3A), suggesting that 5FdUR induces more pronounced transcriptional changes. Moreover, these 40 genes form a relatively interconnected network with enriched functional terms, including the two previously identified p53-related terms (cf. Fig 4).

### Changes in lncRNA gene expression

In HCT116-UGI and HCT116-MMR-UGI cells, most lncRNAs were downregulated following drug treatment compared to NT controls, whereas this trend was not observed in wild-type HCT116, where U-DNA repair remains active. Notably, this downregulation of lncRNAs contrasts with the tendency for upregulation of protein-coding genes. This discrepancy might be related to transcriptome dynamics, including shorter average half-life of lncRNAs compared to mRNAs [67], as well as a general reduction in transcriptional activity on uracil-substituted genomes [68]. Among these downregulated lncRNA genes, 20 were common across all six conditions, with an additional five and 19 shared among RTX- or 5FdUR-treated samples, respectively (cf. dark colored dots in S7 Fig); however, most of these showed low baseline expression in NT samples. The HCT116-UGI cell line, in which both U-DNA BER and MMR are impaired, exhibited the most pronounced lncRNA downregulation (cf. S7A Fig), with 29 and 21 abundantly expressed lncRNAs reduced following RTX and 5FdUR treatment, respectively.

We also examined two well-characterized colon-cancer-associated lncRNAs, MALAT1 and NEAT1. While MALAT1 expression remained unchanged, NEAT1 was downregulated in DNA-repair-deficient HCT116-UGI cells (cf. S7A Fig), but rather upregulated in wild-type HCT116 cells, where U-DNA repair is active (S7C Fig). In MMR-proficient cells, NEAT1 expression showed a slight bias toward 5FdUR treatment (S7B and S8 Fig), consistent with the assumption of extensive repair synthesis induced by U:G mismatches under these conditions [13]. As a structural component of nuclear paraspeckles, NEAT1 plays a key role in sequestration of transcription and splicing factors, as well as specific, usually hyper-edited RNAs, thereby contributing to the regulation of transcription and RNA metabolism [69,70]. Although NEAT1 is a known p53 target gene [71,72], its downregulation in treated HCT116-UGI cells may reflect the global decrease of lncRNA transcription. NEAT1 downregulation may also be associated with the increased γH2AX signal [73], which was observed in our system (cf. Fig 5D).

Against the background of widespread lncRNA downregulation, the relatively few upregulated lncRNAs may play a disproportionate role in shaping the cellular response. Notably, GAS6-DT (GAS6-AS2) and LINC02846 were consistently upregulated across all cell lines following both RTX and 5FdUR treatment. LINC02846 remains functionally uncharacterized, but has been identified as a direct target of p53 [74]. GAS6-DT has been implicated in cancer progression, including resistance and metastasis, through activation of the AXL/AKT/MEK pathway, resulting in repression of apoptosis [75,76] as well as via interactions with cell-cycle-related miRNAs that promote proliferation [77–80]. Consistently, elevated GAS6-DT expression has been associated with worse prognosis in multiple cancers [75,77,78]. Notably, some of the upregulated lncRNAs, which were found in conditions with active DNA repair, are also known targets of p53 [74] (cf. red dots in S9 Fig). Their distribution on the volcano plots further strengthens the view that p53 might have a stronger effect in response to 5FdUR than in RTX, especially in UNG-inhibited cells.

Two additional lncRNAs, AC015912.3 and AC093866.1, were upregulated in most conditions, except 5FdUR-treated and RTX-treated HCT116-UGI cells, respectively. Although their functions are not well defined, AC015912.3 has been linked to cuproptosis [81], while AC093866.1 has been proposed as a prognostic biomarker in gastric cancer [82].

Interestingly, while none of the few RTX-biased lncRNAs were shared across all three cell lines, AP002761.4 exhibited consistent 5FdUR-biased expression (S8 Fig). High AP002761.4 expression level was proposed to be either a positive [83] or a negative [84] prognostic marker, suggesting a cancer-type-dependent role. Furthermore, AL158206.1 (together with AC093866.1) was detected as 5FdUR-biased in the two UGI-expressing cell lines (cf. S8 Fig). AL158206.1 was reported to be up-regulated 4 hours post high-dose ionizing irradiation of primary skin fibroblasts, where CDKN1A, MDM2, TIGAR, BTG2, and HSPA4L were identified as the top five most co-expressing mRNAs [85]. In addition, GSEA performed on all co-expressing RNAs revealed “signal transduction by p53 class mediator” as the most enriched functional category [85]. This suggests a similar cellular response after treatment with TS-inhibitory drugs, particularly with 5FdUR, as most of these mRNAs were also upregulated in our study. It is further supported by the high γH2AX signal in our cells following treatment with RTX or 5FdUR, which is usually a sign for DNA double-strand breaks, typically caused by ionizing radiation.

### miRNA and sn/snoRNA expression changes are primarily cell line–dependent rather than drug-specific

Both the numbers and the distribution of DE mi/sRNAs exhibited strong dependency on cell line and also on the applied drug. In HCT116-UGI cells, comparable upregulation and downregulation were observed following the treatments, although the distribution of miRNAs and sn/snoRNAs exhibited a strong bias (cf. S9A Fig). In HCT116-MMR-UGI cells, upregulation of sn/snoRNAs was dominant after both treatments (cf. S9B Fig). In contrast, in HCT116 cells, the two drug treatments induced markedly different responses: RTX resulted in substantially fewer DE mi/sRNA genes than 5FdUR. Moreover, several miRNAs were induced in HCT116 cells, a pattern not observed in UGI-expressing backgrounds. These pronounced discrepancies limit direct comparisons across cell lines and question the identification of consistent drug-specific signatures at the small RNA level.

Upregulation of several snoRNAs and snRNAs may reflect nucleolar stress, which potentially affects many cellular processes, including rRNA and mRNA processing, cell cycle arrest, and apoptosis [86,87]. The only snoRNA upregulated in all six conditions was SCARNA12, a Small Cajal body-specific RNA, involved in pseudouridylation of U5 snRNA splicing factor and previously identified as an oncogenic biomarker in colorectal cancers [88].

Most upregulated snoRNAs belonged to the C/D-box class (SNORD), which mediates 2′-O-methylation primarily of rRNA, but some of them have mRNA or miRNA substrates [89], and often the same SNORD can be present in methylating and non-methylating ribonucleoprotein complexes [90]. As upregulated SNORDs varied substantially among cell lines (cf. no common DE snoRNAs between HCT116-UGI and HCT116-MMR-UGI cell lines in S9 Fig), true drug-specific differences can hardly be identified (cf. S10 Fig). Only the 5FdUR-biased SNORA38 and SNORA64 are shared between MMR-deficient cell lines (cf. S10A and S10C Fig), from which SNORA38 has been reported as a marker for tumor progression and metastasis in breast cancer [91]. These snoRNAs belong to another main class containing H/ACA box, which is typically involved in pseudouridylation of several RNA targets [92]. In our study, only one abundant H/ACA class snoRNA, SNORA21, was differentially expressed, and only in MMR-proficient cells (S9B Fig); SNORA21 has also been reported as an oncogenic biomarker in colorectal cancers [93,94]

RNU7−1, which was strongly upregulated in MMR-proficient cells and showed 5FdUR-biased expression in HCT116-UGI cells, is an snRNA component of U7 snRNP required for non-canonical processing of replication-dependent histone pre-mRNA [95].

Among the few abundant upregulated miRNAs, miR-139-5p appears in the three conditions where DNA-repair is active. miR-139-5p is a well-characterized tumor-suppressing miRNA [96] that is a known target of the p53 transcription activator [97] and potentially modifies the p53-dependent cellular response [98].

In addition, in HCT116 cells, miR-192-5p, miR22-3p, and miR-34a-5p are abundant and upregulated after both treatments (cf. S9 Fig), although the abundant lncRNA MALAT1 is a known sponge for all of them [99]. miR192-5p [100–102] and miR-34a-5p [103] have been identified as potential biomarkers suppressing tumor progression in colorectal cancers.

In DNA-repair-deficient cells, only 5FdUR treatment induces expression of a single abundant miRNA, miR-185-5p, which is known to target CDK4 and CDK6 in colon cancer cells [104]. Moreover, NEAT1 has been reported as a sponge for miR-185-5p [105]; in these cells, where NEAT1 is downregulated (cf. S7A Fig), the effect of this upregulated miRNA might be more pronounced in the regulation of migration and epithelial-mesenchymal transition (EMT) [106].

Above the cell-line-specific upregulation of several tumor suppressor miRNAs, the massive downregulation in UGI-expressing cells deficient in U-DNA repair might strongly contribute to the overall cellular response (cf. S9A and S9B Fig). Interestingly, abundant downregulated miRNAs were detected only in HCT116-UGI cells (6 and 10 after 5FdUR and RTX treatments, respectively), as well as in 5FdUR-treated HCT116 cells, where the drug effect was robust and not biased towards downregulation of miRNAs (cf. S9C Fig).

Although a stronger influence of p53-related pathways by 5FdUR treatment was demonstrated at the mRNA level, corresponding drug-specific changes were not consistently observed in the mi/snRNA dataset across all three cell lines. This suggests that the differential activation of p53-associated responses by 5FdUR versus RTX is primarily regulated at the transcriptional or translational/post-translational level, rather than through a shared small RNA-mediated decay.

### 5FdUR-biased induction of p53-related pathways relies on translational control

The observed 5FdUR-biased activation of the p53 pathway was confirmed by RT-qPCR analysis of p53-regulated genes (*CDKN1A, MDM2, and CCNG1*) in HCT116-UGI cells (cf. Fig 5C). As TP53 mRNA levels did not change significantly upon treatments (cf. Fig 5A and S4 Fig), we hypothesized that the differential expression of p53 target genes reflects increased p53 protein levels. Indeed, a marked increase in p53 protein level was detected in the nuclear extract specifically after 20 μM 5FdUR treatment (cf. Fig 5D and S6 Fig). Furthermore, in the insoluble fraction, which must contain chromatin including proteins strongly bound to genomic DNA, was also increased following high-dose 5FdUR treatment, whereas comparable levels were detected after RTX and low-dose 5FdUR treatments, all exceeding control levels (cf. Fig 5D and S6 Fig). This is consistent with the common (cf. S2 Fig) and 5FdUR-biased (cf. Fig 4 and 5, and S3 Fig) induction of p53 target genes, as increased p53 level in the insoluble fraction might reflect higher occupancy of p53-binding sites on genomic DNA.

In contrast, the p53 target, *CDKN1A,* exhibited strong and 5FdUR-biased upregulation at the mRNA level. Still, the encoded p21 protein level remained unchanged or was even reduced following TS-inhibitory treatments compared to controls (cf. Fig 5C and 5D). This contradiction may be explained by the post-transcriptional regulation of p21 mRNA mediated by calreticulin, which inhibits p21 mRNA translation [107]. Supporting this, calreticulin expression was elevated in RNA-seq data of UNG-inhibited cell lines after both treatments (S2 Table), consistent with the observed suppression of p21 protein levels. lncRNA SPUD, generated from the *CDKN1A* locus via alternative polyadenylation and known to regulate p21 expression by competing for calreticulin binding [108] showed extremely low expression across all conditions, below the reliable detection limit (cf. bigwig files at GEO under GSE318306); therefore, this competitor cannot contribute to the translational regulation of p21. The expression of the translational activator, CELF1 (CUGBP1) [107] was unchanged, or even downregulated in 5FdUR-treated HCT116-MMR-UGI cells (S2 Table). Together, these results suggest that p21 protein levels are primarily controlled by calreticulin-mediated translational inhibition in this context.

Our results show that the p53 pathway is initiated after both TS-inhibitory treatments; however, high-dose 5FdUR treatment causes a stronger effect. In addition, we could detect further differences between the RTX- and 5FdUR-induced RNAs, which might also contribute to the biased activation of the p53 pathway and, after all, to altered cell survival upon 20 μM 5FdUR treatment [13].

### The RNA-regulatory role of TS is drug-dependent

A potential translational modulating factor might be TS due to its ability to bind RNA (similarly to calreticulin), which might contribute to different cellular responses upon TS-inhibitory drug treatments. Indeed, in TS-bound RNA co-immunoprecipitation and sequencing, we could detect drug-induced differences in the enrichments of read coverage at different exons of TS/*TYMS* and p53/*TP53* mRNAs, two already described binding partners (cf. Fig 6B-C). Similarly, TS-RIP-seq enrichment patterns displayed drug-induced changes on p21/*CDKN1A* exons (cf. Fig 6D), which indicates a potential contribution of TS to the translational regulation of p21 levels. Our results revealed that upon treatment with 5FdUR, p53 mRNA is less bound to the TS protein than in the case of RTX treatment or NT. This result might explain how the p53 protein level is strongly elevated only upon 5FdUR treatment without changes in its mRNA level. In the case of p21 mRNA, we propose a stronger interaction following RTX treatment than in 5FdUR-treated or non-treated cells, further contributing to the posttranscriptional regulation of p21. We also observed that TS, p53, and c-Myc mRNAs (known RNA-interacting partners of TS) were not among our strongest hits (cf. Table 2). However, we detected several new potentially TS-binding RNAs, which, interestingly, were all enriched in functional terms related to membranes in GSEA (cf. Table 3). We could also detect a few lncRNAs among the TS-associated RNAs whose stability might be influenced upon TS binding. At the same time, the interaction with TS might interfere with the post-transcriptional regulatory role of lncRNAs, which requires further investigation.

## Conclusions

We reported differences in cellular response at the RNA level between TS-inhibitory drugs, RTX, and 5FdUR, which are further influenced by the cellular repair status. Despite massive cell-line-specific effects, the 5FdUR-biased induction of p53-related pathways was demonstrated; however, p53 mRNA was not induced. In contrast, an increased p53 protein level was observed selectively after high-dose 5FdUR treatment, which could explain the stronger activation of p53-target genes and might contribute to the diverged cellular response previously characterized in this condition [13]. In addition, the observed discrepancy between the p53 mRNA and protein levels indicates key regulatory mechanisms at the translational or post-translational levels. Furthermore, p21 mRNA as a p53-target was strongly induced, especially after high-dose 5FdUR treatment; in contrast, p21 protein was massively downregulated in drug-treated cells. This phenomenon can also be explained by translational regulation involving calreticulin, a reported inhibitor of p21 translation [107], whose expression was induced after drug treatments. In addition, TS could also contribute to translational regulation via its RNA-binding ability, as previously described [21,109]. In TS-RIP-seq experiments, we demonstrated a wide repertoire of possible TS-binding RNAs, including p53 and p21, whose binding profiles were differentially affected by the two drugs. Although newly identified putative TS-bound RNAs require further experimental confirmation, these results may guide future research addressing the role and impact of TS’s RNA-binding function.

Notably, the observed pronounced DNA-repair-dependent transcriptional differences indicate that these drugs may differentially impact the transcriptomes of other tumor cell types. However, a higher potential of 5FdUR treatment to affect p53-related pathways has been demonstrated. Our findings provide new insights into these drugs’ mechanisms of action, contributing to a better understanding of the cellular responses arising upon treatment with RTX or 5FdUR, with a potential impact on drug resistance mechanisms.

## Materials and methods

### Cell culturing

HCT116, UGI expressing HCT116 (HCT116-UGI), and its MMR proficient version (HCT116-MMR-UGI) cells [12] were all maintained in McCoy’s 5A medium (Thermo Fisher Scientific (Gibco), 16600082) supplemented with 10% FBS (Sigma, F9665-500ML) and 1% PenStrep (Thermo Fisher Scientific (Gibco), 15140122). Mycoplasma contamination was regularly checked. Cells were cultured in 6-well plates, treated with 0.1 µM and 20 µM of RTX or 5FdUR for 48 hours. Because RTX was dissolved in dimethyl sulfoxide (DMSO), it was used as a vehicle control in Western blot analysis.

### RNA isolation

Drug-treated cells were harvested, and the total RNA pool was isolated using TRIzol reagent (ThermoFisher Scientific, 15596018) according to the manufacturer’s protocol. Briefly, cells were suspended in TRIzol reagent, incubated for 5 min at room temperature, and extracted with chloroform, then centrifuged at 12,000 × g at 4 °C for 15 min. RNA-containing aqueous phase was collected, and RNA was precipitated with ice-cold isopropanol, washed with 75% ethanol, dried at 55 °C, and dissolved in RNase-free water. RNA sample quality was checked on a 1% agarose gel, and the concentration was measured using a NanoDrop 2000C (ThermoFisher).

### RNA-seq

Total RNA samples were used to establish stranded RNA sequencing libraries for short RNAs (below 200 nt), and long RNAs (above 200 nt), separately. Small and long RNA libraries were sequenced on a Novaseq6000 platform using Illumina 50 SE (20M reads) and 150 PE (150M reads), respectively. Library preparations, including rRNA depletion and QC, as well as the sequencing, were provided by Novogene Co., Ltd. (China) as a whole transcriptome sequencing service.

### Bioinformatics – RNA-seq data analysis

From long RNA sequencing data, after quality check using fastQC [110], residual rRNA-related reads were removed in silico (script is provided in Supplementary Methods), then polyG, adapter, and low-quality sequences were trimmed using the fastp tool [111]. Clean reads were aligned to the human reference genome GRCh38 [112] using HiSAT2 aligner [113]. Transcriptome reconstruction was performed, and differential expression was analyzed using the Cufflinks package [49,50]. The annotated “*.cuffdiff.gene_exp.tsv.gz” files for each comparison are available at GEO. Significantly up- or downregulated genes were identified using filters for minimal mean expression level (meanFPKM ≥ 1000) and for the change of expression level (abs(log_2_(Fold Change) ≥ 1, q-value<0.05).

Short RNA data were subjected to adapter and quality trimming using trimmomatic [114], then aligned to the GRCh38 reference genome using STAR [115]. Read coverage was quantified on annotated mature miRNAs (hsa.gff3, MiRBase, https://www.mirbase.org/download, [45,46]) and sRNAs, snoRNAs (GENCODE V34, https://www.gencodegenes.org/human/release_34.html, [43,44]) using the FeatureCounts tool [116], and compared using DESeq2 [117,118]. The annotated “*DEsRNA.deseq2.tsv.gz” files for each comparison are available at GEO. Significantly up- and downregulated short RNAs were determined using filters for a minimal expression level (meanCount ≥ 25) and for the change of expression level (abs(log_2_(Fold Change) ≥ 0.585 and FDR < 0.05).

Upset plots [47] and volcano plots were created in R [119]. Gene Set Enrichment Analysis (GSEA) was performed using the STRING database online analysis tool (https://string-db.org [48]), on external databases: Gene Ontology [120], KEGG [121], Reactome [122], WikiPathways [123], and Compartments [124]. Detailed computational pipelines and scripts are provided in Supplementary Methods. These RNA-seq data have been deposited in NCBI’s Gene Expression Omnibus [125] and are accessible through GEO Series accession number GSE318306 (https://www.ncbi.nlm.nih.gov/geo/query/acc.cgi?acc=GSE318306).

### qPCR validation of RNA-seq results

Reverse transcription-coupled quantitative PCR was performed to validate some of the findings of our RNA-seq analysis. Namely, we measured the gene expression levels of CDKN1A/p21, MDM2, and CCNG1/cyclin G, in addition to CNOT4 and PUM1, as reference genes. Specific primers for each target were used based on previous publications [126–129]. Reverse transcription efficiency and primer efficiency were checked for each target. 400 ng RNA was subjected to reverse transcription using the High-Capacity cDNA Reverse Transcription kit (Applied Biosystems, 4368814). Each 10 μl qPCR mix contained 2X MyTaq HS MIX (Bioline, BIO-25046), 20X EvaGreen Dye (Biotium, #31000), 500−500 nM reverse and forward primers, 0.15 μl reverse transcribed mix, and nuclease-free water. Each measurement was performed in technical triplicate and on at least two biological replicates with no-template control for each target in a CFX96 Touch Real-Time PCR Detection System (Biorad). The qPCR annealing temperature was 62 °C for 15 sec, which was followed by a 72 °C 30 sec elongation for 50 cycles. Melting curve analysis was also performed after each PCR reaction.

### RNA co-immunoprecipitation via thymidylate synthase (TS-RIP)

Cells were cultured in 15 cm Petri dishes and treated with 100 nM RTX, 20 µM 5FdUR, or nothing (NT). After the 48-hour treatments and a PBS wash, cells were harvested using a scraper and centrifuged (150 × g, 21 °C), then resuspended in PBS for counting. After a second centrifugation, cells were resuspended in cytoplasmic extraction buffer (1 ml/10^7^ cells, composition: 20 mM TRIS, pH 7.4, 10 mM NaCl, 3 mM MgCl_2_, 0.5 mM dithiotreitol, 0.05% NP40, Protease inhibitor tablet (Roche)) and incubated on ice for 15 min with frequent mixing. Upon centrifugation (3000 × g, 4 °C, 7 min), the supernatant was kept as the cytoplasmic fraction. The pellet was resuspended in nuclear extraction buffer (200 µl / 10^7^ cells, composition: 50 mM TRIS, pH 7.4, 150 mM NaCl, 50 mM NaF, 5 mM EDTA, 1 mM EGTA, 1 mM PMSF, 1% NP40, Protease inhibitor tablet (Roche)) and incubated on ice for 30 min with frequent vortexing. After centrifugation (16000 × g 10 min 4 °C), the supernatant (nuclear extract) was collected, reunited with the cytoplasmic fraction, and supplemented with glycerol (final conc. 6.5%). Then these lysates were added to 55 μL pre-washed magnetic Protein AG beads (Thermo Scientific, Cat. no. 88803) and incubated at 4 °C for 2 h with constant rotation for pre-clearing. Beads were collected and used as a negative control; they were resuspended in the same buffers and kept in the same conditions as the RIP samples. The pre-cleared lysate was subjected to TS RNA co-immunoprecipitation (RIP): first, overnight incubation with anti-TS antibody (3–4.5 ug; Proteintech 15047–1-AP), then 2-hour incubation with washed magnetic protein A/G beads at 4 °C with constant rotation. Then, the beads were collected and washed three times with low-salt buffer (20 mM TRIS, pH 8.0, 150 mM NaCl, 2 mM EDTA, 1% TritonX), and once with the washing buffer (DEPC-treated PBS, 0.05% Tween20). For elution, beads were incubated in 200 µl elution buffer (100 mM TRIS, 10 mM EDTA, 1% SDS, 1 µg/ml Proteinase K) at 60 °C for 30 min with frequent vortexing. 5% of the pre-cleared lysates were saved as input samples and incubated under the same conditions as the RIP samples, including the final Proteinase K treatment. The eluted solution was subjected to RNA isolation (Geneaid, VR050) following the manufacturer’s instructions. RNA concentration was measured with the Qubit RNA BR assay kit (Thermo Fisher Scientific, Q10211) on a Qubit 4.0 (Thermo Fisher Scientific, Waltham, MA, USA). The RNA integrity in the samples was checked on 1% agarose gels compared to the input samples (Fig S11B). The presence of the TS enzyme during the RIP was controlled by Western blotting (Fig S11A). RNA samples (RIP and negative control) were sent for sequencing.

### Sequencing of TS-RIP samples and data analysis

The libraries for Illumina sequencing were prepared using xGen RNA Lib Prep Kit (Integrated DNA Technologies, Iowa, USA). Briefly, 400 ng RNA was rRNA-depleted using RiboCop rRNA Depletion Kit for Human/Mouse/Rat V2 (Lexogen, Vienna, Austria). Thereafter, the RNA was fragmented and reverse-transcribed using random priming, then the cDNA was tailed and adapter-ligated. Finally, the libraries were amplified according to the manufacturer’s instructions. The quality of the libraries was checked on Agilent 4200 TapeSation System using D1000 Screen Tape (Agilent Technologies, Palo Alto, CA, USA), and the quantity was measured on Qubit 3.0 (Thermo Fisher Scientific, Waltham, MA, USA). Illumina sequencing was performed on the NovaSeq X Plus instrument (Illumina, San Diego, CA, USA) with 2 × 151 run configuration. The whole procedure of library preparation and sequencing was performed as a service of the Hungarian Centre for Genomics and Bioinformatics, Szentágothai Research Centre at the University of Pécs, Hungary.

Sequencing data were processed by the same pipeline as the long RNA sequencing data described earlier. TS-RIP samples were compared to the corresponding RIPctr samples. In addition, TS-RIP enrichment was also calculated for exons. For this, all human exons annotated in GenCode V34 were filtered for those that have a detectable average read coverage in the transcriptome sequencing. Read coverage and log_2_(fold change) tracks were calculated using the deeptools package [130]. These RIP-seq data have been deposited in NCBI’s Gene Expression Omnibus [125] and are accessible through GEO Series accession number GSE307531 (https://www.ncbi.nlm.nih.gov/geo/query/acc.cgi?acc=GSE307531).

### Western blot

Fractionation lysis was performed as described in the beginning of TS-RIP section. In the end of the lysis the leftover insoluble fraction was resuspended in PBS (200 µl / 107 cells). All samples were supplemented with Laemmli SDS sample buffer (4X) boiled at 95 °C 7 min and stored at –20 °C. Samples were subjected to SDS-PAGE using a 12% polyacrylamide gel, followed by transfer to PVDF membrane (Millipore; IPVH09120) in 10 mM CAPS 15% EtOH pH 12, 350 mA 3 h. The membrane was blocked using 5% milk powder in PBS-T. Primary antibodies were added in 5% BSA PBS-T (anti-TS-ab: 1:3000 (Proteintech 15047–1-AP), anti-p53-ab: 1:2000 (DO-1 clone, Santa Cruz sc-126), anti-p21-ab: 1:1000 (Cell signaling Technology #2947), anti-actin-ab: 1:2500 (Sigma A1978), anti-γH2AX-ab: 1:750 (Millipore 05–636)) and incubated at 4°C overnight (ON). After several washing steps, HRP-conjugated secondary antibodies were added (1:10,000) and incubated for 2 h at RT. After several washing steps with PBS, visualization of the membrane was performed using Immobilon Western Chemiluminescent HRP Substrate (Millipore) on a ChemiDoc MP Imaging System (Bio-Rad).

## Supporting information

S1 Fig Comparison of upregulated and downregulated protein-coding genes after treatment with 0.1 μM RTX or 20 μM 5FdUR in HCT116-UGI (A), HCT116-MMR-UGI (B), and HCT116 (C) cell lines. Treatment-induced differential expression was calculated from long RNA sequencing data using the Cufflinks package. Numbers of differentially expressed protein-coding RNA genes are shown on Venn diagrams (left). Significantly DE genes (fold change ≥ 2, q-value < 0.05, and mean FPKM > 1000) are shown on Volcano-plots with dark gray on a white background. Those that show drug-biased changes in each of the three cell lines (cf. Fig 3A) are colored with shades of blue (8 RTX-biased) or green (40 5FdUR-biased). Those genes with drug-biased expression that were significantly upregulated in the given condition are also labeled by their gene symbols.
(TIF)

S2 Fig Network of commonly upregulated 121 protein-coding genes. (A) Network data from the STRING database (Table 1) were visualized in CytoScape. The mean FPKM values measured in non-treated samples were mapped to the node size, ranging from the smallest FPKM of 614 (EBI3) to the highest of 200372 (CDKN1A). Enriched functional terms were mapped to the node colors as indicated: p53sig – “p53 signaling pathway” in KEGG Pathways (hsa04115), p53reg – a “p53 transcriptional gene network” of WikiPathways (WP4963), p53CC – “TP53 Regulates Transcription of Cell Cycle Genes” in Reactome Pathways (HSA-6791312), and DDR – “DNA damage response” of WikiPathways (WP707). The border color was mapped to the STRING score for extracellular localization as indicated on the color bar. (B) GSEA results for the 121 commonly upregulated genes. Selected enriched terms are indicated (vertical axis), dot size is mapped to the enrichment strength as indicated, and horizontal positions of dots reflect the reliability of the enrichment as measured by the -log_10_FDR. The enriched terms are from the GO cellular component (GO), WikiPathways (WP), KEGG Pathways (hsa), and Reactome Pathways (HSA). The number of genes associated with each term in the current network is indicated relative to the total number of genes annotated with that term.
(TIF)

S3 Fig Summary of GSEA results. GSEA was performed using the online Analysis tool of the STRING database, with the whole genome as background. Selected enriched terms corresponding to GO molecular function, KEGG Pathways, Reactome Pathways, WikiPathways, and Compartments (vertical axis) and the corresponding -log_10_(FDR) values (horizontal axis) are plotted on the enrichment maps. The enrichment strength is mapped to the size of the dots. Different sets of conditions are colored as indicated. GSEA was performed for gene sets that were either commonly upregulated (A) or downregulated (B) by both drugs, or upregulated only by RTX (C) or 5FdUR (D) treatments in the three cell lines independently (cf. Venn diagrams in S1 Fig). The gene lists and all details are provided in Source Data File 1 (Source-data_Fig4_S2Fig_S3Fig.xlsx). Permanent links for the corresponding STRING networks and network statistics are given in Table 1.
(TIF)

S4 Fig Components of the p53-related network among DE mRNAs. DE mRNAs in HCT116-MMR-UGI (A) and HCT116 (B) cell lines are presented on Volcano-plots: direct comparisons of the two drug treatments (0.1 μM RTX and 20 μM 5FdUR, top plots, the same as in Fig 3C and D), and the two treatments compared to the corresponding NT samples (mid and bottom plots, the same as S1B and S1C Figs). In the top plots, the p53-related genes from the WikiPathways (WP4963), and the KEGG Pathways (hsa04115) are colored either with dark gray (not significantly differentially expressed), or shades of green (5FdUR-biased) or blue (RTX-biased) following the previously applied color code. TP53 (encoding p53) is marked with red. In the bottom plots, components of the same p53-related pathways are colored according to the color code previously applied for the different conditions, and those that displayed drug-biased differential expression are marked in red.
(TIF)

S5 Fig Differential expression projected to the p53 signaling pathway. Using the clusterProfiler, the hsa04115 pathway from the KEGG Pathways was colored according to differential expression data comparing the two drug treatments in (A) HCT116-MMR-UGI and (B) HCT116 cells. The colors of the network nodes are mapped to the log_2_(Fold Change) values according to the color bar from 5FdUR-biased (green) to RTX-biased (red) expression. White boxes represent non-significant drug bias, gray represents multiple genes behind the network nodes.
(TIF)

S6 Fig Western blots on biological replicates for p53, actin, TS, and γ-H2AX. The uncropped images of each blot are provided in the Source Data File 3 (Source-data_Uncropped-Western-blots.pdf).
(TIF)

S7 Fig Comparison of DE lncRNA genes after treatment with 0.1 μM RTX or 20 μM 5FdUR in HCT116-UGI (A), HCT116-MMR-UGI (B), and HCT116 (C) cell lines. Treatment-induced differential expression was calculated together with protein-coding genes (cf. S1 Fig). The numbers of DE lncRNA genes are summarized in Venn diagrams (left). Significant DE lncRNA genes (fold change ≥ 2, q-value < 0.05, and mean FPKM > 1000) are shown on Volcano-plots with dark gray on a white background. Those that were commonly upregulated in the three cell lines in response to either RTX or 5FdUR treatments are colored using the condition-specific color code and labeled by their gene symbol. The only lncRNA gene that shows 5FdUR-biased expression in all three cell lines, AP002761.4 (enlarged green dot), is labeled on each Volcano plot. Commonly downregulated lncRNA genes in all six conditions (black), in the three RTX-treated (dark blue), and in the three 5FdUR-treated (dark green) cell lines are also marked. Pronounced downregulated lncRNA genes (expression level in NT cells ≥ 10.000 FPKM, and log_2_(Fold Change) values < −2) are labeled with their gene symbols. On panel A, pronounced downregulated lncRNA genes without known functions are not labeled. lncRNAs that are known targets of p53 are labeled with smaller red dots.
(TIF)

S8 Fig Drug-biased expression of lncRNAs. A direct comparison of the two drug effects in lncRNA expression data is presented in Venn diagrams (A), and by Volcano plots for HCT116-UGI (B), HCT116-MMR-UGI (C), and HCT116 (D) cell lines. 5FdUR-bised lncRNAs common across cell lines are highlighted with rectangular (all cell lines) or oval (2 cell lines) shapes.
(TIF)

S9 Fig Comparison of DE small RNA genes after treatment with 0.1 μM RTX or 20 μM 5FdUR in HCT116 UGI (A), HCT116 MMR UGI (B), and HCT116 (C) cell lines. Treatment-induced differential expression of short RNAs (including miRNAs and sn/snoRNAs) was calculated using featureCounts and deseq2 as described in Materials and Methods. The numbers of DE miRNA (top numbers) and sRNA (bottom numbers) genes are summarized in Venn diagrams (left). Significant DE mi/sRNA genes (fold change ≥ 1.5, q-value < 0.05, and mean counts > 25) are shown on Volcano-plots colored using the condition-specific color code on a white background. Those genes that were commonly up- or downregulated by both drugs in the given cell line are marked with darker colors, and abundant ones are labeled by their gene names (mean counts above 10k (bigger font size) or between 1k and 10 k (smaller font size)).
(TIF)

S10 Fig Drug-biased expression of mi/sRNAs. A direct comparison of the two drug effects in short RNA expression data is presented in Venn diagrams (A), and by Volcano plots for HCT116-UGI (B), HCT116-MMR-UGI (C), and HCT116 (D) cell lines. Significant DE mi/snoRNA are colored according to the previously applied condition-specific color code and labeled by their gene names. The font size of the labels reflects the abundance of these genes: (mean counts above 10k (bigger font size, only on panel D), between 1k and 10 k (smaller font size), or between 100 and 1k (smallest size, on panels B and C).
(TIF)

S11 Fig Quality check of thymidylate synthase coupled RNA-immunoprecipitation (TS-RIP). (A) Western blotting on TS-RIP fractions. Samples from each step are indicated above the blot. Molecular mass marker: GRS Protein Marker MultiColour (GRISP Research Solutions, Porto, Portugal), “RIP before elution” indicates the antibody-conjugated beads that successfully recruited TS protein from the pre-cleaned lysates (NT, RTX, 5FdUR samples as indicated at the top. “Negative ctrl” samples represent protein A/G agarose incubated with lysates without anti-TS antibody. Position of TS (~35.7 kDa) is marked (triangle on the right). (B) Quality of input RNA analyzed on 1% agarose gel. The input samples were incubated under the same conditions as the TS-RIP and negative control samples. The two main bands correspond to abundant rRNAs, indicating the extent of their preservation during the long immunoprecipitation procedure.
(TIF)

S1 Table Abbreviations. (XLSX)

S2 Table Expression data of genes regulating p21 translation. (XLSX)

S1 File Supplementary methods. (DOCX)
