# Supplementary figures and images for: Thymidylate synthase inhibitory drugs induce p53-dependent pathways differently
Source: PLoS One. 2026 Jul 1;21(7):e0332491. doi: 10.1371/journal.pone.0332491 (PMC13322534; doi:10.1371/journal.pone.0332491)

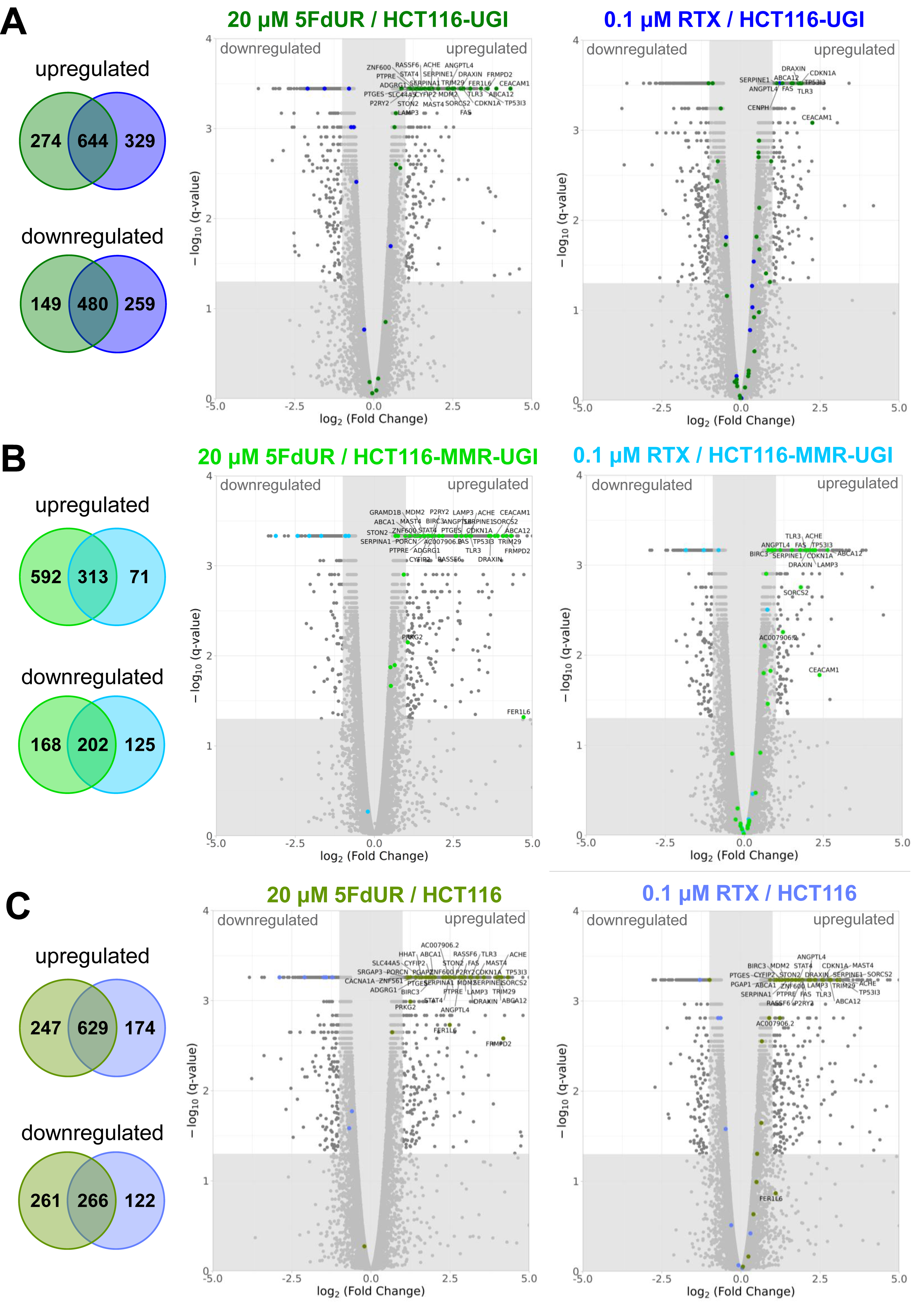

Supplement: S1 Fig — Treatment-induced differential expression was calculated from long RNA sequencing data using the Cufflinks package. Numbers of differentially expressed protein-coding RNA genes are shown on Venn diagrams (left). Significantly DE genes (fold change ≥ 2, q-value < 0.05, and mean FPKM > 1000) are shown on Volcano-plots with dark gray on a white background. Those that show drug-biased changes in each of the three cell lines (cf. Fig 3A) are colored with shades of blue (8 RTX-biased) or green (40 5FdUR-biased). Those genes with drug-biased expression that were significantly upregulated in the given condition are also labeled by their gene symbols. (TIF) [file pone.0332491.s001.tif]

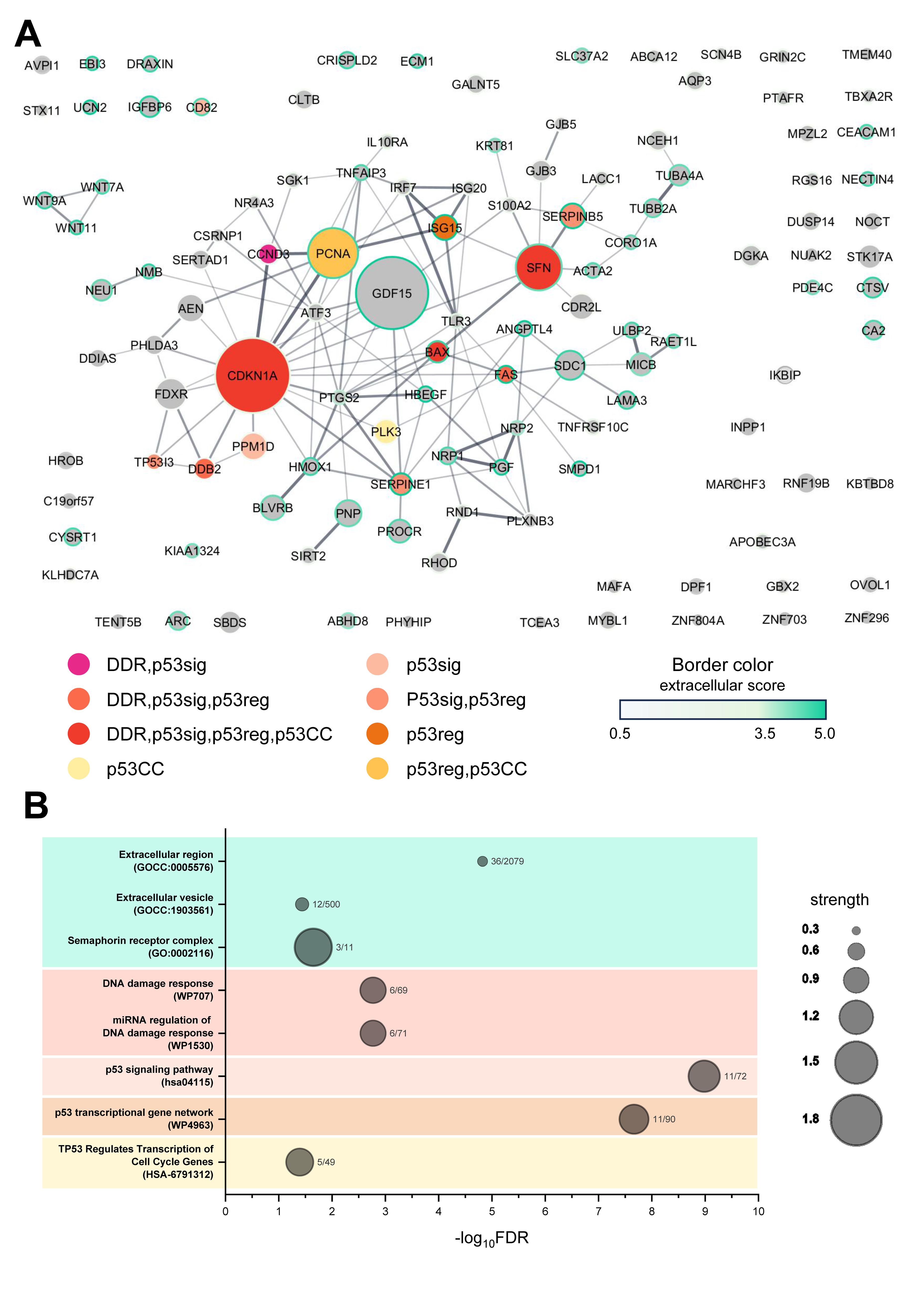

Supplement: S2 Fig — (A) Network data from the STRING database (Table 1) were visualized in CytoScape. The mean FPKM values measured in non-treated samples were mapped to the node size, ranging from the smallest FPKM of 614 (EBI3) to the highest of 200372 (CDKN1A). Enriched functional terms were mapped to the node colors as indicated: p53sig – “p53 signaling pathway” in KEGG Pathways (hsa04115), p53reg – a “p53 transcriptional gene network” of WikiPathways (WP4963), p53CC – “TP53 Regulates Transcription of Cell Cycle Genes” in Reactome Pathways (HSA-6791312), and DDR – “DNA damage response” of WikiPathways (WP707). The border color was mapped to the STRING score for extracellular localization as indicated on the color bar. (B) GSEA results for the 121 commonly upregulated genes. Selected enriched terms are indicated (vertical axis), dot size is mapped to the enrichment strength as indicated, and horizontal positions of dots reflect the reliability of the enrichment as measured by the -log10FDR. The enriched terms are from the GO cellular component (GO), WikiPathways (WP), KEGG Pathways (hsa), and Reactome Pathways (HSA). The number of genes associated with each term in the current network is indicated relative to the total number of genes annotated with that term. (TIF) [file pone.0332491.s002.tif]

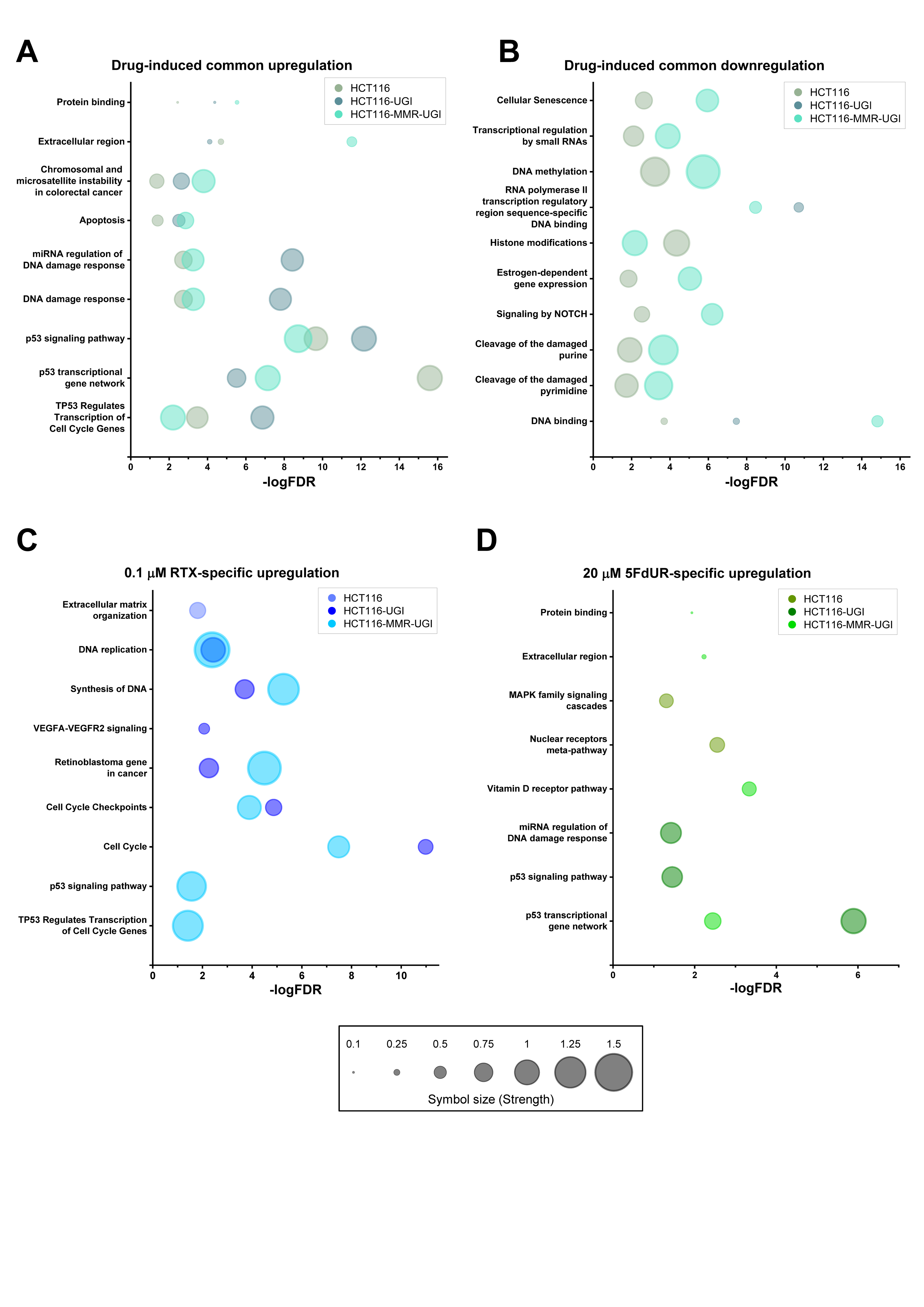

Supplement: S3 Fig — GSEA was performed using the online Analysis tool of the STRING database, with the whole genome as background. Selected enriched terms corresponding to GO molecular function, KEGG Pathways, Reactome Pathways, WikiPathways, and Compartments (vertical axis) and the corresponding -log10(FDR) values (horizontal axis) are plotted on the enrichment maps. The enrichment strength is mapped to the size of the dots. Different sets of conditions are colored as indicated. GSEA was performed for gene sets that were either commonly upregulated (A) or downregulated (B) by both drugs, or upregulated only by RTX (C) or 5FdUR (D) treatments in the three cell lines independently (cf. Venn diagrams in S1 Fig). The gene lists and all details are provided in Source Data File 1 (Source-data_Fig4_S2Fig_S3Fig.xlsx). Permanent links for the corresponding STRING networks and network statistics are given in Table 1. (TIF) [file pone.0332491.s003.tif]

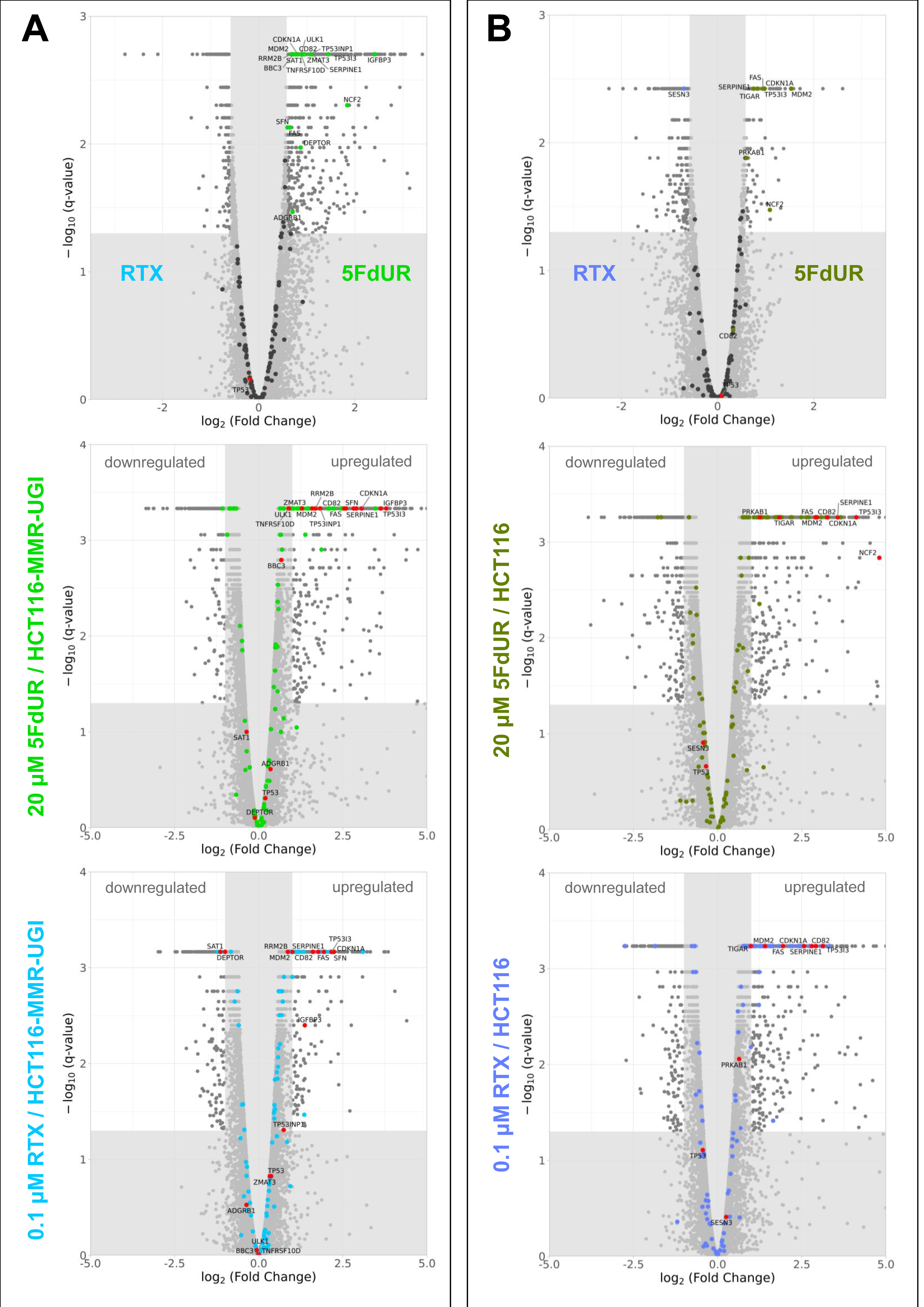

Supplement: S4 Fig — DE mRNAs in HCT116-MMR-UGI (A) and HCT116 (B) cell lines are presented on Volcano-plots: direct comparisons of the two drug treatments (0.1 μM RTX and 20 μM 5FdUR, top plots, the same as in Fig 3C and D), and the two treatments compared to the corresponding NT samples (mid and bottom plots, the same as S1B and S1C Figs). In the top plots, the p53-related genes from the WikiPathways (WP4963), and the KEGG Pathways (hsa04115) are colored either with dark gray (not significantly differentially expressed), or shades of green (5FdUR-biased) or blue (RTX-biased) following the previously applied color code. TP53 (encoding p53) is marked with red. In the bottom plots, components of the same p53-related pathways are colored according to the color code previously applied for the different conditions, and those that displayed drug-biased differential expression are marked in red. (TIF) [file pone.0332491.s004.tif]

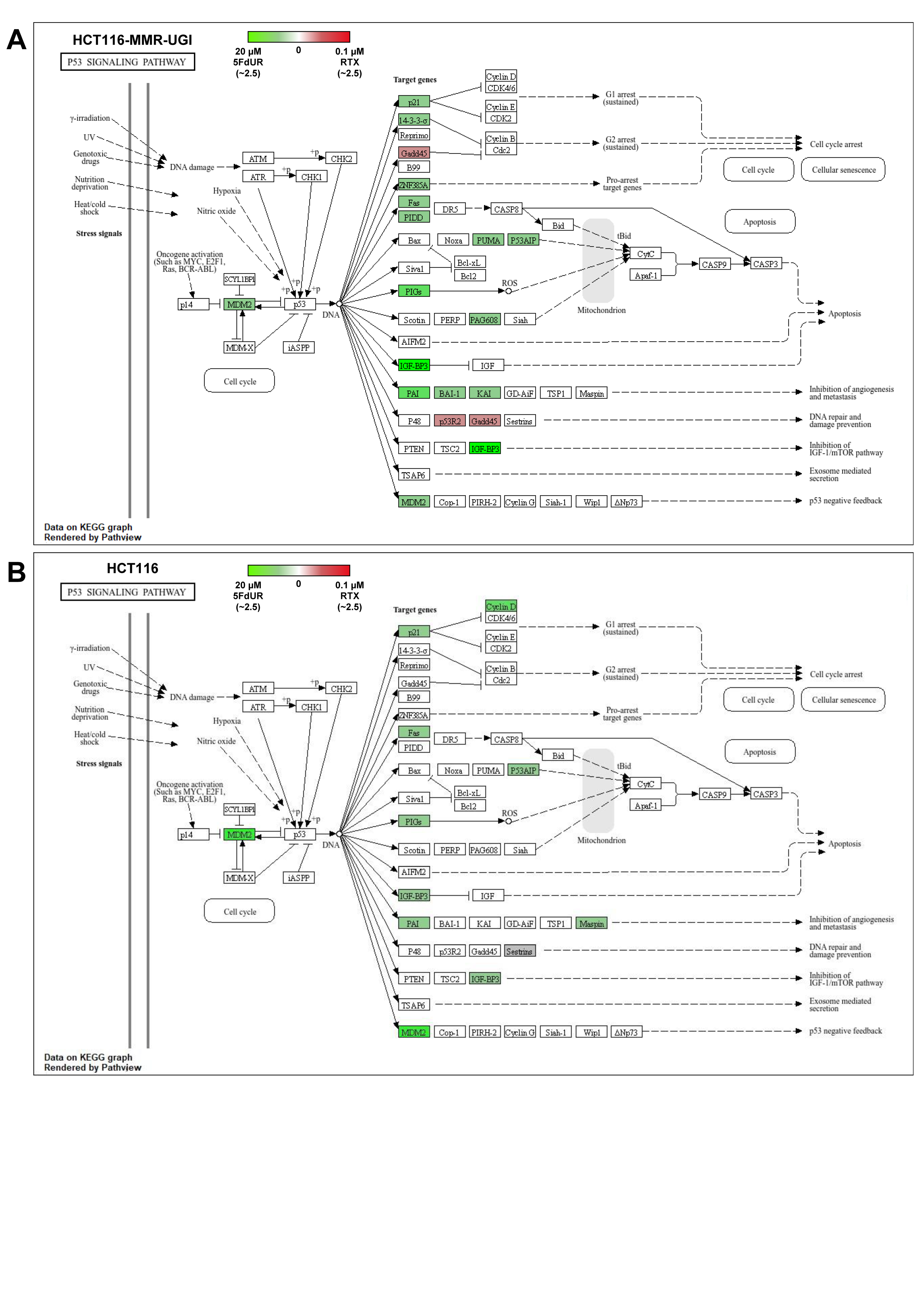

Supplement: S5 Fig — Using the clusterProfiler, the hsa04115 pathway from the KEGG Pathways was colored according to differential expression data comparing the two drug treatments in (A) HCT116-MMR-UGI and (B) HCT116 cells. The colors of the network nodes are mapped to the log2(Fold Change) values according to the color bar from 5FdUR-biased (green) to RTX-biased (red) expression. White boxes represent non-significant drug bias, gray represents multiple genes behind the network nodes. (TIF) [file pone.0332491.s005.tif]

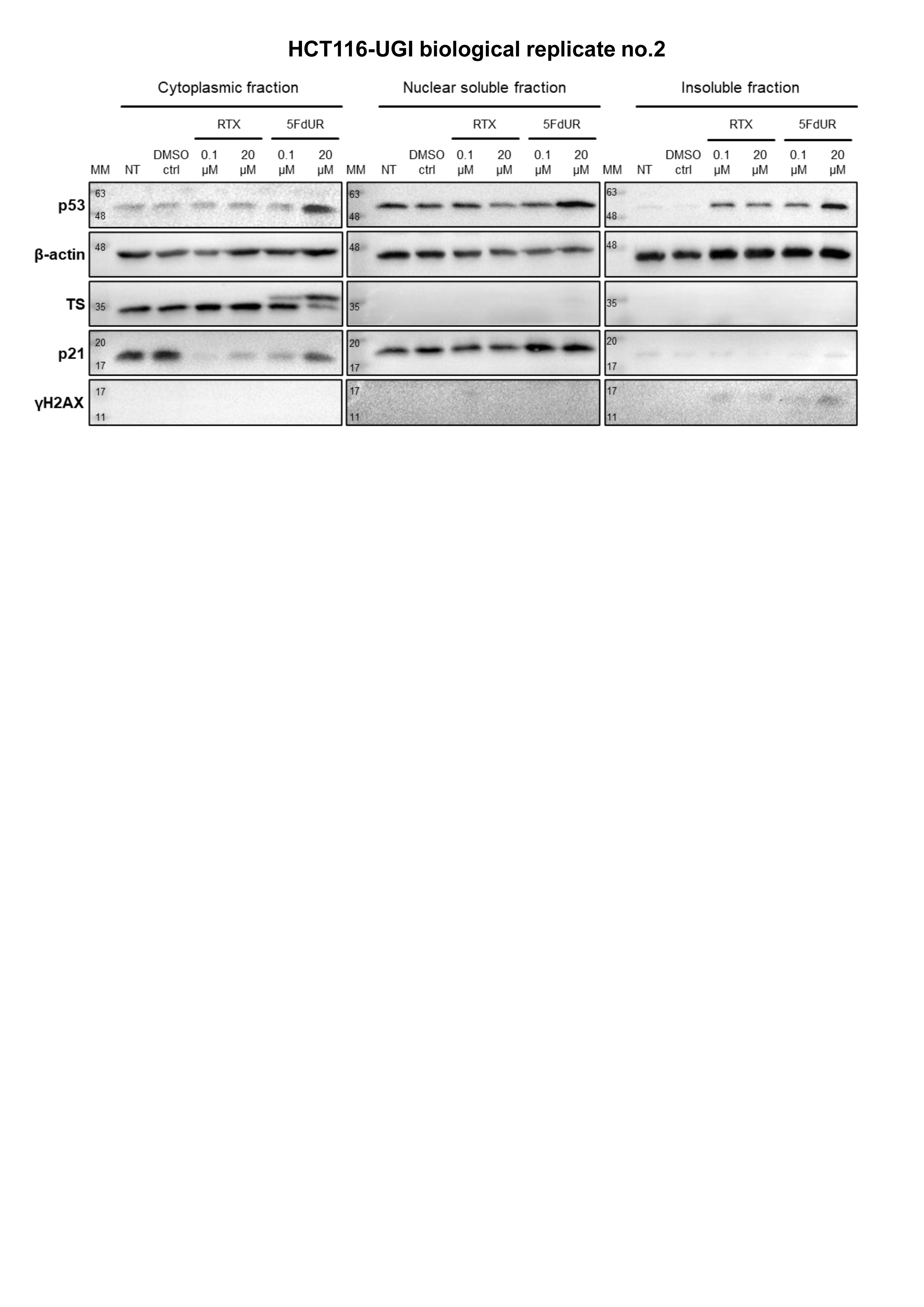

Supplement: S6 Fig — The uncropped images of each blot are provided in the Source Data File 3 (Source-data_Uncropped-Western-blots.pdf). (TIF) [file pone.0332491.s006.tif]

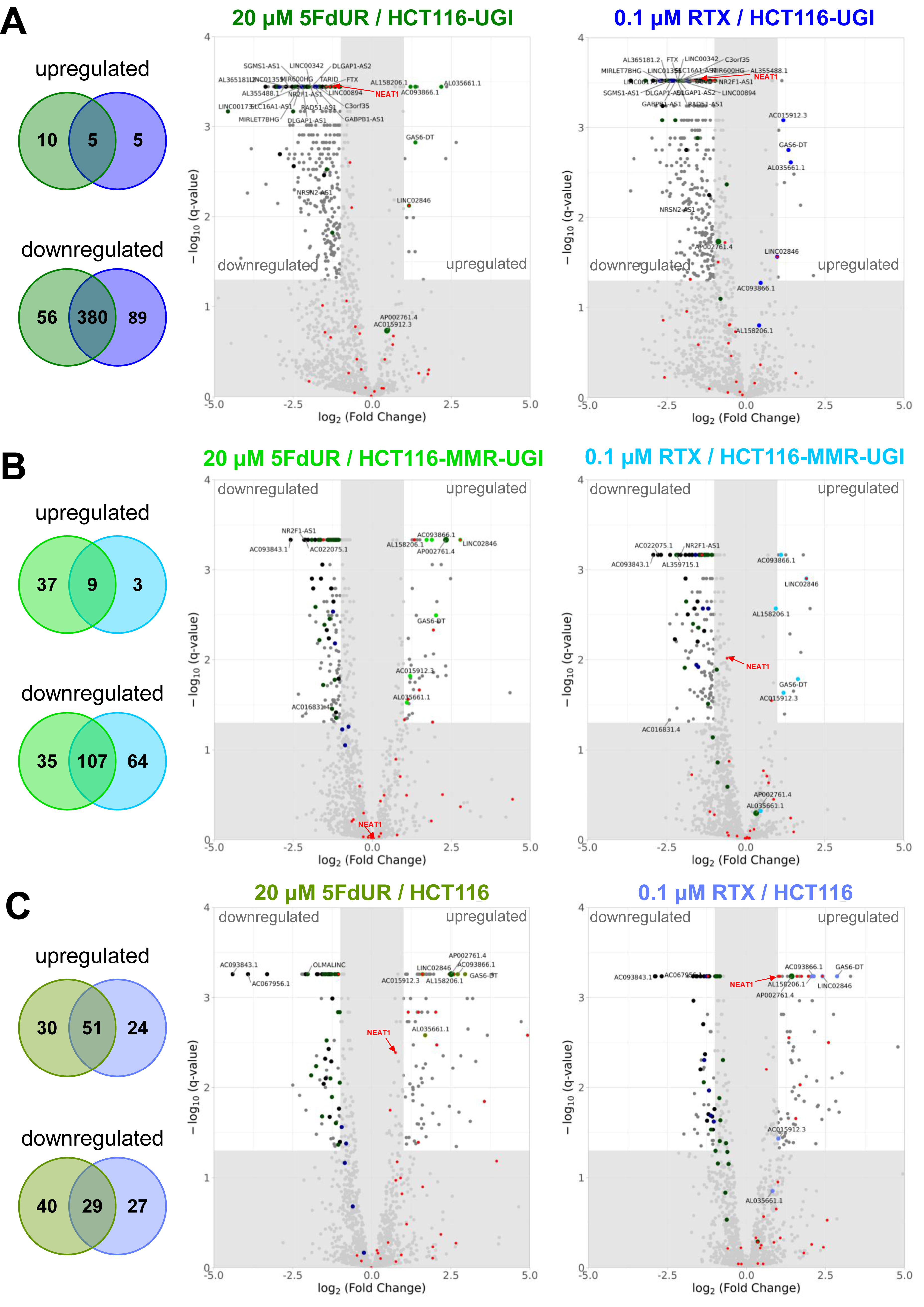

Supplement: S7 Fig — Treatment-induced differential expression was calculated together with protein-coding genes (cf. S1 Fig). The numbers of DE lncRNA genes are summarized in Venn diagrams (left). Significant DE lncRNA genes (fold change ≥ 2, q-value < 0.05, and mean FPKM > 1000) are shown on Volcano-plots with dark gray on a white background. Those that were commonly upregulated in the three cell lines in response to either RTX or 5FdUR treatments are colored using the condition-specific color code and labeled by their gene symbol. The only lncRNA gene that shows 5FdUR-biased expression in all three cell lines, AP002761.4 (enlarged green dot), is labeled on each Volcano plot. Commonly downregulated lncRNA genes in all six conditions (black), in the three RTX-treated (dark blue), and in the three 5FdUR-treated (dark green) cell lines are also marked. Pronounced downregulated lncRNA genes (expression level in NT cells ≥ 10.000 FPKM, and log2(Fold Change) values < −2) are labeled with their gene symbols. On panel A, pronounced downregulated lncRNA genes without known functions are not labeled. lncRNAs that are known targets of p53 are labeled with smaller red dots. (TIF) [file pone.0332491.s007.tif]

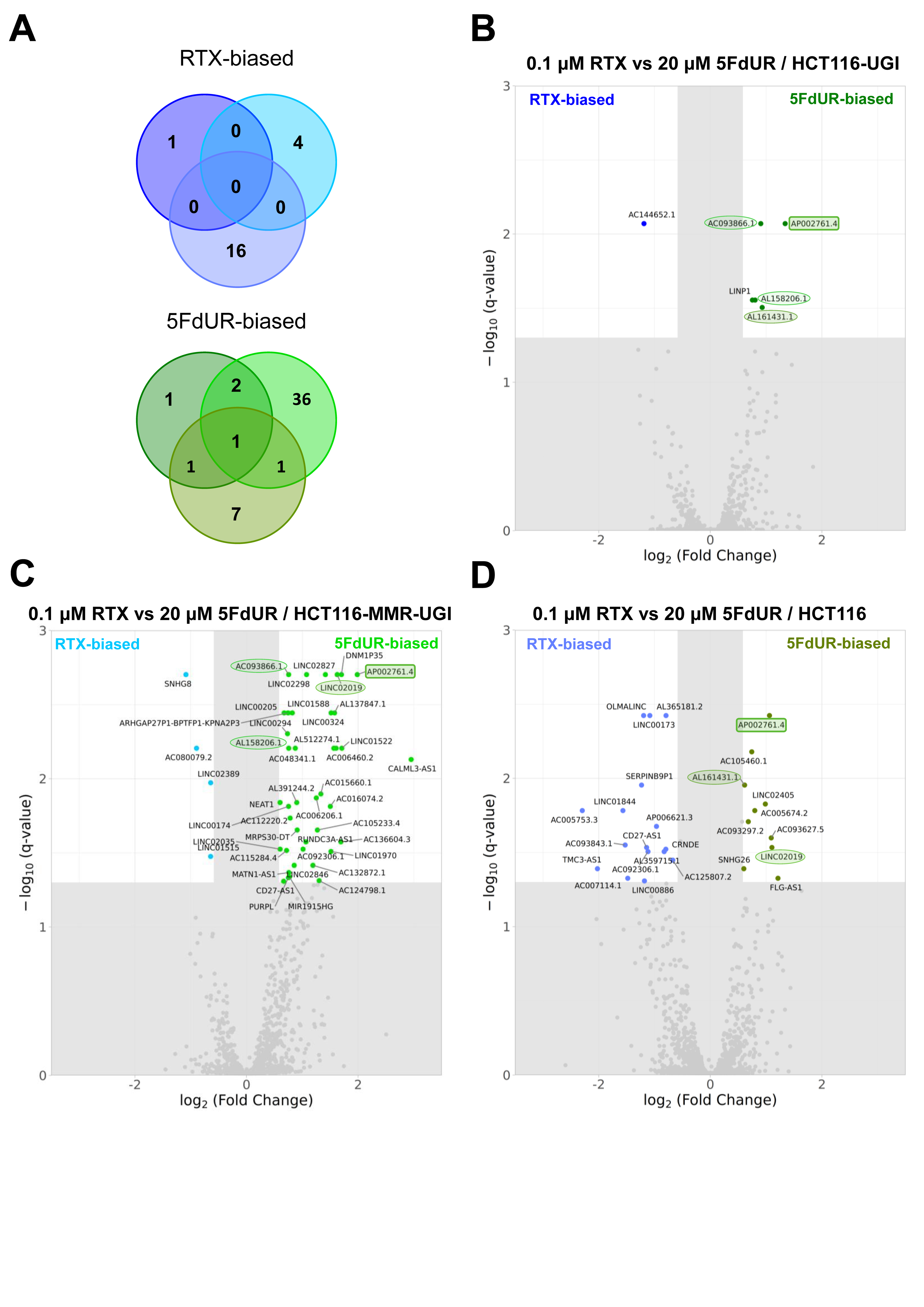

Supplement: S8 Fig — A direct comparison of the two drug effects in lncRNA expression data is presented in Venn diagrams (A), and by Volcano plots for HCT116-UGI (B), HCT116-MMR-UGI (C), and HCT116 (D) cell lines. 5FdUR-bised lncRNAs common across cell lines are highlighted with rectangular (all cell lines) or oval (2 cell lines) shapes. (TIF) [file pone.0332491.s008.tif]

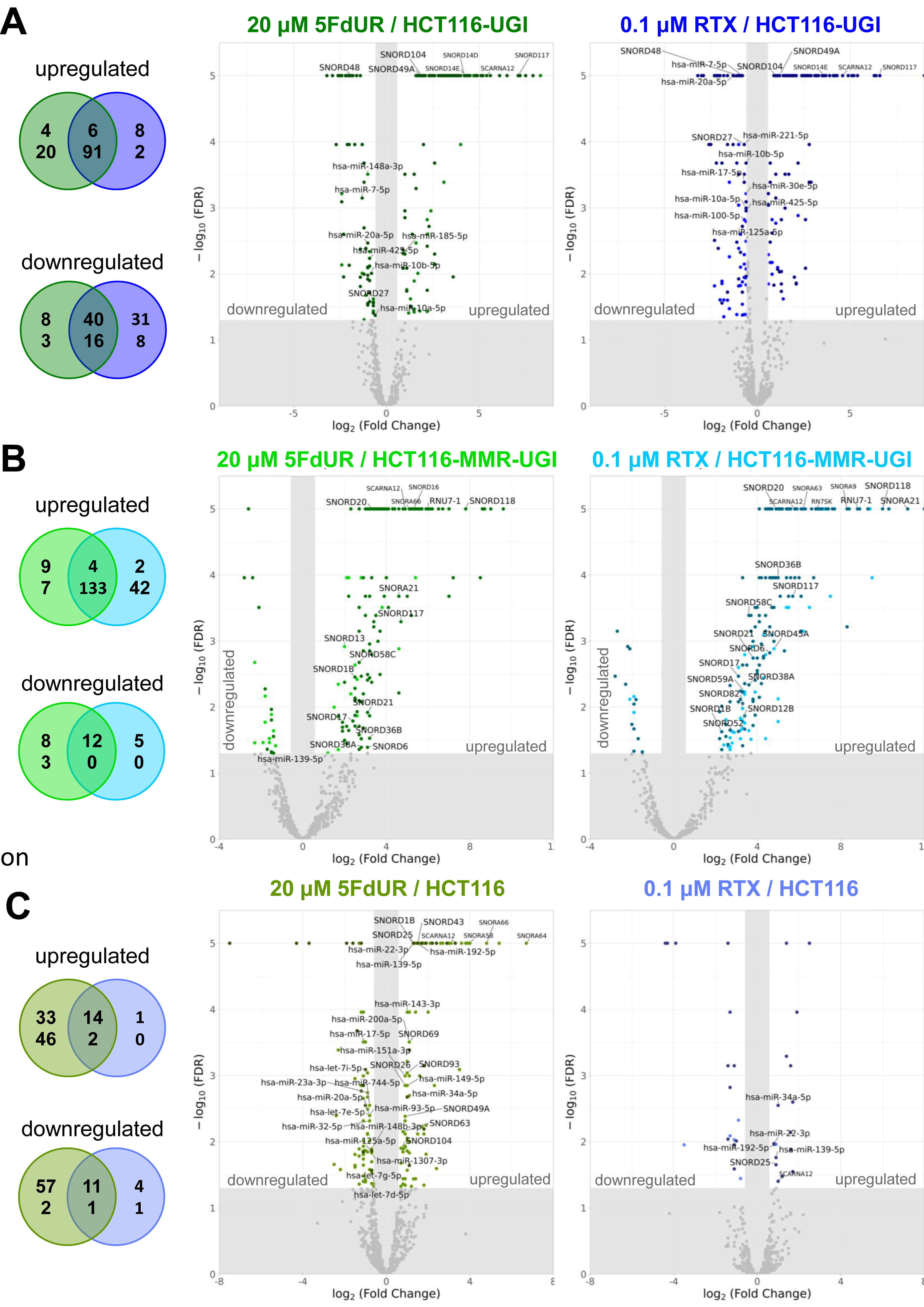

Supplement: S9 Fig — Treatment-induced differential expression of short RNAs (including miRNAs and sn/snoRNAs) was calculated using featureCounts and deseq2 as described in Materials and Methods. The numbers of DE miRNA (top numbers) and sRNA (bottom numbers) genes are summarized in Venn diagrams (left). Significant DE mi/sRNA genes (fold change ≥ 1.5, q-value < 0.05, and mean counts > 25) are shown on Volcano-plots colored using the condition-specific color code on a white background. Those genes that were commonly up- or downregulated by both drugs in the given cell line are marked with darker colors, and abundant ones are labeled by their gene names (mean counts above 10k (bigger font size) or between 1k and 10 k (smaller font size)). (TIF) [file pone.0332491.s009.tif]

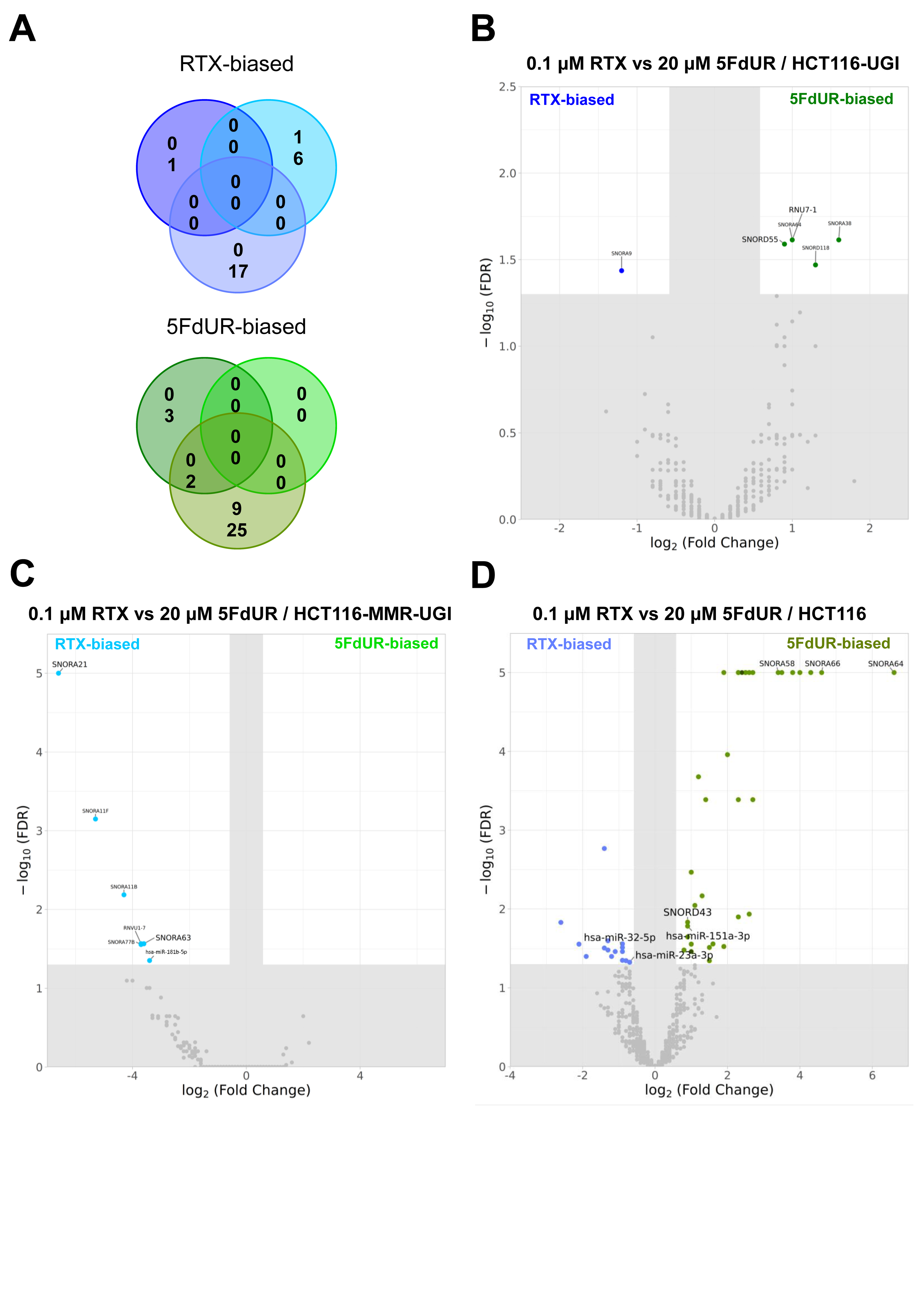

Supplement: S10 Fig — A direct comparison of the two drug effects in short RNA expression data is presented in Venn diagrams (A), and by Volcano plots for HCT116-UGI (B), HCT116-MMR-UGI (C), and HCT116 (D) cell lines. Significant DE mi/snoRNA are colored according to the previously applied condition-specific color code and labeled by their gene names. The font size of the labels reflects the abundance of these genes: (mean counts above 10k (bigger font size, only on panel D), between 1k and 10 k (smaller font size), or between 100 and 1k (smallest size, on panels B and C). (TIF) [file pone.0332491.s010.tif]

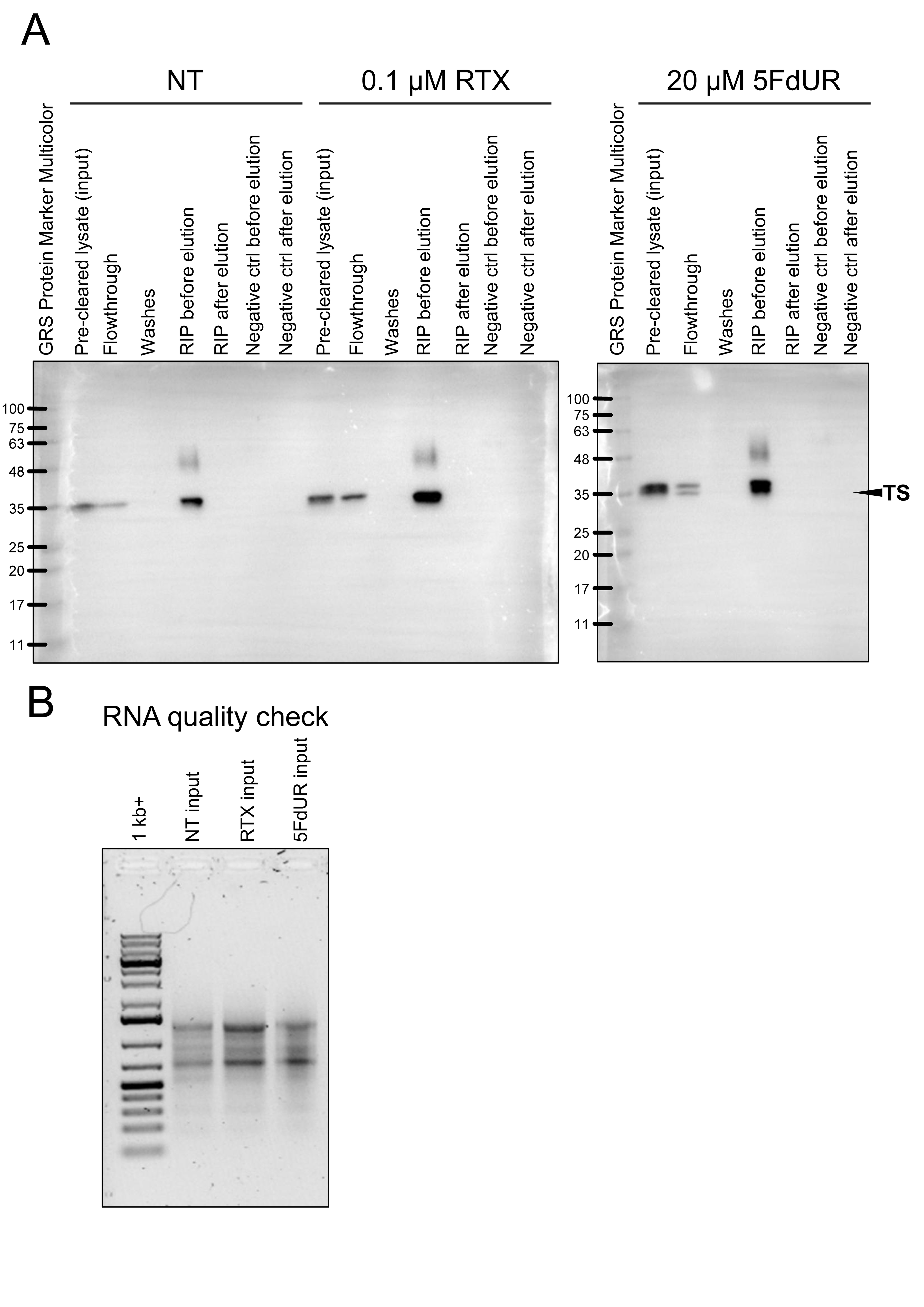

Supplement: S11 Fig — (A) Western blotting on TS-RIP fractions. Samples from each step are indicated above the blot. Molecular mass marker: GRS Protein Marker MultiColour (GRISP Research Solutions, Porto, Portugal), “RIP before elution” indicates the antibody-conjugated beads that successfully recruited TS protein from the pre-cleaned lysates (NT, RTX, 5FdUR samples as indicated at the top. “Negative ctrl” samples represent protein A/G agarose incubated with lysates without anti-TS antibody. Position of TS (~35.7 kDa) is marked (triangle on the right). (B) Quality of input RNA analyzed on 1% agarose gel. The input samples were incubated under the same conditions as the TS-RIP and negative control samples. The two main bands correspond to abundant rRNAs, indicating the extent of their preservation during the long immunoprecipitation procedure. (TIF) [file pone.0332491.s011.tif]
