# Supplementary material for: Thymidylate synthase inhibitory drugs induce p53-dependent pathways differently
Source: PLoS One. 2026 Jul 1;21(7):e0332491. doi: 10.1371/journal.pone.0332491 (PMC13322534; doi:10.1371/journal.pone.0332491)
Supplement: S1 File — (DOCX) [file pone.0332491.s014.docx]

**Supplementary Methods**

**Thymidylate synthase inhibitory drugs induce p53-dependent pathways differently.**

**Authors**

Eszter Holub^1,2,3^, Milda Blanka Szajkó^1,2^, Anna Felföldi^1^, Beáta G. Vértessy^1,2^*, Angéla Békési ^1,2^*

**Authors’ affiliations**

^1^ Department of Applied Biotechnology and Food Science, Faculty of Chemical Technology and Biotechnology at Budapest University of Technology and Economics, Budapest, Hungary

^2^ Genome Metabolism Research Group, Institute of Molecular Life Sciences at HUN-REN Research Center for Natural Sciences, Budapest, Hungary

^3^ Doctoral School of Biology, Institute of Biology at ELTE Eötvös Loránd University, Budapest, Hungary

* Corresponding author

E-mail: [bekesi.angela@vbk.bme.hu](mailto:bekesi.angela@vbk.bme.hu) (AB)

* Second corresponding author:

E-mail: [vertessy.beata@ttk.hu](mailto:vertessy.beata@ttk.hu) (BGV)

**Running Title:** Transcriptomic effects of TS-inhibitors

**Keywords:** 5-fluoro-2’-deoxyuridine, raltitrexed, thymidylate synthase, p53-related, transcriptome sequencing, RNA-IP-seq

***RNA-seq analysis***

Sequencing quality of raw data was checked using the fastQC (1). Adapter and quality **trimming** were done using fastp (2):

mkdir TrimmedData

cd TrimmedData

for f in ../rawData/*_1.fastq.gz

do

tsamp="$(basename $f _1.fastq.gz)"

dir="$(dirname $f)"

#cut artificial polyG ends due to too short insert size.

fastp --in1 $dir/${tsamp}_1.fastq.gz --in2 $dir/${tsamp}_2.fastq.gz \

--out1 ./${tsamp}pre_P1.fq.gz --out2 ./${tsamp}pre_P2.fq.gz \

--unpaired1 ./${tsamp}pre_U1.fq.gz --unpaired2 ./${tsamp}pre_U2.fq.gz \

--dont_overwrite --disable_adapter_trimming --trim_poly_g \

--poly_g_min_len 2 -j ./${tsamp}pre.trimmed.json \

-h ./${tsamp}pre.trimmed.html

#cut adapter-related and low-quality sequences, and also potential polyA tails

fastp --in1 ./${tsamp}pre_P1.fq.gz --in2 ./${tsamp}pre_P2.fq.gz \

--out1 ./${tsamp}_P1.fq.gz --out2 ./${tsamp}_P2.fq.gz \

--unpaired1 ./${tsamp}_U1.fq.gz --unpaired2 ./${tsamp}_U2.fq.gz \

--dont_overwrite --detect_adapter_for_pe --adapter_fasta adapter2.fa \

-l 20 -q 20 -u 30 -x --poly_x_min_len 2 -f 12 --dont_eval_duplication \

-j ./${tsamp}.trimmed.json -h ./${tsamp}.trimmed.html \

-R "${tsamp}_trimming_report"

#quality check for trimmed files

fastqc -o ./FastQC_trimmed -t 32 -f fastq ./${tsamp}_P1.fq.gz \

./${tsamp}_P2.fq.gz ./${tsamp}_U1.fq.gz ./${tsamp}_U2.fq.gz \

./${tsamp}pre_P1.fq.gz ./${tsamp}pre_P2.fq.gz ./${tsamp}pre_U1.fq.gz \

./${tsamp}pre_U2[.fq.gz](https://encoded-592c9deb-987b-4562-aa3c-9fa3d37d83e9.uri/http%3a%2f%2f.fq.gz)

done

**Removal of rRNA-related reads** was performed in silico by aligning raw data to the reference set of rRNA genes using BWA (3), then transforming the unmapped reads from the BAM file back to fastq format:

mkdir rRNAdepletion

cd rRNAdepletion

for f in ../TrimmedData/*_P1.fq.gz

do

tsamp="$(basename $f _P1.fq.gz)"

dir="$(dirname $f)"

bwa mem -t 32 \

-R '@RG\tID:01\tSM:SAMPLE' /PATH/rRNA_seqs_nonredundant.fasta \

$dir/${tsamp}_P1.fq.gz $dir/${tsamp}_P2.fq.gz | samtools view -Shb | \

samtools sort -@ 32 > ./$tsamp.rRNA.bam

samtools index -@ 32 ./$tsamp.rRNA.bam

samtools idxstats -@ 32 ./$tsamp.rRNA.bam > ./$tsamp.rRNA.idxstats.csv

samtools view -@ 32 -f4 -Shb ./$tsamp.rRNA.bam > \

./$tsamp.rRNAdepleted.bam

bedtools bamtofastq -i ./$tsamp.rRNAdepleted.bam \

-fq ./$tsamp.rRNAdepleted.P1.fq -fq2 $tsamp.rRNAdepleted.P2.fq

gzip ./$tsamp.rRNAdepleted.P1.fq

gzip ./$tsamp.rRNAdepleted.P2.fq

rm $tsamp.rRNAdepleted.bam

done

Quality check was performed after rRNA depletion using fastQC (1), and the rRNA-depleted reads were aligned to the human reference genome GRCh38 (4) using hisat2 tool (5):

mkdir alignedData

cd alignedData

for f in ../rRNAdepletion/*.rRNAdepleted.P1.fq.gz

do

tsamp="$(basename $f .P1.fq.gz)"

dir="$(dirname $f)"

hisat2 --phred33 --rna-strandness RF --no-discordant --add-chrname \

--rg-id $tsamp -p 32 -x /PATH/grch38_tran/genome_tran -1 \

$dir/$tsamp.P1.fq.gz -2 $dir/$tsamp.P2.fq.gz -S ./$tsamp.unsorted.sam

samtools view -@ 32 -Shb ./$tsamp.unsorted.sam | samtools sort -@32 > \

./$tsamp.sorted.bam

samtools index -@ 32 ./$tsamp.sorted.bam

samtools idxstats ./$tsamp.sorted.bam > \

./$tsamp.sorted.bam.idxstats.csv

rm ./$tsamp.unsorted.sam

done

Raw **aligned data were filtered** for uniquely mapped reads and the core chromosomes. Furthermore, in the case of the TS-RIP data, a sub-sampling was also performed to generate pseudoreplicates for statistical evaluation:

for f in ./*.rRNAdepleted.sorted.bam

do

tsamp="$(basename $f .rRNAdepleted.sorted.bam)"

samtools view -bh -q1 -@32 -L list_of_core_chr.bed \

$tsamp.rRNAdepleted.sorted.bam > $tsamp.core.rRNAdepleted.sorted.bam

samtools view -bh -q1 -@32 -L list_of_core_chr.bed -s 0.5 \

$tsamp.rRNAdepleted.sorted.bam > $tsamp.coreA50.rRNAdepleted.sorted.bam

samtools index -@ 32 ./$tsamp.core.rRNAdepleted.sorted.bam

samtools idxstats ./$tsamp.core.rRNAdepleted.sorted.bam > \

./$tsamp.core.rRNAdepleted.sorted.bam.idxstats.csv

#For generating pseudoreplicates:

samtools index -@ 32 ./$tsamp.coreA50.rRNAdepleted.sorted.bam

samtools idxstats ./$tsamp.coreA50.rRNAdepleted.sorted.bam > \

./$tsamp.coreA50.rRNAdepleted.sorted.bam.idxstats.csv

done

Calculations of **expression levels and differential expression** were done using the Cufflinks package (6,7) for the longRNA-seq and the TS-RIP-seq data. This approach allows a reconstruction of the transcriptome based on the split reads and the given annotation GTF file, then the establishment of a common (merged) reference transcriptome, on which a quantification and several comparisons can be performed:

mkdir cufflinks

cd cufflinks

for f in ../alignedData/.core*.rRNAdepleted.sorted.bam

do

tsamp="$(basename $f .rRNAdepleted.sorted.bam)"

dir="$(dirname $f)"

cufflinks -o $tsamp.cufflinks -p 32 -G /PATH/basicgencodev34.gtf -u \

--library-type fr-firststrand $dir/$tsamp.rRNAdepleted.sorted.bam

done

cuffmerge -o cuffmerged -g /PATH/basicgencodev34.gtf -p 32 list_of_transcripts_gtf

#list_of_transcripts_gtf is a list of cufflinks output files: $tsamp.cufflinks /transcript.gtf

for f in ../alignedData/.core*.rRNAdepleted.sorted.bam

do

tsamp="$(basename $f .rRNAdepleted.sorted.bam)"

dir="$(dirname $f)"

cuffquant -o $tsamp.cuffquant -u --library-type fr-firststrand -p 32 \

--no-length-correction ./cuffmerged/merged.gtf \

$dir/$tsamp.rRNAdepleted.sorted.bam

done

cuffnorm -o cuffnorm_All -L NT_lRNA,RTX_lRNA,5FdUR_lRNA,NT_RIPctr,RTX_RIPctr,5FdUR_RIPctr,NT_RIP,RTX_RIP,5FdUR_RIP \

--library-norm-method geometric -p 32 ./cuffmerged/merged.gtf \

NT_rep1.lRNA.core.cuffquant/abundances.cxb,NT_rep2.lRNA.core.cuffquant/abundances.cxb \

RTX_rep1.lRNA.core.cuffquant/abundances.cxb,RTX_rep2.lRNA.core.cuffquant/abundances.cxb \

5FdUR_rep1.lRNA.core.cuffquant/abundances.cxb,5FdUR_rep2.lRNA.core.cuffquant/abundances.cxb \

NT_RIPctr.core.cuffquant/abundances.cxb,NT_RIPctr.coreA50.cuffquant/abundances.cxb \

RTX_RIPctr.core.cuffquant/abundances.cxb,RTX_RIPctr.coreA50.cuffquant/abundances.cxb \

5FdUR_RIPctr.core.cuffquant/abundances.cxb,5FdUR_RIPctr.coreA50.cuffquant/abundances.cxb \

NT_TS_RIP.core.cuffquant/abundances.cxb,NT_TS_RIP.coreA50.cuffquant/abundances.cxb \

RTX_TS_RIP.core.cuffquant/abundances.cxb,RTX_TS_RIP.coreA50.cuffquant/abundances.cxb \

5FdUR_TS_RIP.core.cuffquant/abundances.cxb,5FdUR_TS_RIP.coreA50.cuffquant/abundances.cxb

#To **compare** the RTX- or 5FdUR-treated samples to the NT sample example for RTX-NT comparison:

cuffdiff -o NT_vs_RTX.cuffdiff -L NT_lRNA,RTX_lRNA --FDR 0.05 -u \

-p 32 --no-length-correction ./cuffmerged/merged.gtf \

NT_rep1.lRNA.core.cuffquant/abundances.cxb,\

NT_rep2.lRNA.core.cuffquant/abundances.cxb \

RTX_rep1.lRNA.core.cuffquant/abundances.cxb,\

RTX_rep2_lRNA.core.cuffquant/abundances.cxb

#To compare the TS-RIP samples to the corresponding RIPctr samples; example for RTX sample:

cuffdiff -o RTX_RIPctr_vs_TS-RIP.cuffdiff -L RTX_RIPctr,RTX_TS-RIP \

--FDR 0.05 -u -p 32 --no-length-correction ./cuffmerged/merged.gtf \

RTX_TS_RIPctr.core.cuffquant/abundances.cxb,\

RTX_TS_RIPctr.coreA50.cuffquant/abundances.cxb \

RTX_TS_RIP2.core.cuffquant/abundances.cxb,\

RTX_TS_RIP2.coreA50.cuffquant/abundances.cxb

Both the cuffnorm and the cuffdiff outputs were **merged with annotation** based on **gencode V34** (<https://www.gencodegenes.org/human/release_34.html>, (8):

##### # Creation of isoforms.attr_table.GeneCodeV34.csv and gene.attr_table.GeneCodeV34.csv

# awk '($3=="transcript") {print $0}' gencode.v34.basic.annotation.gtf | tr -d '";' | awk '{print $12 "\t" $10 "\t" $14 "\t" $16 " \t" $18 "\t" $20 "\t" $26 "\t" $1 "\t" $4 "\t" $5 "\t" $7 "\t" $2}' | sort -k1,1 > gencode.v34.basic.transcript_annotation.csv

# The cuffnorm output was used to join the inner test IDs to the annotation file:

sort -k3,3 ../cuffnorm/isoforms.attr_table > isoforms.attr_table.sorted

join --header -e who_knows -1 3 -2 1 isoforms.attr_table.sorted gencode.v34.basic.transcript_annotation.csv > isoforms.attr_table.GeneCodeV34.csv

awk '{print $0 "\t" $4}' isoforms.attr_table.GeneCodeV34.csv | sort –k4,4 | uniq -f19 > gene.attr_table.GeneCodeV34.csv

#cleaning the gene.attr_table.GeneCodeV34.csv, keeping the meaningful columns only

# A headline is also inserted: “ENST test_id relation tracking_id ENST2 tss_id locus length gene_id biotype gene_symbol transcript_type transcript_id X chr start end strand origin tracking_id2"

join -1 1 -2 4 --header -e who_knows ../cuffnorm/genes.attr_table gene.attr_table.GeneCodeV34.csv | awk '{print $1 "\t" $16 "\t" $18 "\t" $17 "\t" $7 "\t" $25 "\t" $5 "\t" $6}' | sort -k1,1 > gene.attr_table.GeneCodeV34.clean.csv

wc -l gene.attr_table.GeneCodeV34.clean.csv

42823 gene.attr_table.GeneCodeV34.clean.csv

for f in ./*/gene_exp.diff

do

tsamp="$(dirname $f)"

sort -k1,1 $tsamp/gene_exp.diff > $tsamp.gene_exp.diff

join --header -e who_knows -1 1 -2 1 $tsamp.gene_exp.diff \

gene.attr_table.GeneCodeV34.clean.csv | awk '{print $1 "\t" $15 "\t" $16 "\t" $17 "\t" $18 "\t" $19 "\t" $5 "\t" $6 "\t" $7 "\t" $8 "\t" $9 "\t" $10 "\t" $11 "\t" $12 "\t" $13 "\t" $14}' > $tsamp.gene_exp.GeneCodeV34.diff

awk '($0~/yes/) && ($12>0) {print $0}' $tsamp.gene_exp.GeneCodeV34.diff \

> $tsamp.list.GeneCodeV34.DEgenes_pos.txt

awk '($0~/yes/) && ($12<0) {print $0}' $tsamp.gene_exp.GeneCodeV34.diff \

> $tsamp.list.GeneCodeV34.DEgenes_neg.txt

done

join -1 1 -2 1 --header -e who_knows ../cuffnorm/genes.fpkm_table gene.attr_table.GeneCodeV34.clean.csv > cuffnorm.genes.fpkm_table.fullAnnot.GeneCodeV34.csv

**Vulcano plots in R:**

install.packages("dplyr")

install.packages("ggrepel")

install.packages("ggplot2")

#head deseq2.NTvsRTX_sRNA.csv

#Geneid,ensembl_gene_id,ensembl_transcript_id,transcript_length,X5FdUR_rep2_sRNA,X5FdUR_rep3_sRNA,baseMean,baseMeanA,baseMeanB,foldChange,log2FoldChange,lfcSE,stat,PValue,PAdj,FDR,falsePos,NT_rep2_sRNA,NT_rep3_sRNA,RTX_rep2_sRNA,RTX_rep3_sRNA

# Now read in the data file with header:

diff_data <- read.csv("deseq2.NTvsRTX_sRNA.csv", sep = ",", header = TRUE)

#counting with FDR is problematic, as it is many times =0, so the logarithm will not work. Therefore, a pseudocount 0.00001 was introduced, as the lowest non-zero FDR value was 0.0001

diff_data$log_FDR = -1*log10((diff_data$FDR)+0.00001)

diff_data$Significant <- ifelse(diff_data$log_FDR > 1.30103 & abs(diff_data$log2FoldChange) > 0.585 , "Significant", "Not_Significant")

write.table(diff_data, file="RTX_vs_NT.deseq2_DE_sRNA.volcano.csv")

library(dplyr)

#Filter for genes with meanCounts > 50

filtered_data <- diff_data %>%

filter(baseMean > 50)

#get the top genes according to combined filters

top_genes_comb <- filtered_data %>%

filter(abs(log2FoldChange) > 1, log_FDR > 2)

# Get the top 5 genes by FDR-value

#top_genes <- filtered_data %>%

# arrange(FDR) %>%

# slice_head(n = 5)

# Get the top 5 genes by log2FC

#top_genes <- filtered_data %>%

# arrange(FDR) %>%

# filter(abs(log2FoldChange) > 1) %>%

# slice_head(n = 5) %>%

#Top 5 up- and down-regulated genes

top_genes <- bind_rows(

filtered_data %>%

arrange(desc(log_FDR)) %>%

filter(abs(log2FoldChange) > 0.585, log_FDR > 1.31) %>%

slice_head(n = 10),

filtered_data %>%

arrange(desc(abs(log2FoldChange))) %>%

filter(log_FDR > 1.31) %>%

slice_head(n = 40)

) %>%

distinct(Geneid, .keep_all = TRUE) # remove duplicates based on Geneid

#Manually selected genes are labeled

top_genes_man <- filtered_data %>% filter(Geneid %in% c("RNU7-1", "SNORA38", "SNORA64", "SNORD55", "SNORD118", "SNORD104", "SNORA9", "AC025362.1:SNORA50C"))

library(ggrepel)

library(ggplot2)

plot1 <- ggplot(filtered_data, aes(x = log2FoldChange, y = log_FDR)) +

geom_point(aes(color = Significant)) +

scale_x_continuous(limits = c(-5, 5),expand=c(0,0)) +

scale_y_continuous(limits=c(0,4.6),expand=c(0,0)) +

scale_color_manual(values = c("gray", "red")) +

guides(color = "none") +

ggtitle(expression("DE sRNAs, 0.1 "~mu*"M RTX (featureCounts/deseq2)")) +

labs(x = expression(log[2]~"(Fold Change)"), y = expression(-log[10]~"(FDR)")) +

theme_light() + geom_rect(aes(xmin=-0.585,xmax=0.585,ymin=1.30103,ymax=Inf),alpha=0.02,fill="grey99") +

geom_rect(aes(xmin=-Inf,xmax=Inf,ymin=0,ymax=1.30103),alpha=0.02,fill="grey99") +

theme(text = element_text(size=12)) +

geom_text_repel(

data = top_genes_comb,

aes(label = Geneid),

size = 3,

max.overlaps = 30,

box.padding = 0.2,

segment.color = "grey50"

)

ggsave("RTX_vs_NT.deseq2_DE_sRNA_filt.volcano.png", plot1, dpi = 600)

plot_man <- ggplot(filtered_data, aes(x = log2FoldChange, y = log_FDR)) +

geom_point(aes(color = Significant)) +

scale_x_continuous(limits = c(-5, 5),expand=c(0,0)) +

scale_y_continuous(limits=c(0,4.6),expand=c(0,0)) +

scale_color_manual(values = c("gray", "red")) +

guides(color = "none") +

ggtitle(expression("DE sRNAs, 0.1 "~mu*"M RTX (featureCounts/deseq2)")) +

labs(x = expression(log[2]~"(Fold Change)"), y = expression(-log[10]~"(FDR)")) +

theme_light() + geom_rect(aes(xmin=-0.585,xmax=0.585,ymin=1.30103,ymax=Inf),alpha=0.02,fill="grey99") +

geom_rect(aes(xmin=-Inf,xmax=Inf,ymin=0,ymax=1.30103),alpha=0.02,fill="grey99") +

theme(text = element_text(size=12)) +

geom_text_repel(

data = top_genes_man,

aes(label = Geneid),

size = 3,

max.overlaps = 30,

box.padding = 0.2,

segment.color = "grey50"

)

ggsave("RTX_vs_NT.deseq2_DE_sRNA_filt.man_label.volcano.png", plot_man, dpi = 600)

**Heatmap in R:**

pheatmap (version 1.0.13) was used.

library(pheatmap)

## User-defined inputs (edit these)

input_dir <- "<PATH/TO/INPUT_DIRECTORY>"

output_dir <- "<PATH/TO/OUTPUT_DIRECTORY>"

dir.create(output_dir, showWarnings = FALSE, recursive = TRUE)

files <- c(

"protein_coding_1000_10000.tsv", "protein_coding_10000_100000.tsv", "protein_coding_100000_plus.tsv", "lncRNA_onlysamples.tsv")

output_files <- c("heatmap1.png", "heatmap2.png", "heatmap3.png", "heatmap4.png")

# Desired column order (sample order)

desired_order <- c(

"WT_lRNA_0", "WT_lRNA_1",

"RTX_lRNA_0", "RTX_lRNA_1",

"X5FdUR_lRNA_0", "X5FdUR_lRNA_1",

"NT_UGI_lRNA_0", "NT_UGI_lRNA_1",

"RTX_UGI_lRNA_0", "RTX_UGI_lRNA_1",

"X5FdUR_UGI_lRNA_0", "X5FdUR_UGI_lRNA_1",

"NT_UGIMMR_lRNA_0", "NT_UGIMMR_lRNA_1",

"RTX_UGIMMR_lRNA_0", "RTX_UGIMMR_lRNA_1",

"X5FdUR_UGIMMR_lRNA_0", "X5FdUR_UGIMMR_lRNA_1")

# Transformation and plotting parameters

pseudocount <- 10

zlim <- 2 # color scale clipped to [-zlim, +zlim]

png_width <- 4000

png_height <- 5000

png_res <- 300

## Processing loop

for (i in seq_along(files)) {

in_file <- file.path(input_dir, files[i])

out_file <- file.path(output_dir, output_files[i])

# Input: tab-delimited table with header; first column used as row names

data <- read.table(in_file, header = TRUE, sep = "\t", row.names = 1, check.names = FALSE)

# Reorder columns to a fixed sample order

data <- data[, desired_order]

# Row-wise baseline normalization:

# log2( (x + pseudocount) / mean_row(x + pseudocount) )

row_means <- rowMeans(data + pseudocount)

data_scaled <- log2((data + pseudocount) / row_means)

# Heatmap with fixed color scale [-zlim, +zlim] (values outside are clipped)

png(out_file, width = png_width, height = png_height, res = png_res)

pheatmap(

data_scaled,

cluster_rows = TRUE,

cluster_cols = FALSE,

show_rownames = FALSE,

show_colnames = TRUE,

color = colorRampPalette(c("blue", "white", "red"))(100),

breaks = seq(-zlim, zlim, length.out = 101)

)

dev.off()

cat("Saved: ", out_file, "\n", sep = "")

}

**Upset plots in R:**

UpSetR (version 1.4.0) was used.

library(UpSetR)

## User-defined paths (edit these two variables)

input_dir <- "<PATH/TO/GENE_LISTS_DIRECTORY>"

output_dir <- "<PATH/TO/OUTPUT_DIRECTORY>"

dir.create(output_dir, showWarnings = FALSE, recursive = TRUE)

dir.create(output_dir, showWarnings = FALSE, recursive = TRUE)

## Input: gene lists (one gene symbol per line, no header)

## File selection: all files matching 'upreg.*.txt' or 'downreg.*.txt'

direction <- "upreg" # set to "upreg" or "downreg"

files <- sort(list.files(

path = input_dir,

pattern = paste0(direction, ".*\\.txt$"),

full.names = TRUE

))

# Exclude Windows Zone.Identifier pseudo-files if present

files <- files[!grepl("Zone\\.Identifier$", files)]

if (length(files) == 0) {

stop("No files found for direction = ", direction)

}

cat("Direction: ", direction, "\n", sep = "")

cat("Input file order:\n")

print(basename(files))

## Build membership matrix

gene_sets <- lapply(files, scan, what = character(), quiet = TRUE)

set_names <- sub("\\.txt$", "", basename(files))

names(gene_sets) <- set_names

m <- fromList(gene_sets)

## Plot

custom_names <- set_names

colnames(m) <- custom_names

out_png <- file.path(output_dir, paste0("UpSet_", direction, ".png"))

png(out_png, width = 3000, height = 1500, res = 300)

upset(

m,

nsets = ncol(m),

nintersects = 2^ncol(m) - 1,

text.scale = c(0, 0, 0, 0, 0, 1),

main.bar.color = "#2b2b2b",

sets.bar.color = "#4a4a4a",

matrix.color = "#2b2b2b",

point.size = 3.5,

line.size = 0,

mainbar.y.label = "Intersection Size",

sets.x.label = "Set Size"

)

dev.off()

cat("Saved: ", out_png, "\n", sep = "")

**Evaluation of TS-RIP enrichment on exons with marked read-coverage:**

awk '($3=="exon") {print $0}' PATH/GTF_Files/gencode.v34.basic.annotation.gtf > gencode.v34.exons.gtf

#wc -l gencode.v34.exons.gtf ~700k

#uniq ~400k

awk '($3=="exon") && ($7=="+") {print $0}' gencode.v34.exons.gtf | tr -d '";' | awk '{print $1 "\t" $4 "\t" $5 "\t" $24}' | sort -k1,1 -k2,2n | uniq > gencode.v34.exons.forward.bed

awk '($3=="exon") && ($7=="-") {print $0}' gencode.v34.exons.gtf | tr -d '";' | awk '{print $1 "\t" $4 "\t" $5 "\t" $24}' | sort -k1,1 -k2,2n | uniq > gencode.v34.exons.reverse.bed

awk '($3=="exon") {print $0}' gencode.v34.exons.gtf | tr -d '";' | awk '{print $24 "\t" $7 "\t" $10 "\t" $14 "\t" $16}' | sort -k1,1 | uniq > gencode.v34.exon_annotation.csv

**Read coverage calculation using deep tools** (9)**:**

for f in ./*core*rRNAdepleted.sorted.bam

do

tsamp="$(basename $f .rRNAdepleted.sorted.bam)"

bamCoverage -b ./${tsamp}.rRNAdepleted.sorted.bam -o ${tsamp}.bin10bp.RPKM.forward.bw --binSize 10 --verbose -p 32 --filterRNAstrand forward --skipNAs --normalizeUsing RPKM

bamCoverage -b ./${tsamp}.rRNAdepleted.sorted.bam -o ${tsamp}.bin10bp.RPKM.reverse.bw --binSize 10 --verbose -p 32 --filterRNAstrand reverse --skipNAs --normalizeUsing RPKM

done

for f in $DATA/*core.bin10bp.RPKM.forward.bw

do

tsamp="$(basename $f .core.bin10bp.RPKM.forward.bw)"

bigWigAverageOverBed -bedOut=$RESULTS/$tsamp.exons.forward.raw.bed $DATA/$tsamp.core.bin10bp.RPKM.forward.bw $DATA/gencode.v34.exons.forward.noY.bed DEL.tab

bigWigAverageOverBed -bedOut=$RESULTS/$tsamp.exons.reverse.raw.bed $DATA/$tsamp.core.bin10bp.RPKM.reverse.bw $DATA/gencode.v34.exons.reverse.noY.bed DEL.tab

sort -k1,1 -k2,2n $RESULTS/$tsamp.exons.forward.raw.bed | awk '{printf "%s\t", $1; printf "%s\t", $2; printf "%s\t", $3; printf "%s\t", $4; printf "%f\n", $5}' > $RESULTS/$tsamp.exons.forward.bed

sort -k1,1 -k2,2n $RESULTS/$tsamp.exons.reverse.raw.bed | awk '{printf "%s\t", $1; printf "%s\t", $2; printf "%s\t", $3; printf "%s\t", $4; printf "%f\n", $5}' > $RESULTS/$tsamp.exons.reverse.bed

rm $RESULTS/$tsamp.exons.forward.raw.bed

rm $RESULTS/$tsamp.exons.reverse.raw.bed

awk '($5>=100) {print $0}' $RESULTS/$tsamp.exons.forward.bed > $RESULTS/$tsamp.exons.forward.100.bed

awk '($5>=100) {print $0}' $RESULTS/$tsamp.exons.reverse.bed > $RESULTS/$tsamp.exons.reverse.100.bed

done

**Enrichment on TYMS and TP53 exons:**

bigWigAverageOverBed -bedOut=NT_UGI_HCT116_rep2.TP53_exons.bed NT_UGI_HCT116_rep2.lRNA.core.bin10bp.RPKM.reverse.bw TP53_exons_clean_nonRedundant.bed del.tab

for f in ../bigwig_files_as_refs/*.TS_RIP2_vs_RIPctr.core.reverse.log2.filtered.bw

do

tsamp="$(basename $f .TS_RIP2_vs_RIPctr.core.reverse.log2.filtered.bw)"

bigWigAverageOverBed -bedOut=$tsamp.TP53_exons.bed ../bigwig_files_as_refs/$tsamp.TS_RIP2_vs_RIPctr.core.reverse.log2.filtered.bw TP53_exons_clean_nonRedundant.bed del.tab

done

awk '($8=="TYMS") {print $0}' union.exons.forward.100.annotated.bed > TYMS_exons.bed

bedtools merge -c 4 -o collapse -i TYMS_exons.bed > TYMS_exons_nonRedundant.bed

for f in ../bigwig_files_as_refs/*.TS_RIP2_vs_RIPctr.core.forward.log2.filtered.bw

do

tsamp="$(basename $f .TS_RIP2_vs_RIPctr.core.forward.log2.filtered.bw)"

bigWigAverageOverBed -bedOut=$tsamp.TYMS_exons.bed ../bigwig_files_as_refs/$tsamp.TS_RIP2_vs_RIPctr.core.forward.log2.filtered.bw TYMS_exons_nonRedundant.bed del.tab

done

awk '($8=="CDKN1A") {print $0}' union.exons.forward.100.annotated.bed > CDKN1A_exons.bed

# exons that has coverage were manually selected and merged → 3 main exons

bedtools merge -c 4 -o collapse -i CDKN1A_exons.bed > CDKN1A_exons_nonRedundant.bed

for f in ../bigwig_files_as_refs/*.TS_RIP2_vs_RIPctr.core.forward.log2.filtered.bw

do

tsamp="$(basename $f .TS_RIP2_vs_RIPctr.core.forward.log2.filtered.bw)"

bigWigAverageOverBed -bedOut=$tsamp.CDKN1A_exons.bed ../bigwig_files_as_refs/$tsamp.TS_RIP2_vs_RIPctr.core.forward.log2.filtered.bw CDKN1A_exons_nonRedundant.bed del.tab

done

**Analysis of sRNA-seq data:**

**Preprocessing** was performed using trimmomatic (10) for adapter and quality trimming, and STAR aligner (11):

java -jar ${HOME}/PATH/trimmomatic.jar SE -threads 32 -phred33 -trimlog TrimLog_$tsamp.txt $DATA/$tsamp.fq.gz $tsamp.sRNA_Trimmed.fq.gz LEADING:3 TRAILING:3 ILLUMINACLIP:adapter3.fa:2:20:7 SLIDINGWINDOW:4:12 MAXINFO:16:0.8 MINLEN:16

The reference genome was downloaded from: https://www.gencodegenes.org/human/

PATH/GenomeIndeces/GRCh38.primary_assembly/genome

#Indexing the reference genome for STAR aligner:

STAR --runThreadN 32 --runMode genomeGenerate --genomeDir \ PATH/GenomeIndeces/STAR_GRCh38primary --genomeFastaFiles \ PATH/GenomeIndeces/GRCh38.primary_assembly.genome.fa --sjdbGTFfile ./ENCFF470CZH.gtf --sjdbOverhang 49

#alignment of trimmed reads:

for f in $DATA/*.sRNA_Trimmed.fq.gz

do

tsamp="$(basename $f .sRNA_Trimmed.fq.gz)"

STAR --runThreadN 32 --genomeDir PATH/GenomeIndeces/STAR_GRCh38primary \

--readFilesIn $DATA/$tsamp.sRNA_Trimmed.fq.gz --readFilesCommand gunzip -c \

--outFileNamePrefix $RESULTS/$tsamp.sRNA.STARprim

samtools view -bh -F 256 -@ 32 $RESULTS/$tsamp.sRNA.STARprimAligned.out.sam \

-L $RESULTS/list_of_chr_STARprim.bed -o \

$RESULTS/$tsamp.sRNA.STARprimAligned.filtered256.bam

samtools sort -@ 32 $RESULTS/$tsamp.sRNA.STARprimAligned.filtered256.bam > \

$RESULTS2/$tsamp.sRNA.STARprimAligned.filtered256.sorted.bam

samtools index $RESULTS2/$tsamp.sRNA.STARprimAligned.filtered256.sorted.bam

samtools idxstats $RESULTS2/$tsamp.sRNA.STARprimAligned.filtered256.sorted.bam > \

$RESULTS3/$tsamp.sRNA.STARprimAligned.filtered256.sorted.idxstats.txt

rm $RESULTS/$tsamp.sRNA.STARprimAligned.out.sam

rm $RESULTS/$tsamp.sRNA.STARprimAligned.filtered256.bam

done

**Quantification of relative expression** was done by FeatureCounts (12) and deseq2 (13,14) using an annotation file created for matured miRNA and other sRNAs:

awk -F';' '{print $1}' hsa.matured_miRNA.gff3 | awk -F'=' '{print $1 "\t" $2}' | awk '{print $10 "\t" $1 "\t" $4 "\t" $5 "\t" $7}' > hsa.matured_miRNA.featureCounts.SAF.tsv

awk -F';' '{print $1 "\t" $6}' smallRNAs.gencode.v34.annotation.gff3 | awk -F'=' '{print $1 "\t" $2 "\t" $3}' | awk '{print $12 "\t" $1 "\t" $4 "\t" $5 "\t" $7}' > smallRNAs.gencode.v34.featureCounts.SAF.tsv

awk '{print $2 "\t" $3 "\t" $4 "\t" $5 "\t" $1}' smallRNAs.gencode.v34.featureCounts.SAF.tsv > smallRNAs.gencode.v34.featureCounts.SAF.bed

bedtools merge -d -1 -c 5,4 -o collapse,first -i smallRNAs.gencode.v34.featureCounts.SAF.bed | awk '{print $4 "\t" $1 "\t" $2 "\t" $3 "\t" $5}' > smallRNAs.gencode.v34.featureCounts.SAF.nonOverlap.tsv

cat smallRNAs.gencode.v34.featureCounts.SAF.nonOverlap.tsv hsa.matured_miRNA.featureCounts.SAF.tsv | sort -k2,2 -k3,3n > smallRNA_miRNA.featureCounts.SAF.sorted.noheader.tsv

**featureCounts:**

for f in $DATA/*UGI_HCT116_rep*sRNA.STARprimAligned.filtered256.sorted.bam

do

tsamp="$(basename $f .sRNA.STARprimAligned.filtered256.sorted.bam)"

featureCounts -s 1 --minOverlap 15 --largestOverlap --nonSplitOnly -a ${GTF} -d 15 -T 32 -o $RESULTS/${tsamp}.nonSplitOnlyReadCounts1 $DATA/${tsamp}.sRNA.STARprimAligned.filtered256.sorted.bam

done

**References**

1. Andrews S. FastQC. Babraham Bioinformatics. 2010;

2. Chen S, Zhou Y, Chen Y, Gu J. Fastp: An ultra-fast all-in-one FASTQ preprocessor. In: Bioinformatics. 2018.

3. Li H, Durbin R. Fast and accurate long-read alignment with Burrows-Wheeler transform. Bioinformatics. 2010;26(5):589–95.

4. Guo Y, Dai Y, Yu H, Zhao S, Samuels DC, Shyr Y. Improvements and impacts of GRCh38 human reference on high throughput sequencing data analysis. Genomics [Internet]. 2017 Mar 1 [cited 2019 Oct 21];109(2):83–90. Available from: https://www.sciencedirect.com/science/article/pii/S0888754317300058

5. Kim D, Langmead B, Salzberg SL. HISAT: A fast spliced aligner with low memory requirements. Nat Methods [Internet]. 2015 Mar 31 [cited 2021 Mar 1];12(4):357–60. Available from: /pmc/articles/PMC4655817/

6. Trapnell C, Williams B a, Pertea G, Mortazavi A, Kwan G, van Baren MJ, et al. How Cufflinks works. Nat Biotechnol. 2010;28(5).

7. Ghosh S, Chan CKK. Analysis of RNA-seq data using TopHat and cufflinks. In: Methods in Molecular Biology [Internet]. Humana Press Inc.; 2016 [cited 2022 Nov 24]. p. 339–61. Available from: https://pubmed.ncbi.nlm.nih.gov/26519415/

8. Frankish A, Carbonell-Sala S, Diekhans M, Jungreis I, Loveland JE, Mudge JM, et al. GENCODE: reference annotation for the human and mouse genomes in 2023. Nucleic Acids Res. 2023;51(D1).

9. Ramírez F, Ryan DP, Grüning B, Bhardwaj V, Kilpert F, Richter AS, et al. deepTools2: a next generation web server for deep-sequencing data analysis. Nucleic Acids Res. 2016 Jul;44(W1):W160-5.

10. Bolger AM, Lohse M, Usadel B. Trimmomatic: a flexible trimmer for Illumina sequence data. Bioinformatics [Internet]. 2014 Aug 1 [cited 2019 Oct 22];30(15):2114–20. Available from: http://www.ncbi.nlm.nih.gov/pubmed/24695404

11. Dobin A, Davis CA, Schlesinger F, Drenkow J, Zaleski C, Jha S, et al. STAR: Ultrafast universal RNA-seq aligner. Bioinformatics. 2013;29(1).

12. Liao Y, Smyth GK, Shi W. FeatureCounts: An efficient general purpose program for assigning sequence reads to genomic features. Bioinformatics. 2014;30(7).

13. Love MI, Huber W, Anders S. Analyzing RNA-seq data with DESeq2. Bioconductor. 2017;

14. Liu S, Wang Z, Zhu R, Wang F, Cheng Y, Liu Y. Three differential expression analysis methods for rna sequencing: Limma, edger, deseq2. Journal of Visualized Experiments. 2021;2021(175).
