# Supplementary material for: Thymidylate synthase inhibitory drugs induce p53-dependent pathways differently
Source: PLoS One. 2026 Jul 1;21(7):e0332491. doi: 10.1371/journal.pone.0332491 (PMC13322534; doi:10.1371/journal.pone.0332491)
Supplement: Attachment — Submitted filename: Rebuttal_Letter20260404.docx [file pone.0332491.s016.docx]

**Rebuttal Letter**
**Manuscript ID:** PONE-D-25-47401
**Title:** *Thymidylate synthase inhibitory drugs induce p53-dependent pathways differently*
**Journal:** PLOS ONE
**Authors:** Eszter Holub^1,2,3^, Milda Blanka Szajkó^1,2^, Anna Felföldi^1^, Beáta G. Vértessy^1,2^*, Angéla Békési ^1,2^*

We thank the Editor and Reviewers for their careful evaluation of our manuscript. We greatly appreciate the constructive feedback, which has helped us improve the clarity, rigor, and presentation of our study. Below, we provide a point-by-point response to all comments. All changes made in the revised manuscript are highlighted in track changes.

# Responses to the Editor’s comments:

1. Our manuscript now meets PLOS ONE's style requirements, as defined in the PLOS ONE style templates. The changes to the style were made outside of track changes mode to avoid unnecessary confusion. We also put the “Materials and Methods” section at the end of the manuscript, right after the “Conclusions”, which is not specifically indicated in the revised manuscript with track changes.
2. We updated the grant information in the ‘Funding Information’ and ‘Financial Disclosure’ sections, which are matched now, and the updated statement is also included in the Cover Letter.
3. The data availability statement is also updated now. We have uploaded the RNA-seq data to the GEO database under accession number **GSE318306** and the TS-RIP-seq data under the accession number **GSE307531**. These are in private status until the date of acceptance or publication of this manuscript. However, Reviewers and the Editor can have access using the Private tokens ‘**mpqhqagytbcjbaf**’ and ‘**ehsjyeuytduznen**’, respectively. We have updated these accession numbers in the manuscript Data availability session and also in the corresponding “Materials and Methods” section.
4. A full ethics statement was included in the “Materials and Methods” section; however, our experimental setup did not involve human samples except the commercially available cell lines, with no restrictions for usage.
5. Figure 4 in our original submission, that is Figure 6 in the revised version, contains a scheme created in BioRender. The publication license, under the CC BY 4.0 scheme, is uploaded as an "Other" file with the submission of the revised manuscript, and the proper citation has been inserted into the figure legend.
6. The Supporting Information files with DIFF extensions are the output files of the Cuffdiff package, which are in tab-separated text format. Now, we have added appropriate extensions to the filenames, making them openable to Windows users as well. We have uploaded these files to GEO with appropriate descriptions; we did not include them as source data files with the Revised Manuscript.
7. Captions for the Supporting Information files have been inserted at the end of the manuscript, including titles for each file, and all in-text citations were updated accordingly.
8. Reviewer 1 has suggested citing several papers with the DOIs mentioned. Among these, we found only one that can somehow be related to our topic. We cited this one (DOI: 10.1371/journal.pone.0193810), but not the others (DOI: 10.2174/0113816128277350231219062154, DOI: 10.1080/03007995.2024.2416985, DOI: 10.1002/iub.1510, DOI: 10.1016/j.bioorg.2023.106427, and DOI: 10.3390/ijms25073904), with which we did not find any connections to our study.

# Responses to Reviewers' comments:

## Responses to Reviewer 1:

Thanks to Reviewer 1 for his/her positive and supportive evaluation of our manuscript. We agree that the original manuscript could be improved grammatically to make it clearer and more fluent. Hence, we corrected the text grammatically and stylistically throughout the manuscript, also using the support of Grammarly; the changes can be followed in the revised manuscript with track changes.

- We also restructured the Abstract as Reviewer 1 suggested.
- *“But the manuscript is showing details, but needs more clarification,”* Although Reviewer 1 did not specify which details need more clarification, we tried to improve the manuscript in this aspect as well. We hope that the revised version now meets Reviewer 1's expectations.
- Reviewer 1 also missed citations, especially from the Introduction: *“The introduction background is all about comprehensive summary of info, each sentence should have a ref.”* We carefully revised the Introduction and Discussion, and inserted citations where relevant. These are highlighted in yellow in the References and also in the text within the revised manuscript with track changes. Finally, 23 and 48 additional references were cited in the Introduction and the Discussion, respectively.
- Reviewer 1 specifically asked for citing the paper *(DOI: 10.1371/journal.pone.0193810)* after the first sentence of the Introduction. We appreciate the suggestion, and this paper provides valuable information about the active and inactive conformations of thymidylate synthase (TS) and their selective targeting. However, we think the paper does not support the general statement of the 1^st^ sentence: “Thymidylate synthase (TS) is one of the key enzymes of thymidine biosynthesis”, which is rather textbook information that does not require additional citation of recent papers. Instead, we cited two reviews in row 41 supporting the relevance of TS and thymidylate biosynthesis as anticancer targets. The review that was suggested by Reviewer 1 has also been cited after a new sentence in row 59 in the revised manuscript with track changes: *“This equilibrium is considered a pivotal factor in drug sensitivity; accordingly, a new group of TS inhibitors has also been designed, shifting this equilibrium towards the inactive conformation [17].”*
- Reviewer 1 specifically asked for citing the papers *(DOI: 10.2174/0113816128277350231219062154 and DOI: 10.1080/03007995.2024.2416985)* into the sentence *“Long non-coding RNAs (lncRNAs), in contrast, are longer transcripts that do not encode proteins but regulate gene expression at multiple levels: transcriptionally, by modulating chromatin structure, and posttranscriptionally, often by sponging miRNAs to prevent degradation of their target mRNAs.”* in rows 76-78 (that are 97-100 in the cleaned revised version). We found that these two papers (one is about Medulloblastoma, the other is about clinical therapies) were not relevant; however, we cited additional references supporting the functions of these non-coding RNA species: ref36 for snoRNAs, refs 37-38 for miRNAs, refs 39-41 for lncRNAs, and refs 42-43 for their role in phase separation.
- Reviewer 1 mentioned that *“The aim is well defined, but needs better objectives for clarification”.* To clarify our objectives better, we first moved the Materials and Methods section to the end of the manuscript to avoid interrupting the logic and flow. Second, we have presented and cited our previous results (published meanwhile in LSA (DOI: 10.26508/lsa.202503352)) about drug-specific differences in genome-wide uracil patterns, cell viability, and induced mutagenicity. In the present manuscript, we aimed to understand the molecular background of these differences, which now is more explicitly stated in the last paragraph of the revised Introduction.
- As Reviewer 1 suggested, we added a subheading for Bioinformatics: we modified the original *“RNA-seq analysis”* to *“Bioinformatics – RNA-seq data analysis”* in row 924.
- The resolution of the figures has also been improved. We also enlarged the Volcano plots that were too small in the original manuscript figures. We note that due to the implementation of additional RNA-seq data, most figures had to be completely revised: for multiple comparisons (cf. Fig 1 and 2), we also had to use new visualization methods (PCA plots, heatmaps, and UpSet plots).
- Reviewer 1 suggested: *“add the network and protein-protein interaction”.* We have analyzed protein-protein interaction networks and functional enrichment for different groups of differentially expressed protein-coding genes in the STRING database and provided network statistics and permanent links for those in Table 1. However, we did not provide the network figures themselves as manuscript figures. In the revised version, we have performed further analysis for the additional two cell lines (c.f. revised version of Table 1). In addition, we have implemented two networks: (1) about commonly induced genes (S2A Fig) and (2) about genes showing 5FdUR-biased expression in all cell lines (Fig 4A). The other potentially interesting networks are available via STRING permanent links (cf. Table 1).
- Reviewer 1’s comment *“the “strength(s)” of the study to be mentioned and limitations as separate subheading”* was considered. Originally, we did not prepare a Conclusion section, but in the Revised Manuscript, we included it, summarizing the strengths and limitations of the study, as Reviewer 1 had suggested.
- Reviewer 1 has suggested implementing specified references to the Discussion and an additional sentence as well: *“Recommendation and future perspectives need to be stated and highlighted, this sentence as a sustainability part “repurposing drugs as GLP-1 based therapy or targeting 20S proteasomes and giving various natural compounds as hinokitiol as prophylactic with immuno-modulatory effect with positive impact on better to be used with TS” and cite these references DOI: 10.1002/iub.1510, DOI: 10.1016/j.bioorg.2023.106427, and DOI: 10.3390/ijms25073904, respectively.”* Neither of these papers mentioned TS, the applied cell lines, or the two drugs; we did not find them appropriate to cite in our study. Instead, we have inserted relevant reviews about those regulatory procedures we have discussed. These are highlighted in References and in the text of the Revised Manuscript with track changes. We are convinced that all of them are relevant and strengthen our study. Therefore, we appreciate that Reviewer 1 drew our attention to the lack of relevant references as a major issue.
- Reviewer 1 also suggested providing a List of abbreviations that we prepared and attached as supporting information, S1 Table.

## Responses to Reviewer 2

### Reviewer 2’s general evaluation:

*The study identifies correlations between drug treatments (5FdUR and RTX) and transcriptomic differences, but does not really establish mechanisms linking Tymidylate synthase-RNA interactions to differential p53 pathway activation. The authors infer that altered TS–RNA binding explains p53/p21 expression discrepancies, but no conclusive functional assays (e.g., RNA-binding mutants of TS, RNA pulldown validation, or reporter assays) are provided to test this directly. The conclusion that TS acts as a major post-transcriptional regulator influencing p53 signaling is not really supported. Differences between RTX and 5FdUR responses are presented as biologically distinct, but the data does not support this at this stage.*

*The data set is of interest and may provide useful information about the drugs and p53 response, but for this to be, it is necessary to provide more information on p53 and p21 at various concentrations, time points, and also in at least one more cell lines. Otherwise it is very difficult to generalize on these findings.*

We thank Reviewer 2 for the constructive and thoughtful evaluation of our manuscript. We agree that the original manuscript could be strengthened from a statistical point of view by extending the analysis to other cell lines and by addressing the time- and dose-dependency. We have addressed these points in the revised manuscript. We also appreciate the comment regarding data availability; all corresponding sequencing data have now been deposited in the GEO database.

Regarding the concern about our main conclusions, we would like to clarify that the primary focus of our study is demonstrating drug-specific differences, with particular emphasis on the 5FdUR-biased effects on p53-related pathways. The role of TS as a post-transcriptional regulator of p53 and p21 was intended to present as a complementary, exploratory aspect rather than a central mechanistic claim. In the Revised Manuscript, this section has been further de-emphasized, especially in light of additional RNA-seq data, and the corresponding statements have been revised to adopt a more cautious interpretation.

In addition, our related study, which has since been published (DOI: 10.26508/LSA.202503352), provides further context for the observed drug-specific differences. In that work, we demonstrate alterations between these two TS-inhibitory treatments in terms of drug sensitivity, genomic uracilation profiles, and drug-induced mutagenesis in the same cell line model. Notably, that study addresses the time- and dose-dependency at the level of cell viability, revealing reduced sensitivity specifically in response to high-dose 5FdUR treatment, which is also associated with increased cytosine deamination events in genomic DNA. The interpretation of the present transcriptomic findings is now better supported in the context of these previously established drug-specific differences.

In agreement with Reviewer’s suggestions and to strengthen the current study, we have expanded the experimental scope as follows: (1) inclusion of transcriptome analyses from two additional HCT116-derived cell lines with distinct DNA repair capacities; (2) extension of RT–qPCR validation and (3) Western blot analyses to additional drug concentrations.

Taken together, these additions further support our main conclusion that high-dose 5FdUR induces a stronger activation of p53-related pathways compared to RTX. At the same time, we acknowledge that the RNA-binding and regulatory role of TS has not been further investigated. To avoid overinterpretation of this part, we have revised the manuscript to present this aspect more cautiously as a potential, rather than a definite mechanism.

### Major and minor points of Reviewer 2

***Point 1:***

*Most of the data is done in MMR-deficient HCT116 model, if I see correct there is no comparison to wild-type HCT116 or other cell lines, making it rather difficult to generalize findings.*

**Response 1:**

We thank Reviewer 2 for this valuable comment. In the revised manuscript, we have expanded our analysis by including additional transcriptome sequencing data from wild-type HCT116 cells, as well as from MMR-proficient but UNG-inhibited HCT116 cells (HCT116-MMR-UGI). Together, these three cell lines represent distinct uracil-DNA repair capacities, enabling a more comprehensive and generalizable assessment of drug-induced transcriptomic changes. The inclusion of these new sequencing datasets, comprising both RTX- or 5FdUR-treated samples alongside their respective non-treated controls, required reanalysis of the entire dataset and the application of updated visualization approaches. As a result, the revised manuscript includes substantial modifications, including new figures and expanded subsections in both the Results and Discussion.

***Point 2.***

*It also looks as if there is no proper vehicle control (solvent only), data are shown to confirm that observed effects are due to the drugs themselves and not general stress. How was the compounds dissolved and given to the cells? This may just need a clarification though.*

**Response 2:**

We thank the Reviewer for this important comment. As the two drugs were dissolved in different solvents (RTX in DMSO, and 5FdUR in water), we have now included an additional DMSO-only control in our Western blot analysis. This control confirmed that the DSMO-treated sample behaved similarly to the non-treated control (c.f. revised Fig 5D and S6 Fig), indicating that the observed effects are attributable to the drugs rather than solvent-induced stress. The corresponding figure legends and methods section have also been revised accordingly.

***Point 3.***

*Most of the experiments rely on single concentration point, this is very risky, for example p53-p21 response could change a lot depending on time and concentration. It would be advised to do dose response curves and also check how p53 and p21 behaves at various concentrations. This is the biggest issue for this referee.*

**Response 3:**

We thank the Reviewer for highlighting the importance of dose- and time-dependent effects, which we agree is a critical aspect of interpreting the cellular responses. We have now directly addressed this point by extending our experimental analysis to include additional drug concentrations.

Guided by our recently published study (DOI: 10.26508/LSA.202503352), in which dose- and time-dependent effects on cell viability were systematically characterized, we selected additional concentration points to evaluate transcriptional and protein-level responses. Specifically, we performed additional RT-qPCR measurements of MDM2, CDKN1A/p21, and CCNG1/cycling following 48 h treatments of HCT116-UGI cells with both low (100 nM) and high (20 µM) concentrations of RTX and 5FdUR (cf. revised Fig 5C). In parallel, we expanded our Western blot analysis to include the same drug concentrations, enabling comparison of γH2AX, TS, p53, and p21 protein levels across a broader concentration range (cf. Fig 5D and 6S Fig).

These new data demonstrate clear dose-dependent differences. Interestingly, high-dose RTX did not further enhance p21 expression at either the mRNA or protein level; instead, a reduction was observed compared to the lower dose. (Notably, this trend is consistent with the induction patterns of APOBEC enzymes reported in our published study (DOI: 10.26508/LSA.202503352)). In contrast, low-dose 5FdUR elicited responses comparable to those seen with high-dose RTX, while the strongest overall effects (though not necessarily the biggest drug-specific differences) were consistently observed at 0.1 µM RTX and 20 µM 5FdUR. Importantly, these are the same concentrations used in our previous U-DNA-Seq and the recent RNA-seq experiments, supporting the relevance of our transcriptomic analyses. Furthermore, a similar induction of DNA damage marker γH2AX in the insoluble fraction was observed across all tested concentrations, indicating that activation of the DNA damage response is robust and not dose-dependent in this range.

Taken together, these additional experiments directly address the Reviewer’s concern and demonstrate that our conclusions are supported across the tested concentration range, further supporting the robustness of the observed p53 pathway activation differences between RTX and 5FdUR.

***Point 4.***

*Figures and supplementary data are not clearly summarized in the text.*

**Response 4:**

We thank the Reviewer for this comment. In the revised manuscript, we have improved the presentation and integration of figures and supplementary data. In accordance with PLOS ONE requirements, the titles and captions of all supplementary figures and tables are now included at the end of the manuscript document. In addition, all supplementary materials are consistently cited in the relevant sections of the text.

We have also revised the Results and Discussion to provide clearer and more detailed descriptions of the presented data, which have been markedly extended by the inclusion of additional RNA-seq experiments. To better reflect these expanded analyses, the figure set has been comprehensively reorganized. Specifically, the original Fig 1 has been replaced by Fig 1-3, and S1, S7-S10 Figs; Fig 2 has been replaced by Fig 4 and S2-S3 Figs; Fig 3 has been revised as Fig 5 and extended with S4-S6 Figs; Fig 4 has been revised as Fig 6; and S1 Fig has been revised as S11 Fig. The original Fig. S2 has been removed in the revised version.

These changes ensure clearer presentation and more appropriate comparisons across the extended transcriptomic dataset. As a result, the key findings are now more explicitly linked to the corresponding figures. Particular emphasis has been placed on Figures 3 and 4, which support the central focus of the manuscript on the 5FdUR-biased effects on p53-related pathways, while supplementary figures provide additional supporting evidence.

***Point 5.***

*Some of the plots (volcano plots, gene listings (bars) have very poor resolution also in the original TIFF format that I could access (not only in the PDF combined). It makes it impossible to read. It is necessary to improve figure 1,2,4. And re-review these.*

**Response 5:**

We thank the Reviewer for highlighting this issue. In the revised manuscript, we have improved the resolution and overall readability of all figures, including the volcano plots and enrichment graphs.

Specifically, we increased figure dimensions and resolution, optimized labeling to focus on the most relevant genes, and applied clearer and more consistent color schemes to enhance visual interpretation. The enrichment graphs on the original Fig 2 have been extensively revised and replotted (cf. Fig 4 and S2, S3 Figs). In addition, we used the recommended PACE tool to ensure that all figures meet the journal’s formatting and quality requirements. These changes ensure that all elements are legible now.

***Point 6.***

*The seq data accessibility is listed as “GEO accession numbers in progress,” which I m o does not meet PLOS ONE’s open data policy at submission. It is necessary to access.*

**Response 6:**

We thank the Reviewer for highlighting this crucial issue. We have now corrected this mistake by depositing both the RNA-seq and TS-RIP-seq datasets in the GEO database under accession numbers GSE318306 and GSE307531, respectively. The accession numbers and access details have been updated in the Data Availability statement and in the relevant sections of the Materials and Methods.

**Point 7.**

The abbreviation TS is a bit confusing, it sounds on some places like authors invented novel technology TS-RIP or TS-RNA and so on. Better to spell the name out.

**Response 7:**

We thank the Reviewer for this helpful suggestion. To improve clarity, we have revised the manuscript to explicitly define thymidylate synthase (TS) on the first occurrence and to ensure consistent usage throughout the text. While we agree that excessive abbreviations can be confusing, we have retained “TS” after its initial definition for readability and consistency, as it is commonly used in the field.

To avoid any potential confusion, particularly regarding the abbreviations TS-RIP and TS-RIP-seq, we clarified that RIP and RIP-seq are standard methods applied here using a TS-specific antibody. Accordingly, we revised the Methods section subtitles to “RNA co-immunoprecipitation via thymidylate synthase (TS-RIP)” and to “Sequencing of TS-RIP samples and data analysis”. We also adjusted the corresponding sentences in the Abstract and at the end of the Introduction to clarify usage (“…co-immunoprecipitation coupled to sequencing revealed direct RNA partners of TS…“ and “…using a TS-coupled RNA-co-immunoprecipitation (TS-RIP) followed by sequencing (abbreviated as TS-RIP-seq) ...”). Additional minor refinements have been implemented throughout the manuscript (see track changes).

**Point 8.**

If possible provide replicate validation of RNA binding

**Response 8:**

We thank the Reviewer for this suggestion. Due to financial and logistical limitations, we were unable to perform replicate RNA-IP-seq experiments in this study. Importantly, the RNA co-immunoprecipitation analysis was intended as an exploratory, screening-type experiment to identify potential TS-associated RNAs rather than to provide definitive mechanistic validation.

We have revised the manuscript to clarify this point and have emphasized that further functional validation of TS–RNA interactions remains beyond the scope of the current study.

**Point 9.**

Deposit sequencing data with accession numbers before resubmission. Reviewers can get a token (secure) before, so it is not open for anyone to download. Is very easy.

**Response 8:**

We thank the Reviewer for highlighting this issue. We have now deposited both the RNA-seq and TS-RIP-seq datasets in the GEO database under accession numbers GSE318306 and GSE307531, respectively. These datasets are currently in private status and are accessible to the Reviewers using the provided access tokens ‘mpqhqagytbcjbaf’ and ‘ehsjyeuytduznen’, respectively.

We sincerely thank the Editor and Reviewers for their thoughtful comments and careful consideration of our manuscript. We hope that the revisions and clarifications provided here address all concerns and improve the quality and clarity of our work.

Sincerely,
Angela Bekesi on behalf of all co-authors
