# Supplementary material for: Thymidylate synthase inhibitory drugs induce p53-dependent pathways differently
Source: PLoS One. 2026 Jul 1;21(7):e0332491. doi: 10.1371/journal.pone.0332491 (PMC13322534; doi:10.1371/journal.pone.0332491)
Supplement: Peer review history

# Peer review: Decision Letter 0

**DOI:** 10.1371/journal.pone.0332491.r001

**Authors:** Chien-Feng Li

22 Oct 2025

PONE-D-25-47401

Thymidylate synthase inhibitory drugs induce p53-dependent pathways differently

PLOS ONE

Dear Dr. Békési,

Thank you for submitting your manuscript to PLOS ONE. After careful consideration, we feel that it has merit but does not fully meet PLOS ONE’s publication criteria as it currently stands. Therefore, we invite you to submit a revised version of the manuscript that addresses the points raised during the review process.

==============================

**ACADEMIC EDITOR:**

I would like to invite you to revise your work as per the reviewers' comments.

==============================

Please submit your revised manuscript by Dec 06 2025 11:59PM. If you will need more time than this to complete your revisions, please reply to this message or contact the journal office at plosone@plos.org. When you're ready to submit your revision, log on to https://www.editorialmanager.com/pone/ and select the 'Submissions Needing Revision' folder to locate your manuscript file.

Please include the following items when submitting your revised manuscript:

- A rebuttal letter that responds to each point raised by the academic editor and reviewer(s). You should upload this letter as a separate file labeled 'Response to Reviewers'.
- A marked-up copy of your manuscript that highlights changes made to the original version. You should upload this as a separate file labeled 'Revised Manuscript with Track Changes'.
- An unmarked version of your revised paper without tracked changes. You should upload this as a separate file labeled 'Manuscript'.

If you would like to make changes to your financial disclosure, please include your updated statement in your cover letter. Guidelines for resubmitting your figure files are available below the reviewer comments at the end of this letter.

If applicable, we recommend that you deposit your laboratory protocols in protocols.io to enhance the reproducibility of your results. Protocols.io assigns your protocol its own identifier (DOI) so that it can be cited independently in the future. For instructions see: https://journals.plos.org/plosone/s/submission-guidelines#loc-laboratory-protocols. Additionally, PLOS ONE offers an option for publishing peer-reviewed Lab Protocol articles, which describe protocols hosted on protocols.io. Read more information on sharing protocols at https://plos.org/protocols?utm_medium=editorial-email&utm_source=authorletters&utm_campaign=protocols.

We look forward to receiving your revised manuscript.

Kind regards,

Chien-Feng Li, M.D., Ph.D.

Academic Editor

PLOS ONE

Journal Requirements:

When submitting your revision, we need you to address these additional requirements.

1. Please ensure that your manuscript meets PLOS ONE's style requirements, including those for file naming. The PLOS ONE style templates can be found at

https://journals.plos.org/plosone/s/file?id=wjVg/PLOSOne_formatting_sample_main_body.pdf and

https://journals.plos.org/plosone/s/file?id=ba62/PLOSOne_formatting_sample_title_authors_affiliations.pdf

2. We note that the grant information you provided in the ‘Funding Information’ and ‘Financial Disclosure’ sections do not match.

When you resubmit, please ensure that you provide the correct grant numbers for the awards you received for your study in the ‘Funding Information’ section.

3. Thank you for stating in your Funding Statement:

“Project no. 137867 has been implemented with the support provided by the Ministry of Innovation and Technology of Hungary from the National Research, Development and Innovation Fund, financed under the OTKA_FK_21 funding scheme for A.B., who was also supported by the János Bolyai Research Scholarship of the Hungarian Academy of Sciences (BO/726/22/8) and by the ÚNKP-22-5 New National Excellence Program of the Ministry of Culture and Innovation from the source of the National Research, Development and Innovation Fund. For E.H.: The scientific work and results publicized in this article was reached with the sponsorship of Gedeon Richter Talentum Foundation in the framework of Gedeon Richter Excellence PhD Scholarship of Gedeon Richter Plc. E.H. was also supported by the EKÖP-24 University Excellence Scholarship Program (EKÖP-24-3-II-ELTE-596) of the Ministry for Culture and Innovation from the source of the National Research, Development and Innovation Fund. B.G.V. was supported by the National Research, Development and Innovation Fund of Hungary grants K146890, K135231, FK137867, 2018-1.2.1-NKP-2018-00005, 2022-1.2.2-TÉT-IPARI-UZ-2022-00003, and the TKP2021-EGA-02.”

Please provide an amended statement that declares *all* the funding or sources of support (whether external or internal to your organization) received during this study, as detailed online in our guide for authors at http://journals.plos.org/plosone/s/submit-now. Please also include the statement “There was no additional external funding received for this study.” in your updated Funding Statement.

Please include your amended Funding Statement within your cover letter. We will change the online submission form on your behalf.

4. When completing the data availability statement of the submission form, you indicated that you will make your data available on acceptance. We strongly recommend all authors decide on a data sharing plan before acceptance, as the process can be lengthy and hold up publication timelines. Please note that, though access restrictions are acceptable now, your entire data will need to be made freely accessible if your manuscript is accepted for publication. This policy applies to all data except where public deposition would breach compliance with the protocol approved by your research ethics board. If you are unable to adhere to our open data policy, please kindly revise your statement to explain your reasoning and we will seek the editor's input on an exemption. Please be assured that, once you have provided your new statement, the assessment of your exemption will not hold up the peer review process.

5. Please note that your Data Availability Statement is currently missing the DOI/accession number of each dataset OR a direct link to access each database. If your manuscript is accepted for publication, you will be asked to provide these details on a very short timeline. We therefore suggest that you provide this information now, though we will not hold up the peer review process if you are unable.

6. Please include your full ethics statement in the ‘Methods’ section of your manuscript file. In your statement, please include the full name of the IRB or ethics committee who approved or waived your study, as well as whether or not you obtained informed written or verbal consent. If consent was waived for your study, please include this information in your statement as well.

7. We note that Figure 4 in your submission contain copyrighted images. All PLOS content is published under the Creative Commons Attribution License (CC BY 4.0), which means that the manuscript, images, and Supporting Information files will be freely available online, and any third party is permitted to access, download, copy, distribute, and use these materials in any way, even commercially, with proper attribution. For more information, see our copyright guidelines: http://journals.plos.org/plosone/s/licenses-and-copyright.

We require you to either (1) present written permission from the copyright holder to publish these figures specifically under the CC BY 4.0 license, or (2) remove the figures from your submission:

a. You may seek permission from the original copyright holder of Figure 4 to publish the content specifically under the CC BY 4.0 license.

We recommend that you contact the original copyright holder with the Content Permission Form (http://journals.plos.org/plosone/s/file?id=7c09/content-permission-form.pdf) and the following text:

“I request permission for the open-access journal PLOS ONE to publish XXX under the Creative Commons Attribution License (CCAL) CC BY 4.0 (http://creativecommons.org/licenses/by/4.0/). Please be aware that this license allows unrestricted use and distribution, even commercially, by third parties. Please reply and provide explicit written permission to publish XXX under a CC BY license and complete the attached form.”

Please upload the completed Content Permission Form or other proof of granted permissions as an "Other" file with your submission.

In the figure caption of the copyrighted figure, please include the following text: “Reprinted from [ref] under a CC BY license, with permission from [name of publisher], original copyright [original copyright year].”

b. If you are unable to obtain permission from the original copyright holder to publish these figures under the CC BY 4.0 license or if the copyright holder’s requirements are incompatible with the CC BY 4.0 license, please either i) remove the figure or ii) supply a replacement figure that complies with the CC BY 4.0 license. Please check copyright information on all replacement figures and update the figure caption with source information. If applicable, please specify in the figure caption text when a figure is similar but not identical to the original image and is therefore for illustrative purposes only.

8. We are unable to open your Supporting Information files 5FdUR_vs_NT.cuffdiff_DE_lRNA.cuffdiff.gene_exp.GeneCodeV34.diff, 5FdUR_vs_RTX.cuffdiff_DE_lRNA.cuffdiff.gene_exp.GeneCodeV34.diff, RTX_vs_NT.cuffdiff_DE_lRNA.cuffdiff.gene_exp.GeneCodeV34.diff, Please kindly revise as necessary and re-upload.

9. Please include captions for your Supporting Information files at the end of your manuscript, and update any in-text citations to match accordingly. Please see our Supporting Information guidelines for more information: http://journals.plos.org/plosone/s/supporting-information.

10. If the reviewer comments include a recommendation to cite specific previously published works, please review and evaluate these publications to determine whether they are relevant and should be cited. There is no requirement to cite these works unless the editor has indicated otherwise.

[Note: HTML markup is below. Please do not edit.]

Reviewers' comments:

Reviewer's Responses to Questions

**Comments to the Author**

1. Is the manuscript technically sound, and do the data support the conclusions?

The manuscript must describe a technically sound piece of scientific research with data that supports the conclusions. Experiments must have been conducted rigorously, with appropriate controls, replication, and sample sizes. The conclusions must be drawn appropriately based on the data presented.

Reviewer #1: Yes

Reviewer #2: Partly

**********

2. Has the statistical analysis been performed appropriately and rigorously?

Reviewer #1: Yes

Reviewer #2: No

**********

3. Have the authors made all data underlying the findings in their manuscript fully available?

The PLOS Data policy requires authors to make all data underlying the findings described in their manuscript fully available without restriction, with rare exception (please refer to the Data Availability Statement in the manuscript PDF file). The data should be provided as part of the manuscript or its supporting information, or deposited to a public repository. For example, in addition to summary statistics, the data points behind means, medians and variance measures should be available. If there are restrictions on publicly sharing data—e.g. participant privacy or use of data from a third party—those must be specified.

Reviewer #1: Yes

Reviewer #2: No

**********

4. Is the manuscript presented in an intelligible fashion and written in standard English?

PLOS ONE does not copyedit accepted manuscripts, so the language in submitted articles must be clear, correct, and unambiguous. Any typographical or grammatical errors should be corrected at revision, so please note any specific errors here.

Reviewer #1: No

Reviewer #2: Yes

**********

5. Review Comments to the Author

Please use the space provided to explain your answers to the questions above. You may also include additional comments for the author, including concerns about dual publication, research ethics, or publication ethics. (Please upload your review as an attachment if it exceeds 20,000 characters)

Reviewer #1: This research article submitted to Plos One, titled “Thymidylate synthase inhibitory drugs induce p53-dependent pathways differently” by Holub et al., 2025

• The title reflects the idea, disease, and the mechanism

• Abstract appropriately summarize the manuscript, better to be structured

• keywords: ok,

• The manuscript idea is interesting, imp.

• But the manuscript is showing details, but needs more clarification,

• Throughout the manuscript introduction several sentences with no ref.

• The introduction background is all about comprehensive summary of info, each sentence should have a ref.

• Start the manuscript with the subheading background,

• The introduction to mention “Thymidylate synthase (TS) is one of the key enzymes of thymidine biosynthesis.” please cite this reference DOI: 10.1371/journal.pone.0193810

• To the sentence from lines 76-78 is long with no references “Long non-coding RNAs (lncRNAs), in contrast, are longer transcripts that do not encode proteins but regulate gene expression at multiple levels: transcriptionally, by modulating chromatin structure, and posttranscriptionally, often by sponging miRNAs to prevent degradation of their target mRNAs.” add also to this info cite these references DOI: 10.2174/0113816128277350231219062154 and DOI: 10.1080/03007995.2024.2416985

• Again, several sentences without ref.

• The aim is well defined, but needs better objectives for clarification

• Methods part is ok and comprehensive

• Add a subheading for bioinformatics

• Statistical analysis is correct

• Results is with informative subheadings

• Figures and tables are appropriate but figures resolution needs to be better

• Add the network and protein-protein interaction

• the discussion part is adequate and well written, however; but needs more citation

• the “strength(s)” of the study to be mentioned and limitations as separate subheading

• Recommendation and future perspectives need to be stated and highlighted,

this sentence as a sustainability part “repurposing drugs as GLP-1 based therapy or targeting 20S proteasomes and giving various natural compounds as hinokitiol as prophylactic with immuno-modulatory effect with positive impact on better to be used with TS” and cite these references DOI: 10.1002/iub.1510, DOI: 10.1016/j.bioorg.2023.106427, and DOI: 10.3390/ijms25073904, respectively.

• References by authors are few and need to add all the recommended citations as well as more references

• List of abbreviations to be provided,

• The final recommendation Accept after Minor Revision.

Reviewer #2: The study identifies correlations between drug treatments (5FdUR and RTX) and transcriptomic differences, but does not really establish mechanisms linking Tymidylate synthase-RNA interactions to differential p53 pathway activation. The authors infer that altered TS–RNA binding explains p53/p21 expression discrepancies, but no conclusive functional assays (e.g., RNA-binding mutants of TS, RNA pulldown validation, or reporter assays) are provided to test this directly. The conclusion that TS acts as a major post-transcriptional regulator influencing p53 signaling is not really supported. Differences between RTX and 5FdUR responses are presented as biologically distinct, but the data does not support this at this stage.

The data set is of interest and may provide useful information about the drugs and p53 response, but for this to be, it is necessary to provide more information on p53 and p21 at various concentrations, time points, and also in at least one more cell lines. Otherwise it is very difficult to generalize on these findings.

Major and minor points

1. Most of the data is done in MMR-deficient HCT116 model, if I see correct there is no comparison to wild-type HCT116 or other cell lines, making it rather difficult to generalize findings.

2. It also looks as if there is no proper vehicle control (solvent only), data are shown to confirm that observed effects are due to the drugs themselves and not general stress. How was the compounds dissolved and given to the cells? This may just need a clarification though.

3. Most of the experiments rely on single concentration point, this is very risky, for example p53-p21 response could change a lot depending on time and concentration. It would be advised to do dose response curves and also check how p53 and p21 behaves at various concentrations. This is the biggest issue for this referee.

4. Figures and supplementary data are not clearly summarized in the text.

5. Some of the plots (volcano plots, gene listings (bars) have very poor resolution also in the original TIFF format that I could access (not only in the PDF combined). It makes it impossible to read. It is necessary to improve figure 1,2,4. And re-review these.

6. The seq data accessibility is listed as “GEO accession numbers in progress,” which I m o does not meet PLOS ONE’s open data policy at submission. It is necessary to access.

7. The abbreviation TS is a bit confusing, it sounds on some places like authors invented novel technology TS-RIP or TS-RNA and so on. Better to spell the name out.

8. If possible provide replicate validation of RNA binding

9. Deposit sequencing data with accession numbers before resubmission. Reviewers can get a token (secure) before, so it is not open for anyone to download. Is very easy.

**********

6. PLOS authors have the option to publish the peer review history of their article (what does this mean?). If published, this will include your full peer review and any attached files.

If you choose “no”, your identity will remain anonymous but your review may still be made public.

**Do you want your identity to be public for this peer review?**  For information about this choice, including consent withdrawal, please see our Privacy Policy.

Reviewer #1: No

Reviewer #2: No

**********

[NOTE: If reviewer comments were submitted as an attachment file, they will be attached to this email and accessible via the submission site. Please log into your account, locate the manuscript record, and check for the action link "View Attachments". If this link does not appear, there are no attachment files.]

To ensure your figures meet our technical requirements, please review our figure guidelines: https://journals.plos.org/plosone/s/figures

You may also use PLOS’s free figure tool, NAAS, to help you prepare publication quality figures: https://journals.plos.org/plosone/s/figures#loc-tools-for-figure-preparation.

NAAS will assess whether your figures meet our technical requirements by comparing each figure against our figure specifications.

# Author comment: Author response to Decision Letter 1

**DOI:** 10.1371/journal.pone.0332491.r002

10 Apr 2026

Responses to the Editor’s comments:

1. Our manuscript now meets PLOS ONE's style requirements, as defined in the PLOS ONE style templates. The changes to the style were made outside of track changes mode to avoid unnecessary confusion. We also put the “Materials and Methods” section at the end of the manuscript, right after the “Conclusions”, which is not specifically indicated in the revised manuscript with track changes.

2. We updated the grant information in the ‘Funding Information’ and ‘Financial Disclosure’ sections, which are matched now, and the updated statement is also included in the Cover Letter.

3. The data availability statement is also updated now. We have uploaded the RNA-seq data to the GEO database under accession number GSE318306 and the TS-RIP-seq data under the accession number GSE307531. These are in private status until the date of acceptance or publication of this manuscript. However, Reviewers and the Editor can have access using the Private tokens ‘mpqhqagytbcjbaf’ and ‘ehsjyeuytduznen’, respectively. We have updated these accession numbers in the manuscript Data availability session and also in the corresponding “Materials and Methods” section.

4. A full ethics statement was included in the “Materials and Methods” section; however, our experimental setup did not involve human samples except the commercially available cell lines, with no restrictions for usage.

5. Figure 4 in our original submission, that is Figure 6 in the revised version, contains a scheme created in BioRender. The publication license, under the CC BY 4.0 scheme, is uploaded as an "Other" file with the submission of the revised manuscript, and the proper citation has been inserted into the figure legend.

6. The Supporting Information files with DIFF extensions are the output files of the Cuffdiff package, which are in tab-separated text format. Now, we have added appropriate extensions to the filenames, making them openable to Windows users as well. We have uploaded these files to GEO with appropriate descriptions; we did not include them as source data files with the Revised Manuscript.

7. Captions for the Supporting Information files have been inserted at the end of the manuscript, including titles for each file, and all in-text citations were updated accordingly.

8. Reviewer 1 has suggested citing several papers with the DOIs mentioned. Among these, we found only one that can somehow be related to our topic. We cited this one (DOI: 10.1371/journal.pone.0193810), but not the others (DOI: 10.2174/0113816128277350231219062154, DOI: 10.1080/03007995.2024.2416985, DOI: 10.1002/iub.1510, DOI: 10.1016/j.bioorg.2023.106427, and DOI: 10.3390/ijms25073904), with which we did not find any connections to our study.

Responses to Reviewers' comments:

Responses to Reviewer 1:

Thanks to Reviewer 1 for his/her positive and supportive evaluation of our manuscript. We agree that the original manuscript could be improved grammatically to make it clearer and more fluent. Hence, we corrected the text grammatically and stylistically throughout the manuscript, also using the support of Grammarly; the changes can be followed in the revised manuscript with track changes.

• We also restructured the Abstract as Reviewer 1 suggested.

• “But the manuscript is showing details, but needs more clarification,” Although Reviewer 1 did not specify which details need more clarification, we tried to improve the manuscript in this aspect as well. We hope that the revised version now meets Reviewer 1's expectations.

• Reviewer 1 also missed citations, especially from the Introduction: “The introduction background is all about comprehensive summary of info, each sentence should have a ref.” We carefully revised the Introduction and Discussion, and inserted citations where relevant. These are highlighted in yellow in the References and also in the text within the revised manuscript with track changes. Finally, 23 and 48 additional references were cited in the Introduction and the Discussion, respectively.

• Reviewer 1 specifically asked for citing the paper (DOI: 10.1371/journal.pone.0193810) after the first sentence of the Introduction. We appreciate the suggestion, and this paper provides valuable information about the active and inactive conformations of thymidylate synthase (TS) and their selective targeting. However, we think the paper does not support the general statement of the 1st sentence: “Thymidylate synthase (TS) is one of the key enzymes of thymidine biosynthesis”, which is rather textbook information that does not require additional citation of recent papers. Instead, we cited two reviews in row 41 supporting the relevance of TS and thymidylate biosynthesis as anticancer targets. The review that was suggested by Reviewer 1 has also been cited after a new sentence in row 59 in the revised manuscript with track changes: “This equilibrium is considered a pivotal factor in drug sensitivity; accordingly, a new group of TS inhibitors has also been designed, shifting this equilibrium towards the inactive conformation [17].”

• Reviewer 1 specifically asked for citing the papers (DOI: 10.2174/0113816128277350231219062154 and DOI: 10.1080/03007995.2024.2416985) into the sentence “Long non-coding RNAs (lncRNAs), in contrast, are longer transcripts that do not encode proteins but regulate gene expression at multiple levels: transcriptionally, by modulating chromatin structure, and posttranscriptionally, often by sponging miRNAs to prevent degradation of their target mRNAs.” in rows 76-78 (that are 97-100 in the cleaned revised version). We found that these two papers (one is about Medulloblastoma, the other is about clinical therapies) were not relevant; however, we cited additional references supporting the functions of these non-coding RNA species: ref36 for snoRNAs, refs 37-38 for miRNAs, refs 39-41 for lncRNAs, and refs 42-43 for their role in phase separation.

• Reviewer 1 mentioned that “The aim is well defined, but needs better objectives for clarification”. To clarify our objectives better, we first moved the Materials and Methods section to the end of the manuscript to avoid interrupting the logic and flow. Second, we have presented and cited our previous results (published meanwhile in LSA (DOI: 10.26508/lsa.202503352)) about drug-specific differences in genome-wide uracil patterns, cell viability, and induced mutagenicity. In the present manuscript, we aimed to understand the molecular background of these differences, which now is more explicitly stated in the last paragraph of the revised Introduction.

• As Reviewer 1 suggested, we added a subheading for Bioinformatics: we modified the original “RNA-seq analysis” to “Bioinformatics – RNA-seq data analysis” in row 924.

• The resolution of the figures has also been improved. We also enlarged the Volcano plots that were too small in the original manuscript figures. We note that due to the implementation of additional RNA-seq data, most figures had to be completely revised: for multiple comparisons (cf. Fig 1 and 2), we also had to use new visualization methods (PCA plots, heatmaps, and UpSet plots).

• Reviewer 1 suggested: “add the network and protein-protein interaction”. We have analyzed protein-protein interaction networks and functional enrichment for different groups of differentially expressed protein-coding genes in the STRING database and provided network statistics and permanent links for those in Table 1. However, we did not provide the network figures themselves as manuscript figures. In the revised version, we have performed further analysis for the additional two cell lines (c.f. revised version of Table 1). In addition, we have implemented two networks: (1) about commonly induced genes (S2A Fig) and (2) about genes showing 5FdUR-biased expression in all cell lines (Fig 4A). The other potentially interesting networks are available via STRING permanent links (cf. Table 1).

• Reviewer 1’s comment “the “strength(s)” of the study to be mentioned and limitations as separate subheading” was considered. Originally, we did not prepare a Conclusion section, but in the Revised Manuscript, we included it, summarizing the strengths and limitations of the study, as Reviewer 1 had suggested.

• Reviewer 1 has suggested implementing specified references to the Discussion and an additional sentence as well: “Recommendation and future perspectives need to be stated and highlighted, this sentence as a sustainability part “repurposing drugs as GLP-1 based therapy or targeting 20S proteasomes and giving various natural compounds as hinokitiol as prophylactic with immuno-modulatory effect with positive impact on better to be used with TS” and cite these references DOI: 10.1002/iub.1510, DOI: 10.1016/j.bioorg.2023.106427, and DOI: 10.3390/ijms25073904, respectively.” Neither of these papers mentioned TS, the applied cell lines, or the two drugs; we did not find them appropriate to cite in our study. Instead, we have inserted relevant reviews about those regulatory procedures we have discussed. These are highlighted in References and in the text of the Revised Manuscript with track changes. We are convinced that all of them are relevant and strengthen our study. Therefore, we appreciate that Reviewer 1 drew our attention to the lack of relevant references as a major issue.

• Reviewer 1 also suggested providing a List of abbreviations that we prepared and attached as supporting information, S1 Table.

Responses to Reviewer 2

Reviewer 2’s general evaluation:

The study identifies correlations between drug treatments (5FdUR and RTX) and transcriptomic differences, but does not really establish mechanisms linking Tymidylate synthase-RNA interactions to differential p53 pathway activation. The authors infer that altered TS–RNA binding explains p53/p21 expression discrepancies, but no conclusive functional assays (e.g., RNA-binding mutants of TS, RNA pulldown validation, or reporter assays) are provided to test this directly. The conclusion that TS acts as a major post-transcriptional regulator influencing p53 signaling is not really supported. Differences between RTX and 5FdUR responses are presented as biologically distinct, but the data does not support this at this stage.

The data set is of interest and may provide useful information about the drugs and p53 response, but for this to be, it is necessary to provide more information on p53 and p21 at various concentrations, time points, and also in at least one more cell lines. Otherwise it is very difficult to generalize on these findings.

We thank Reviewer 2 for the constructive and thoughtful evaluation of our manuscript. We agree that the original manuscript could be strengthened from a statistical point of view by extending the analysis to other cell lines and by addressing the time- and dose-dependency. We have addressed these points in the revised manuscript. We also appreciate the comment regarding data availability; all corresponding sequencing data have now been deposited in the GEO database.

Regarding the concern about our main conclusions, we would like to clarify that the primary focus of our study is demonstrating drug-specific differences, with particular emphasis on the 5FdUR-biased effects on p53-related pathways. The role of TS as a post-transcriptional regulator of p53 and p21 was intended to present as a complementary, exploratory aspect rather than a central mechanistic claim. In the Revised Manuscript, this section has been further de-emphasized, especially in light of additional RNA-seq data, and the corresponding statements have been revised to adopt a more cautious interpretation.

In addition, our related study, which has since been published (DOI: 10.26508/LSA.202503352), provides further context for the observed drug-specific differences. In that work, we demonstrate alterations between these two TS-inhibitory treatments in terms of drug sensitivity, genomic uracilation profiles, and drug-induced mutagenesis in the same cell line model. Notably, that study addresses the time- and dose-dependency at the level of cell viability, revealing reduced sensitivity specifically in response to high-dose 5FdUR treatment, which is also associated with increased cytosine deamination events in genomic DNA. The interpretation of the present transcriptomic findings is now better supported in the context of these previously established drug-specific differences.

In agreement with Reviewer’s suggestions and to strengthen the current study, we have expanded the experimental scope as follows: (1) inclusion of transcriptome analyses from two additional HCT116-derived cell lines with distinct DNA repair capacities; (2) extension of RT–qPCR validation and (3) Western blot analyses to additional drug concentrations.

Taken together, these additions further support our main conclusion that high-dose 5FdUR induces a stronger activation of p53-related pathways compared to RTX. At the same time, we acknowledge that the RNA-binding and regulatory role of TS has not been further investigated. To avoid overinterpretation of this part, we have revised the manuscript to present this aspect more cautiously as a potential, rather than a definite mechanism.

Major and minor points of Reviewer 2

Point 1:

Most of the data is done in MMR-deficient HCT116 model, if I see correct there is no comparison to wild-type HCT116 or other cell lines, making it rather difficult to generalize findings.

Response 1:

We thank Reviewer 2 for this valuable comment. In the revised manuscript, we have expanded our analysis by including additional transcriptome sequencing data from wild-type HCT116 cells, as well as from MMR-proficient but UNG-inhibited HCT116 cells (HCT116-MMR-UGI). Together, these three cell lines represent distinct uracil-DNA repair capacities, enabling a more comprehensive and generalizable assessment of drug-induced transcriptomic changes. The inclusion of these new sequencing datasets, comprising both RTX- or 5FdUR-treated samples alongside their respective non-treated controls, required reanalysis of the entire dataset and the application of updated visualization approaches. As a result, the revised manuscript includes substantial modifications, including new figures and expanded subsections in both the Results and Discussion.

Point 2.

It also looks as if there is no proper vehicle control (solvent only), data are shown to confirm that observed effects are due to the drugs themselves and not general stress. How was the compounds dissolved and given to the cells? This may just need a clarification though.

Response 2:

We thank the Reviewer for this important comment. As the two drugs were dissolved in different solvents (RTX in DMSO, and 5FdUR in water), we have now included an additional DMSO-only control in our Western blot analysis. This control confirmed that the DSMO-treated sample behaved similarly to the non-treated control (c.f. revised Fig 5D and S6 Fig), indicating that the observed effects are attributable to the drugs rather than solvent-induced stress. The corresponding figure legends and methods section have also been revised accordingly.

Point 3.

Most of the experiments rely on single concentration point, this is very risky, for example p53-p21 response could change a lot depending on time and concentration. It would be advised to do dose response curves and also check how p53 and p21 behaves at various concentrations. This is the biggest issue for this referee.

Response 3:

We thank the Reviewer for highlighting the importance of dose- and time-dependent effects, which we agree is a critical aspect of interpreting the cellular responses. We have now directly addressed this point by extending our experimental analysis to include additional drug concentrations.

Guided by our recently published study (DOI: 10.26508/LSA.202503352), in which dose- and time-dependent effects on cell viability were systematically characterized, we selected additional concentration points to evaluate transcriptional and protein-level responses. Specifically, we performed additional RT-qPCR measurements of MDM2, CDKN1A/p21, and CCNG1/cycling following 48 h treatments of HCT116-UGI cells with both low (100 nM) and high (20 µM) concentrations of RTX and 5FdUR (cf. revis

Attachment Submitted filename: Rebuttal_Letter20260404.docx

# Peer review: Decision Letter 1

**DOI:** 10.1371/journal.pone.0332491.r003

**Authors:** Divijendra Natha Reddy Sirigiri

5 Jun 2026

Thymidylate synthase inhibitory drugs induce p53-dependent pathways differently

PONE-D-25-47401R1

Dear Dr. Békési,

We’re pleased to inform you that your manuscript has been judged scientifically suitable for publication and will be formally accepted for publication once it meets all outstanding technical requirements.

Within one week, you’ll receive an e-mail detailing the required amendments. When these have been addressed, you’ll receive a formal acceptance letter and your manuscript will be scheduled for publication.

An invoice will be generated when your article is formally accepted. Please note, if your institution has a publishing partnership with PLOS and your article meets the relevant criteria, all or part of your publication costs will be covered. Please make sure your user information is up-to-date by logging into Editorial Manager at Editorial Manager® and clicking the ‘Update My Information' link at the top of the page. For questions related to billing, please contact billing support.

If your institution or institutions have a press office, please notify them about your upcoming paper to help maximize its impact. If they’ll be preparing press materials, please inform our press team as soon as possible -- no later than 48 hours after receiving the formal acceptance. Your manuscript will remain under strict press embargo until 2 pm Eastern Time on the date of publication. For more information, please contact onepress@plos.org.

Kind regards,

Divijendra Natha Reddy Sirigiri

Academic Editor

PLOS One

Additional Editor Comments (optional):

Reviewers' comments:

Reviewer's Responses to Questions

**Comments to the Author**

1. If the authors have adequately addressed your comments raised in a previous round of review and you feel that this manuscript is now acceptable for publication, you may indicate that here to bypass the “Comments to the Author” section, enter your conflict of interest statement in the “Confidential to Editor” section, and submit your "Accept" recommendation.

Reviewer #1: (No Response)

Reviewer #2: All comments have been addressed

**********

2. Is the manuscript technically sound, and do the data support the conclusions?

The manuscript must describe a technically sound piece of scientific research with data that supports the conclusions. Experiments must have been conducted rigorously, with appropriate controls, replication, and sample sizes. The conclusions must be drawn appropriately based on the data presented.

Reviewer #1: No

Reviewer #2: Yes

**********

3. Has the statistical analysis been performed appropriately and rigorously?

Reviewer #1: I Don't Know

Reviewer #2: Yes

**********

4. Have the authors made all data underlying the findings in their manuscript fully available?

The PLOS Data policy requires authors to make all data underlying the findings described in their manuscript fully available without restriction, with rare exception (please refer to the Data Availability Statement in the manuscript PDF file). The data should be provided as part of the manuscript or its supporting information, or deposited to a public repository. For example, in addition to summary statistics, the data points behind means, medians and variance measures should be available. If there are restrictions on publicly sharing data—e.g. participant privacy or use of data from a third party—those must be specified.

Reviewer #1: No

Reviewer #2: Yes

**********

5. Is the manuscript presented in an intelligible fashion and written in standard English?

PLOS ONE does not copyedit accepted manuscripts, so the language in submitted articles must be clear, correct, and unambiguous. Any typographical or grammatical errors should be corrected at revision, so please note any specific errors here.

Reviewer #1: No

Reviewer #2: Yes

**********

6. Review Comments to the Author

Please use the space provided to explain your answers to the questions above. You may also include additional comments for the author, including concerns about dual publication, research ethics, or publication ethics. (Please upload your review as an attachment if it exceeds 20,000 characters)

Reviewer #1: Not improved

To be rejected

The manuscript is not improved

Still flow less are there

Reviewer #2: Overall, the authors have made a substantial effort to address my major concerns.

As is evident, and expected, p21 has a special regulation which is commonly seen in response to chemotherapeutics at different doses, and will have a major impact on cell fate. Should be noted that p21 calreticulin in translation is hypothetical. The TIFF files have sufficient resolution.The revised manuscript represents a substantial and genuine improvement over the original submission. The work is technically sound, the data are appropriately deposited, and the conclusions are reasonably supported within the stated scope of the study. Since the revision was substantial with lots of new data and text it is good if authors check the manuscript very carefully for minor mistakes at any potential future editing stage.

**********

7. PLOS authors have the option to publish the peer review history of their article (what does this mean?). If published, this will include your full peer review and any attached files.

If you choose “no”, your identity will remain anonymous but your review may still be made public.

**Do you want your identity to be public for this peer review?**  For information about this choice, including consent withdrawal, please see our Privacy Policy.

Reviewer #1: No

Reviewer #2: No

**********

# Editor report: Acceptance letter

**DOI:** 10.1371/journal.pone.0332491.r004

**Authors:** Divijendra Natha Reddy Sirigiri

PONE-D-25-47401R1

PLOS One

Dear Dr. Békési,

I'm pleased to inform you that your manuscript has been deemed suitable for publication in PLOS One. Congratulations! Your manuscript is now being handed over to our production team.

At this stage, our production department will prepare your paper for publication. This includes ensuring the following:

* All references, tables, and figures are properly cited

* All relevant supporting information is included in the manuscript submission,

* There are no issues that prevent the paper from being properly typeset

You will receive further instructions from the production team, including instructions on how to review your proof when it is ready. Please keep in mind that we are working through a large volume of accepted articles, so please give us a few days to review your paper and let you know the next and final steps.

Lastly, if your institution or institutions have a press office, please let them know about your upcoming paper now to help maximize its impact. If they'll be preparing press materials, please inform our press team within the next 48 hours. Your manuscript will remain under strict press embargo until 2 pm Eastern Time on the date of publication. For more information, please contact onepress@plos.org.

You will receive an invoice from PLOS for your publication fee after your manuscript has reached the completed accept phase. If you receive an email requesting payment before acceptance or for any other service, this may be a phishing scheme. Learn how to identify phishing emails and protect your accounts at https://explore.plos.org/phishing.

If we can help with anything else, please email us at customercare@plos.org.

Thank you for submitting your work to PLOS ONE and supporting open access.

Kind regards,

PLOS ONE Editorial Office Staff

on behalf of

Dr. Divijendra Natha Reddy Sirigiri

Academic Editor

PLOS One
